# An update of the goat genome assembly using dense radiation hybrid maps allows detailed analysis of evolutionary rearrangements in *Bovidae*

**DOI:** 10.1186/1471-2164-15-625

**Published:** 2014-07-23

**Authors:** Xiaoyong Du, Bertrand Servin, James E Womack, Jianhua Cao, Mei Yu, Yang Dong, Wen Wang, Shuhong Zhao

**Affiliations:** Key lab of animal genetics, breeding and reproduction of ministry education, College of Animal Science and Technology, Huazhong Agricultural University, Wuhan, 430070 People’s Republic of China; INRA, Laboratoire de Génétique Cellulaire, Castanet-Tolosan, Auzeville-Tolosane, 31320 France; Department of Veterinary Pathobiology, College of Veterinary Medicine, Texas A&M University, College Station, TX 77843 USA; State Key Laboratory of Genetic Resources and Evolution, Kunming Institute of Zoology, Chinese Academy of Sciences, Kunming, China; Faculty of Life Science and Technology, Kunming University of Science and Technology, Kunming, 650500 People’s Republic of China; State Key Laboratory of Agricultural Microbiology, Huazhong Agricultural University, Wuhan, 430070 People’s Republic of China

**Keywords:** Goat, Radiation hybrid map, Genomic rearrangements, Gene duplication

## Abstract

**Background:**

The domestic goat (*Capra hircus*), an important livestock species, belongs to a clade of *Ruminantia*, *Bovidae*, together with cattle, buffalo and sheep. The history of genome evolution and chromosomal rearrangements on a small scale in ruminants remain speculative. Recently completed goat genome sequence was released but is still in a draft stage. The draft sequence used a variety of assembly packages, as well as a radiation hybrid (RH) map of chromosome 1 as part of its validation.

**Results:**

Using an improved RH mapping pipeline, whole-genome dense maps of 45,953 SNP markers were constructed with statistical confidence measures and the saturated maps provided a fine map resolution of approximate 65 kb. Linking RH maps to the goat sequences showed that the assemblies of scaffolds/super-scaffolds were globally accurate. However, we observed certain flaws linked to the process of anchoring chromosome using conserved synteny with cattle. Chromosome assignments, long-range order, and orientation of the scaffolds were reassessed in an updated genome sequence version. We also present new results exploiting the updated goat genome sequence to understand genomic rearrangements and chromosome evolution between mammals during species radiations. The sequence architecture of rearrangement sites between the goat and cattle genomes presented abundant segmental duplication on regions of goat chromosome 9 and 14, as well as new insertions in homologous cattle genome regions. This complex interplay between duplicated sequences and Robertsonian translocations highlights the rearrangement mechanism of centromeric nonallelic homologous recombination (NAHR) in mammals. We observed that species-specific shifts in ANKRD26 gene duplication are coincident with breakpoint reuse in divergent lineages and this gene family may play a role in chromosome stabilization in chromosome evolution.

**Conclusions:**

We generated dense maps of the complete whole goat genome. The chromosomal maps allowed us to anchor and orientate assembled genome scaffolds along the chromosomes, annotate chromosome rearrangements and thereby get a better understanding of the genome evolution of ruminants and other mammals.

**Electronic supplementary material:**

The online version of this article (doi:10.1186/1471-2164-15-625) contains supplementary material, which is available to authorized users.

## Background

The domestic goat (*Capra hircus*) is an important source of food and wool (cashmere) especially important in low input production systems. The ability of goat to consume a wide range of vegetation explains its significance for the agricultural economy. In 2007, the FAO estimated the world population of goats to be around 800 million animals. Despite this economic importance, goat genomic resources are not extensive compared to other livestock species, such as cattle, pig, chicken, or sheep. Goat is a ruminant and a member of the *Caprinae* order, a clade distinct from *Bovinae* in the *Bovidae* family. Its last common ancestor with cattle is dated between 19 and 40 million year (Myr) ago, and with sheep between 6.2 and 8.5 Myr ago [1]. Comparative mapping between cattle and goat using fluorescent in situ hybridization (FISH) showed a strong synteny conservation between the two genomes with a few large-scale rearrangements, including the well-known 9–14 translocation differentiating *Bovinae* and *Caprinae* [2] and differences in intra-chromosomal organization of the X chromosome [3, 4]. However, the history of genome evolution and chromosomal rearrangements on a small scale in ruminants remain speculative, mostly due to limited number of mapped loci.

High-resolution gene maps provide basic but crucial linear information on the physical organization of the genome and typically serve as the backbone for further genomic research. For example, robust information about marker organization on chromosomes is crucial for linkage analysis and marker assisted animal breeding. Although assembling next-generation sequencing data into a draft genome comprising scaffolds is relatively straightforward, constructing a physical map of the structure of chromosomes is still difficult and costly. Recent years have witnessed a slow increase in gene mapping data in the goat since the first genetic and cytogenetic maps [2, 5]. To date, approximately only 550 loci have been mapped to the goat genome using linkage maps of low resolution [6]. The initial release of the goat genome sequence is based entirely on short-read *de novo* sequencing of a Yunnan Black goat that yielded a ~2.66 Gb assembly of 284,683 scaffolds (N50 = 3.06 Mb). Anew improved optical mapping technology was used to joined 2,090 scaffolds into 315 larger super-scaffolds (N50 = 16.3 Mb). Finally, super-scaffolds were assembled into chromosomes based on conserved synteny with cattle [7]. The goat genome sequence could yield a wealth of information about gene content and putative regulatory elements sequences; however, it is still lacking long-range continuity and its released form only gives a fragmented view of the genome.

Another genomic resource, radiation hybrid (RH) maps, played a pivotal role in the process of mapping animal genomes and validation of draft assemblies of the genome sequence, especially in mammals [8–11]. For obtaining dense and accurate next-generation RH maps, new methods were developed recently exploiting the increased availability of comparative genomic resources [12] and have proved successful for the production of RH maps based on SNP array genotyping data [13, 14].

As a part of the goat genome project, we built RH maps of chromosome 1 that helped to validate the draft genome assembly. In this study, using a goat RH panel that we generated recently [15], we constructed robust physical maps of 45,953 loci for all of goat chromosomes except chromosome Y. The whole-genome goat RH map, as an independent chromosomal map, allowed us to anchor and orientate assembled genome scaffolds along the chromosomes thus permitting the annotation of chromosome rearrangements and the study of their evolutionary history using ruminants and other mammalian genomes.

## Results and discussion

### Genotype calling of SNPs in the RH clones

In building the RH maps, we sought to provide accurate, detailed and reliable physical maps at the whole-genome scale to aid in the validation and improvement of the goat genome assembly. Genotyping SNP arrays from other species on the goat RH panel turned out to be a successful strategy for the genotype calling of RH clones. A similar strategy of marker selection from close related species had been applied for constructing buffalo RH maps [16]. Because sequence conservation between the three species considered here is high, a number of bovine and sheep SNPs presented a positive signal in the goat. Moreover, the SNPs that did not provide positive signals were particularly useful for the genotype calling procedure as they gave an internal control, within each clone, of the signal exhibited by non-retained SNPs. We believe it highly increased the robustness of the genotype calling procedure, which is ultimately proven by the quality of the RH maps produced and for future studies we would recommend this approach. The feasibility depends on the availability of a SNP array in a closely related species. To test that whether the approach can be carried out in a species, a simple experiment would consist in genotyping of whole genome sample of the species of interest with the related species array and check that (1) a number of SNPs *can* provide signal and (2) a number of SNPs *do not* provide signal. Optimally both the categories should be in similar proportion on the array. Recall that the fact that SNPs are actually polymorphic in the species of interest is not relevant for RH mapping.

**Figure 1:**
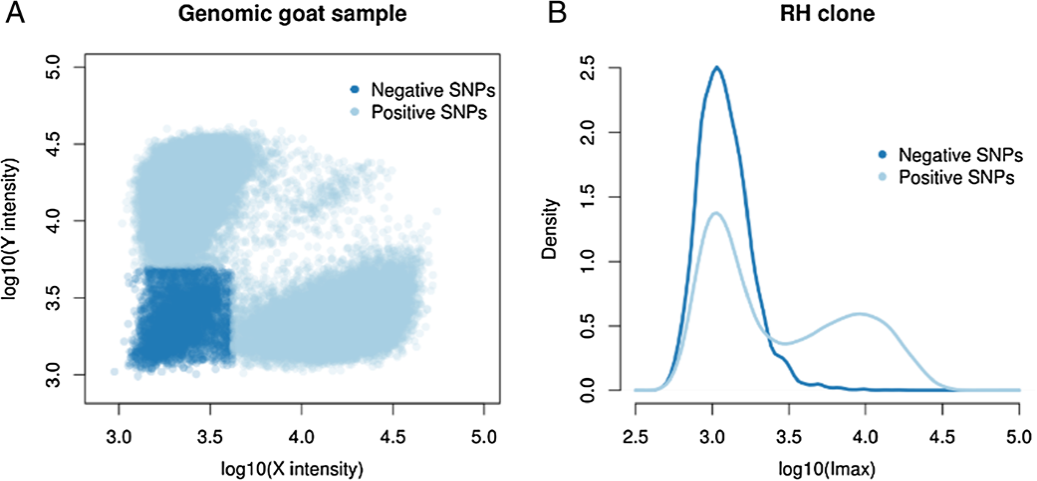
**Imax for SNP marker genotyping.** We called genotypes of clones at SNPs based on the raw signal intensities obtained from Illumina genotyping system. A number (Nneg) of SNPs showed very low intensities for both alleles in the goat (panel **A**). Specifically, the statistic used for genotype calling of a given SNP in a given clone was the maximum observed intensity over the two possible alleles (herein called Imax). The distribution of Imax in a given clone can be seen as a mixture of two underlying distributions, where one for the non-retained SNPs in the clone and one for the retained SNPs (*e.g.* panel **B**).

The goat radiation hybrid panel [12] including 94 hybrid clones was genotyped using IlluminaBovineSNP50K BeadChip and OvineSNP50K BeadChip. RH vectors were constructed for more than 110,000 SNPs out of which 54,318 could be assigned a position on the chromosomes of the goat genome assembly CHIR_1.0. We performed genotype calls (Figure 1) independently for the sheep and the bovine SNP arrays (see Methods). Table 1 summarizes the results of our genotype calling procedure for each SNP array. The two analyzes provided consistent estimates of the panel retention fraction: 37% based on the sheep array data and 35% based on the bovine array data. False negative rates and proportion of missing data were also similar. The call rate was slightly higher for the bovine arrays (80%) than for the ovine array (73%), however, the number of SNPs that could be called was slightly lower (35,521 vs. 43,628). This is explained by the fact that there are much more SNPs (12,577) on the bovine array which do not exhibit a positive signal in the whole genome goat sample than that on the sheep array (3,969), consistent with the phylogeny of these three species.

**Table 1:** **Summary of the genotype calling results**

SNP array	Ns	Nneg	Ndis	Ret. rate estimate	Call rate	Missing proportion	FNR
Ovine	54,231	3,969	6,634	37%	73%	7%	7%
Bovine	54,001	12,577	5,903	35%	80%	5%	5%

Ns: number of SNPs with raw data, Nneg: number of SNPs not callable in the goat, Ndis: number of SNPs discarded based on their retention rate (see details in the text). Call Rate = Proportion of SNP called retained/retention rate estimate. Missing proportion: proportion of missing data, FNR: estimate of the false negative rate in the SNP called “un-retained”.

Even low levels of error result in large losses of information about breakage probabilities, markedly increase the uncertainty in the marker ordering, and inflate estimates of inter-marker distances and total map length [17]. The high error rate of genotypes for constructing certain maps prevents from recovering sufficient signal for ordering the markers. The ability to produce useful robust maps in comparative RH mapping is impaired when the error rate in the data increases above 10% [14]. Before constructing the integrated robust map, we also checked the vector data from bovine SNPs and Ovine SNPs, respectively, and constructed two robust maps for each chromosome. These maps (data not shown) presented a very low error rate in the RH data, in consistent with the low false negative rate estimated by the Q-value R package [18]. However, we note that the slight differences in experimental conditions between genotyping RH clones on the BovineSNP50K array and on the OvineSNP50K array. It could bring certain systematic errors when merging the two datasets, which we cannot assess, since our RH vectors have not been validated by replicated genotyping. However, the availability of negative control SNPs within each clone offers a replication across clones. For increased robustness, we applied three approaches to remove possible genotyping errors before constructing the integrated robust maps.

### Genome map construction and characterization

Briefly, for each chromosome, the analysis of RH mapping was done in three steps:

1. We portioned 53,075 mapped SNPs (mSNPs) according to their assigned chromosome on CHIR_1.0. For each of the 30 chromosomes, a linkage group was established using RH data alone, which removed 13 unlinked markers.
2. We then built comparative RH maps with all remaining markers, using prior information on their ordering from assembly CHIR_1.0. We further excluded 1,918 markers, which possibly have errors or have poor information in RH vector using three criteria (see Methods for details). This resulted in 51,144 SNPs being positioned on 30 chromosomal LKH maps.
3. We extracted a subset of markers from the LKH maps for which the ordering was strongly supported by RH data. This procedure removed about 5,191 SNPs, which could not be confidently ordered, and led to RH robust maps comprising 45,953 SNPs.

**Figure 2:**
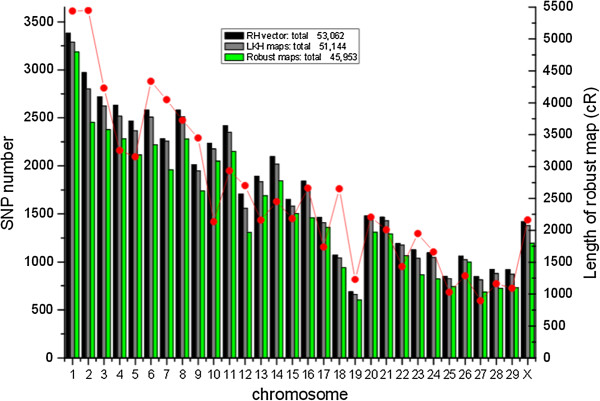
**Number of Single Nucleotide Polymorphisms mapped on the goat chromosomes at different steps of the mapping process and the length of robust maps.**

The final robust RH map contained 45,953 SNP Markers spanning totally 76,800 cR5000. Marker information of the RH maps is shown in Additional file 1. These markers are mapped at 17,628 distinct positions (38.4% of the total number of markers) hence the maps are highly saturated. A summary of the maps including chromosome size, RH map length, and the number of SNPs kept at each step for each chromosome is shown in Figure 2. The number of SNPs removed during construction of robust maps was relatively constant across chromosomes, with the notable exception of CHI23 (27.6%), CHI24 (25.6%), and CHI29 (22.1%), for which a large portion in the middle of the chromosome could not be considered in the robust maps. Marker density was also fairly uniform between chromosomes (results not shown).

**Figure 3:**
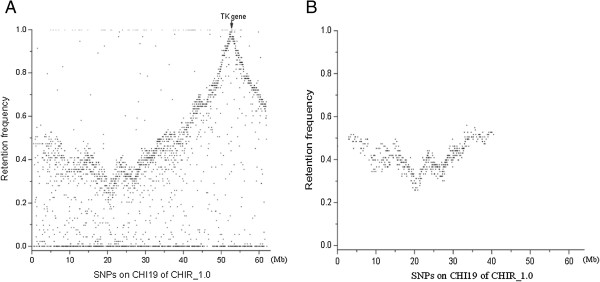
**The retention frequency of the regions which carry the selection marker, thymidine kinase gene 1 (** ***TK1*** **).** Panel **A**: The retention of 2,377 RH vectors in CHI19 of CHIR_1.0, from raw genotyping data before any quality control. Panel **B**: The retention frequency for 604 markers and the position of these markers on the robust RH map of chromosome CHI19. The retention is roughly constant along the chromosome at 39.0%. The missing region in the robust RH map from 40.9 Mb to 62.1 Mb showed extremely high retention. This region carries *TK1* gene at position 52.7 Mb, where the anticipated highest retention near 100% are shown.

**Figure 4:**
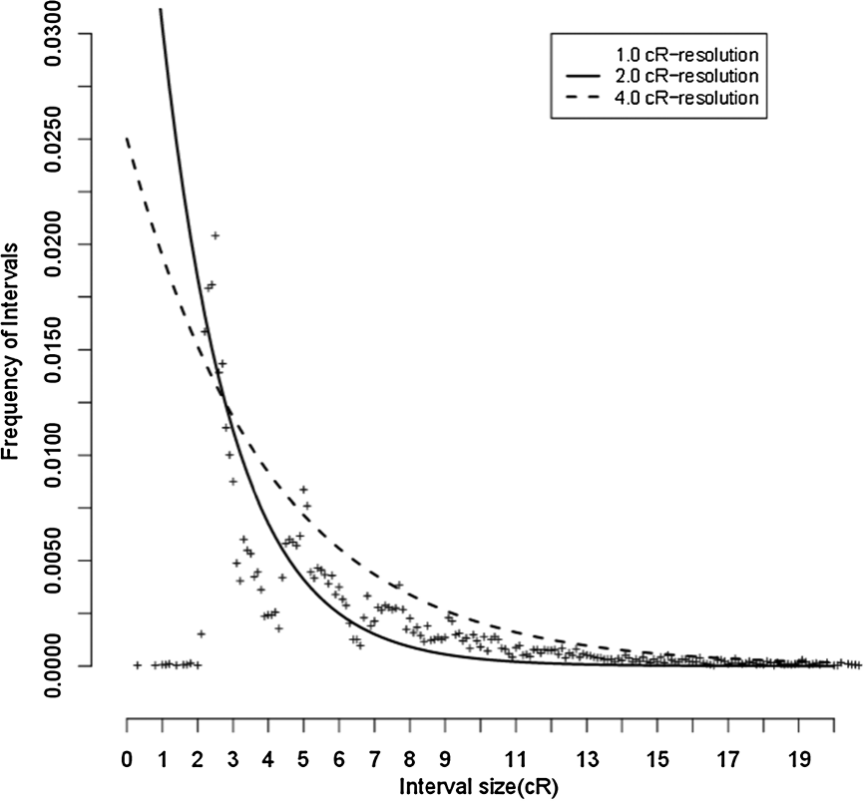
**Observed distribution of inter-marker distances is similar to the expectation for spaced markers at a resolution of 65 kb/2cR.**

Detailed characteristics of the final RH maps are provided in Additional file 2. In general, the retention frequency was stable across the genome, with the expected minor increases at the distal chromosomes. The RH maps uniformly cover >99% of CHIR_1.0 and have an average resolution of 32.6 kb/cR5000 based on a goat genome size of 2.66 GB. The map of chromosome 19 has the shortest length of 1,228 cR and does not cover the region from 40.9 Mb to 62.1 Mb in CHIR_1.0. On CHI19 we retrieved 2,377 RH vectors from raw genotyping data before any quality control. In the raw data, the retention from 40.9 Mb to 62.1 Mb is extremely high because it surrounds the selection locus *TK1* (thymidine kinase 1 gene). We further observed that the regions carry *TK1* gene at position of 52.7 Mb, where the anticipated highest retention near 100%. Consequently, the markers in this region could not be mapped (Figure 3). The log of the ratio of the posterior probability of the best map to the second best map (similar to a LOD score) ranged from 0.17 to 3.54 for robust RH maps. The two chromosomes with the lowest LOD value are CHI21 and CHI4 possibly indicating a higher genotyping error rate in their RH vectors. Genotyping errors are also a vital factor to increase the distance of inter-markers unexpectedly. The total map length of CHI2, CHI7 and CHI18 are higher than expected given the sequence length of these chromosomes (Figure 2). To further characterize the resolution of our RH maps, we studied the distribution of the length of retained fragments using a methodology suggested in a previous study [19]. We found a generally good agreement with the expected distribution of segment lengths (Figure 4), except for the class of very small segments (<2 cR) which is completely lacking in our maps. This is most likely because we are not able to order such close markers with confidence and therefore these small segments are not saw in the robust RH maps. We estimate that the RH panel can order confidently markers separated by at least 65 Kb, which is smaller than the 90 Kb average distances between genes (considering 22,175 genes on a genome of 2.66 Gb).

The main drawback of current high throughput sequencing technologies is the short length of the reads they produce. Many of the novel assemblies including the current goat draft assembly obtained with these approaches are composed of a very large number of scaffolds. This fragmentation does not affect gene discovery, polymorphism analysis and sequence comparison between closely related species but it greatly limits the study of genome structure and chromosomal evolution. The comparative RH approach incorporated the prior knowledge of marker orders in CHIR_1.0 into the statistical model. It has the key property that a different ordering than that of the reference order will be accepted as an alternative order only when the RH data is informative enough. As a consequence, the robust maps allow pinpointing the regions where the assembly order disagrees with an RH map order that is strongly supported by the RH data, so this approach allowed improvements over classical a framework-based mapping strategy [19]. The RH maps presented here offer a powerful complement to the available genetic/cytogenetic maps with very limited resolutions [2, 6, 20] and the genome sequence map [7]. Our RH panel and RH maps also revealed the potential ability to map the unplaced genes or scaffolds and to check possible misassembled regions in further research.

### Validation of the draft genome assembly and anchoring scaffolds on chromosomes

The resolution of discrepancies directly addresses the question of the reliability of the order defined on one side by the genome map and on the other by the assembly. The construction of robust maps was precisely designed to address the reliability of RH maps [14]. In the process of assembling CHIR_1.0, a first draft was produced by standard in silico assembly pipelines yielding numerous scaffolds, followed by an automated optical mapping procedure to assemble scaffolds into super-scaffolds and finally conservation of synteny with cattle was assumed in unassembled regions to organize super-scaffolds into whole chromosomes. For assembling CHIR_1.0, we had constructed two RH maps of chromosome 1 using bovine SNPs and ovine SNPs separately. CHI1 is the longest chromosome and carries many long super-scaffolds. These two maps suggested that the assemblies of super-scaffolds and chromosomes in CHIR_1.0 were accurate [7].

In this study, we merged all the candidate markers from bovine SNPs and ovine SNPs to construct comprehensive RH maps for the 29 autosomes and the X chromosome. The RH marker positions were compared to their sequence positions, providing a comprehensive link between the two physical maps. Based on this link, we aimed at resolving the discrepancies between the two maps to possibly improve the goat genome sequence. Our approach was to consider contigs and scaffolds construction (a small-scale assembly problem out of range given the RH map resolution) as accurate but to allow modifying their organization along chromosomes. More specifically, to resolve discrepancies and update the goat genome sequence using RH maps, we set the priority, from highest to lowest, to the scaffolds, the RH map, the super-scaffolds and the cattle genome successively. Doing so we consider (1) the scaffold data provide ultimate resolution in local region of genome in comparison of any other map, and the assembly of goat scaffold is comprehensively correct at the local level, (2) while the automated whole-genome optical mapping efficiently generated supper-scaffold data for facilitating the anchoring of scaffolds onto chromosomes of CHIR_1.0, the quality control of the maps have not fully been verified, and (3) assuming conserved synteny with cattle should only be required when no other information is available.

**Figure 5:**
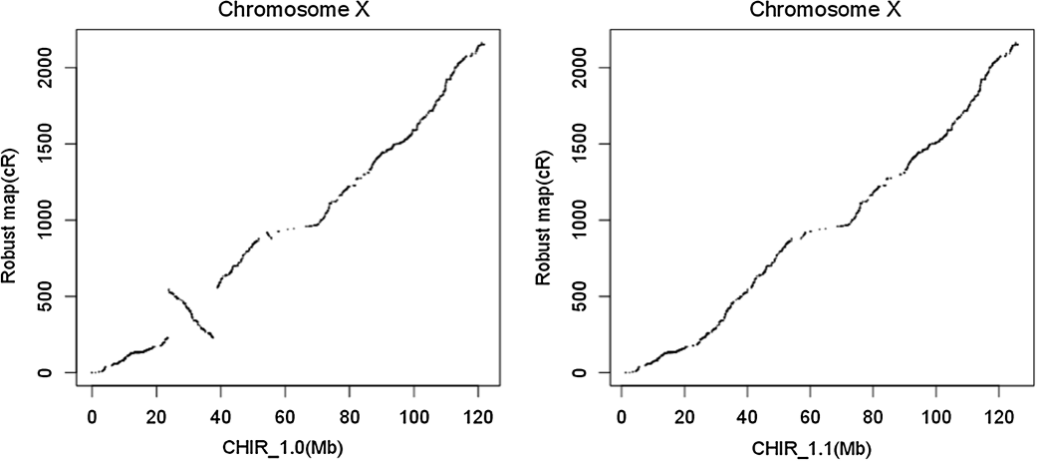
**Comparison between robust RH maps with CHIR_1.0 (left) and CHIR_1.1 (right) for goat chromosome X.**

Markers on the 30 robust maps can be aligned to1,910 scaffolds total 2.47 Gb of the goat genome sequence (Additional file 1). In parallel, the optical mapping for assembling CHIR_1.0 joined 2,090 scaffolds into 315 larger super-scaffolds (N50 = 16.3 Mb). Therefore, these two maps provide together a mean to anchor scaffolds to the whole goat genome. Based on these comparisons we listed a set of proposed improvements to the goat genome assembly, so the modifications of the preliminary assembly (CHIR_1.0) proposed in the present study only involved reordering of scaffolds along chromosomes. In our improved version of the assembly (CHIR_1.1), the positions or/and orientations of 32 scaffolds in CHIR_1.0 were adjusted based on RH maps. 24 scaffolds within 7 super-scaffolds (super3, super240, super102, super152, super192, super93, and super210) and 8 disjunctive scaffolds were reassigned based on the RH maps. As an illustration of such an update, Figure 5 represents assembly order in a region of CHIX in the CHIR_1.0 and CHIR_1.1. For all 30 chromosomes, we provide detailed pictures illustrating comparisons of RH maps with CHIR_1.0 and with CHIR_1.1 (Additional file 3). Our RH maps recovered most of the cattle synteny information, and reassessed the order and orientation of the scaffolds. As explained above, we did not try to correct sequencing data (contigs and scaffolds); however, our approach could potentially contribute to the identification of chimeric scaffolds.

For most markers considered here we were able to assign a position on the genome sequence but still suggest that gaps remain in CHIR_1.0. In chromosome 5 (89.1 Mb between scaffold740 and scaffold666), we underlie a region spanned 72.6 cR haven’t aligned to any goat scaffold data, which mean that a continuous region of over 4.2 Mb missed there in *do novo* assembling of short sequence. Based on predictions using the 17-mer method, the current genome is ~91% of the estimated ~2.92 Gb size. Filling gaps in the goat genome will be a challenge for both technologies of genome mapping and genome sequencing. Since, all maps have some errors in them then it will be important to integrate information from various sources to allow for these checks to be consistent, given that they are generated essentially independently of one another. An approach to resolve contradicting orders is the exploitation of additional and independent source of information. (1) Inconsistency in marker order between the RH maps and other maps is likely due to inaccuracies in independently mappings. (2) Some of the remaining inconsistencies could be biologically grounded, reflecting breed structural variations. Indeed, the reference sequence and the RH panel were generated using the DNA from different individuals and from different breeds (a Yunnan black for the reference sequence and Boer samples for the RH panels), as has already been evidenced in the goat [20].

### Mapping of unplaced scaffolds on the goat genome

As an application of the RH maps, we predicted the positions of SNPs with no localization on the assembly CHIR_1.0. Herein we call these unmapped SNPs “uSNPs”. On the assembly CHIR_1.0, 391 uSNPs assigned to unplaced scaffolds longer than 10Kbhad RH vectors. Firstly, the most likely chromosome was determined based on the linkage with mSNPs and then the best possible position was determined in the LKH map. We excluded uSNPs on tiny scaffolds from map construction because of the limited resolution of our RH maps (we noticed that essentially all SNPs assigned to a given scaffold had consistent predicted localization on the same RH map). We identified 32 uSNPs (2.2%) on the X chromosome, more than expected compared to autosome maps. This could imply that for CHIX more genome sequence is missing in the current assembly than for other chromosomes. We set the same principle for mapping unplaced scaffolds as we used to validate the draft genome: unplaced scaffolds could only be inserted between two contiguous placed scaffolds and we aligned the candidate scaffolds and their neighbors based on the cattle genome order.

**Figure 6:**
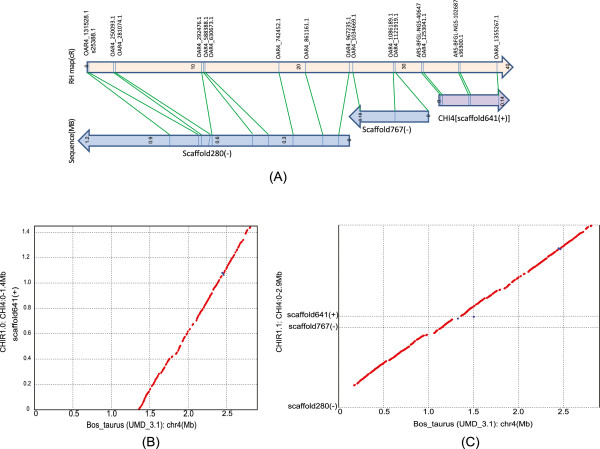
**The validation of the predicted position for the unplaced scaffold280 and scaffold767.** Panel **A**: We constructed a local RH map of CHI4 including SNPs from the scaffold the unplaced scaffold280 and scaffold767. Panel **B**: The genomic region from 0 to 1.35 Mb on cattle chromosome 4 do not have any alignment with CHI4 on CHIR_1.0, which pointed out that a long sequence probably missed on the distal of CHI4 on CHIR_1.0. Panel **C**: We verified that scaffold280 and scaffold767 were aligned with the putative syntenic region of the cattle genome and were closed to the placed scaffold641.

Localizing unplaced scaffold280 (1,268 Kb with 9 uSNPs) and scaffold767 (185 Kb with 3 uSNPs) is illustrated on Figure 6. The predicted localization for this scaffold, based on SNPs predicted positions, was at the beginning of CHI4. We verified that scaffold280 and scaffold767 aligned on the putative syntenic region of the cattle genome and constructed a local RH map of the region including uSNPs of both scaffolds. We found that both scaffolds were indeed strongly linked to scaffold641 on CHI4 so that both can be placed on the goat genome confidently. Another example, illustrated in Additional file 4, revealed that RH maps can be used to improve the goat sequence map accurately in a local region by both correcting the ordering of scaffolds and adding unplaced scaffolds.

Among the uSNPs for which we could assign a position on the goat genome, we were able to align 95 scaffolds present in the goat genome sequence but currently unassigned. Of these scaffolds 52 only could be assigned a position but not orientated, either because they carried a single uSNP or because they contained multiple tightly linked SNPs whose map order was questionable due to insufficient radiation-induced breakpoints. The orientations of the 53 scaffolds were obtained using comparative information with the cattle genome (UMD3.1) assuming conserved synteny. In total, 19.45 Mb of previously unplaced genome sequences were incorporated into the new version of the genome assembly. The comparison between the RH map with super-scaffolds and the CHIR_1.1 assembly are presented in Additional file 5. Of 1,910 scaffolds that can be aligned to the 30 robust RH maps, 1,884 were used to reassembling chromosome sequences. Only a few rearrangements exist between RH map and the new version of genome assembly. The updated assembly is available as an agp file (the CHIR_1.1 assembly, at http://goat.kiz.ac.cn)

### Genome rearrangements between caprine and bovine genome

We used our RH updated genome assembly to identify conserved segments between species that led to novel discoveries of ancient chromosome rearrangements and study the sequence features of evolutionary breakpoints. Mapping a large number of markers on the goat genome and cross-referencing 96.4% of these with the map locations for the markers in the bovine achieved this alignment (Additional file 1). Overall, there is a very good colinearity between the goat RH maps order and the bovine assembly order, consistent with a relatively short divergence time between the two species. However there are few discrepancies (Table 2), in agreement with chromosome banding features [21]. X chromosomes appear to have been subjected to large number of rearrangements, compared to the autosomes, suggesting that rearrangements might better tolerated on sex chromosomes. The X-chromosome linkage group is usually conserved in placental mammals and does not interplay with autosomes in chromosome evolution. Comparison of the cytogenetic maps of caprine and bovine X chromosomes also shows some changes in loci order, defining 6 common chromosome segments [3, 22]. Both the RH map and the sequence map confirmed the well-known chromosome 9–14 translocation, with CHI14 containing a small BTA9q11-q13 segment including genes COL9A1 and ETH225 [23]. Our comparative mapping did not detect inversions between CHI2 and BTA2, or CHI19 and BTA19, which were suggested by these previous cytogenetic maps. We believe the relative inaccuracy in these cytogenetic maps may account for these differences.

**Table 2:** **The major rearrangement sites between goat and cattle**

Goat/chrom.	SNP marker	RH(cR)	Goat_V1.1(Mb)	UMD3.1(Mb)
Chr14	OAR9_334753.1	0	chr14:0.41	chr9:12.20
	OAR9_12923703.1	269.51	chr14:11.67	chr9:0.90
Chr13	s60476.1	259.30	chr13:10.31	chr13:16.44
	s10077.1	433.42	chr13:15.86	chr13:10.59
ChrX	OARX_91845722.1	0	chrX: 1.46	chrX:27.32
	ARS-BFGL-NGS-41828	119.73	chrX: 21.42	chrX:40.41
ChrX	OARX_111907035.1	152.9	chrX: 25.88	chrX:6.74
	OARX_126406510.1	254.23	chrX: 35.07	chrX:0.34
ChrX	OARX_120191387.1	268.01	chrX:36.52	chrX:7.10
	ARS-BFGL-NGS-8011	625.00	chrX: 56.40	chrX:23.69

**Figure 7:**
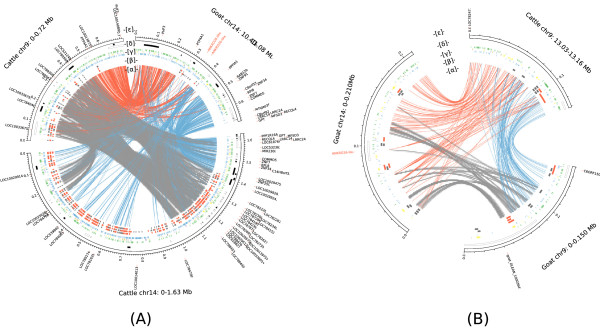
**Sequence architecture at synteny breaks of chromosome 9–14 translation between cattle (UMD3.1) and goat (CHIR_1.0).** Panel **A**: A breakpoint region on CHI14 (10.40 Mb-11.08 Mb) mapping to two cattle centromeres. Panel **B**: A breakpoint on BTA9 (13.03 Mb-13.16 Mb) mapping to two goat centromeres. In each panel, [α] Goat-cattle DNA pairwise alignments are highlighted using colored curve; Self-comparisons of goat regions or cattle regions are grey. Each two neighboring synteny blocks are distinguished between in red curves with in green curves, respectively. Colors have not any biological meanings. [β] Segmental duplications longer than 3 kb are highlighted in grey (>5 kb are in red). Long segmental duplications are highly enriched in breakpoints comparing to synteny blocks. [γ] LINEs (green), SINEs (purple), Simple repeats (blue), and LTRs (yellow). [δ] Genes with colors denotes transcriptional orientation (the white represents “+” and the black represent “-“). [ϵ] Names of ANKRD26 homologsare marked in red. Breakpoint intervals were highlighted using mixture colored regions from two neighboring synteny blocks between cattle and goat, which revealed mosaic new insertions at rearrangement sites. Self-comparisons showed that these two centromeres share larger homologous region in cattle (panel **A**), different from in goat (panel **B**) suggest two cattle centromeres underwent a much longer evolution process than two goat centromeres. The breakpoint in goat genome (panel A) presents more complex architecture than one in cattle genome (panel **B**). Segmental duplications are enriched in breakpoints comparing to synteny blocks suggested that in these cases duplications promoted nonallelic homologous recombination (NAHR), and thus a chromosome rearrangement.

**Figure 8:**
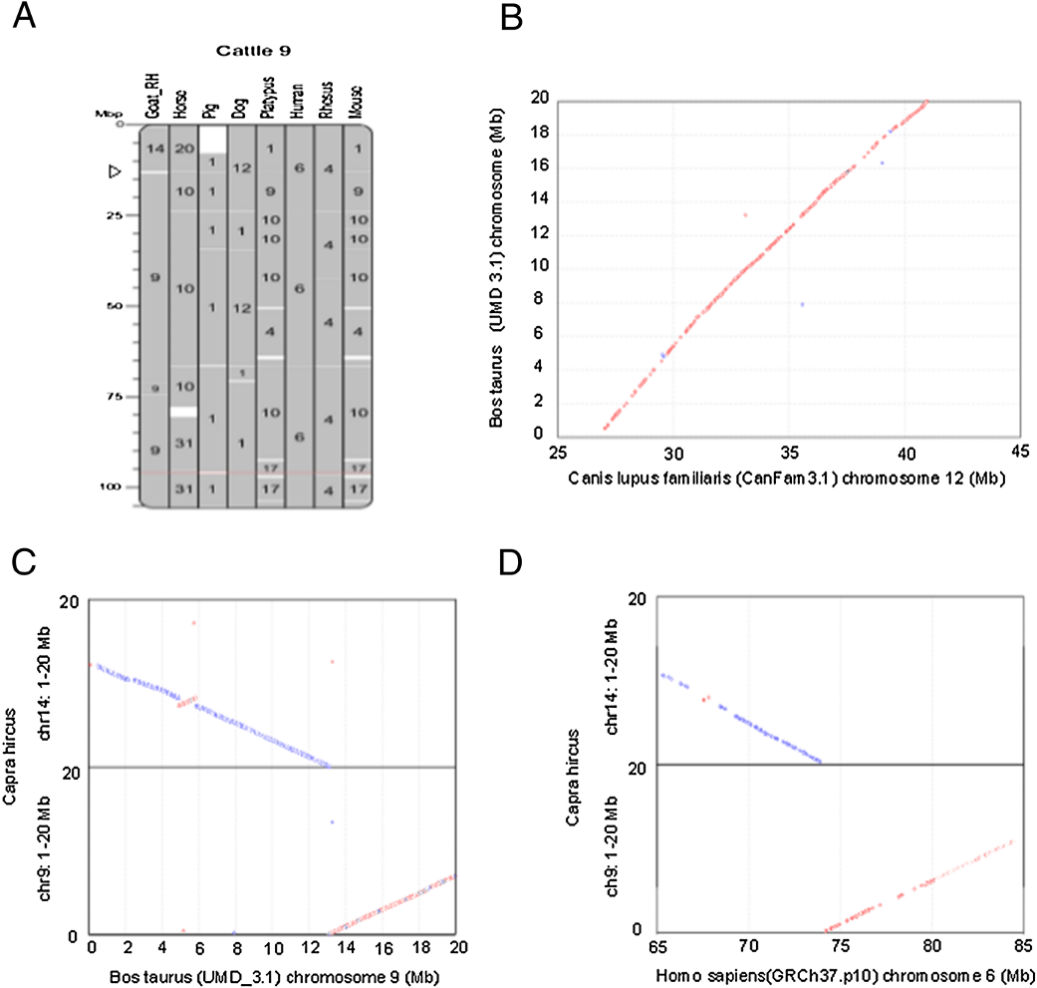
**Cattle share HSB with human and dog genome in at position 13.0 Mb on cattle chromosome 14 (panel A and panel B), but synteny block break on goat genome (panel C and panel D).**

We characterized the sequence architecture of the evolutionary breakpoints for two ruminant rearrangements, one between CHI9 and CHI14 (Figure 7) and one in CHI13 (Additional file 6). While the CHI9-CHI14 translation is a known rearrangement between ruminant genomes [6], the CHI13 one was not described previously. Cattle shares a large region of conserved synteny with the human and dog genomes at position 13.0 Mb on cattle BTA14, whereas goat presents species-specific breakpoints and loaded to two centromeres of CHI9 and CHI14 (Figure 8), so we considered that the rearrangement occurred in the goat lineage. Breakpoint intervals from two neighboring synteny blocks between cattle and goat reveal a mosaic of new insertions at rearrangement sites, rather than a simple model of nonhomologous end-joining (NHEJ). In Figure 7, Representative repeat elements including LINEs, LTRs, SINEs, simple repeats and segmental duplications (SDs), as well as genes are enriched in breakpoints compared to synteny blocks. Each breakpoint region aligns with the two homologous centromeres radiantly and is sprayed with SDs. More interestingly, the breakpoint of the goat genome (Figure 7A) presents a more complex architecture than cattle genome one (Figure 7B). We inferred that these two cattle centromeres underwent a much longer evolution of SD than that of the two goat centromeres (Figure 7A and B). It suggests that these two goat centromeres of CHI9 and CHI14 are formed by a Robertsonian fusion and is evolutionarily more recent. Though neocentromere formation may be independent of sequence characteristic and comply with the rules of epigenetic, DNA rearrangement is one of the primary driving forces for neocentromere formation in chromosome evolution. Abundant SDs could hypothetically provide homologous sequences that are not in allelic positions, where nonallelic homologous recombination (NAHR) or crossing-over in meiosis result in region-specific genomic rearrangement [24]. These duplications can promote NAHR, and thus a chromosome rearrangement. Among distinctive sequence features, we noted that a gene cluster containing ANKRD26 (ankyrin repeat domain 26) was highly duplicated in ruminant breakpoints (Figure 7 and Additional file 6).

The ancestral Bovidae diploid number of 30 pairs of chromosomes has been retained in all but one (gaur) of the previous studied species of Bovina. Robertsonian fusions or centric translocations (ROBs) involving the fusions of two nonhomologous acrocentric or telocentric chromosomes at their centromeres (producing a single biarmed product with distinguishable internal centromere) is frequent in ruminant chromosome evolution and particularly in *Bovidae* [25–27]. Several hypotheses have been advanced to explain the formation of ROBs in mammals usually invoking the organization of pericentromeric satellite DNA and nonallelic homologous recombination at meiosis [28]. Breakpoints are considered to occur in regions of SDs [29] and high concentrations of repetitive elements [30]. The sequence architectures of genomic breakpoints what we identified for ROBs are found to be complex with enriched genes and other active elements, which refine the evidences of these previous studies. The fact that SDs are enriched in breakpoints compared to synteny blocks and that breakpoint specific SD have a different evolutionary history in goat and cattle suggests that in these cases duplications promoted nonallelic homologous recombination (NAHR), and thus a chromosome rearrangement.

### ANKRD26 gene cluster associated with genome rearrangements

Large-scale genome rearrangements caused by chromosome breaks underlie numerous inherited diseases and are associated with species evolution. Evolutionary breakpoints are nonrandomly distributed (reused) throughout mammalian genomes [31] and many breakpoints are coincident with ancient centromere activity as well as new centromere formation [32–36]. It has been hypothesized that centromeric illegitimate recombination between nonhomologous chromosomes led to chromosome fusions and synteny breakpoints during rapid speciation. This hypothesis initiated from the analysis of karyotypic evolution in mammals [37] and was supported by similar studies in marsupials [32] and in plants [38].

**Figure 9:**
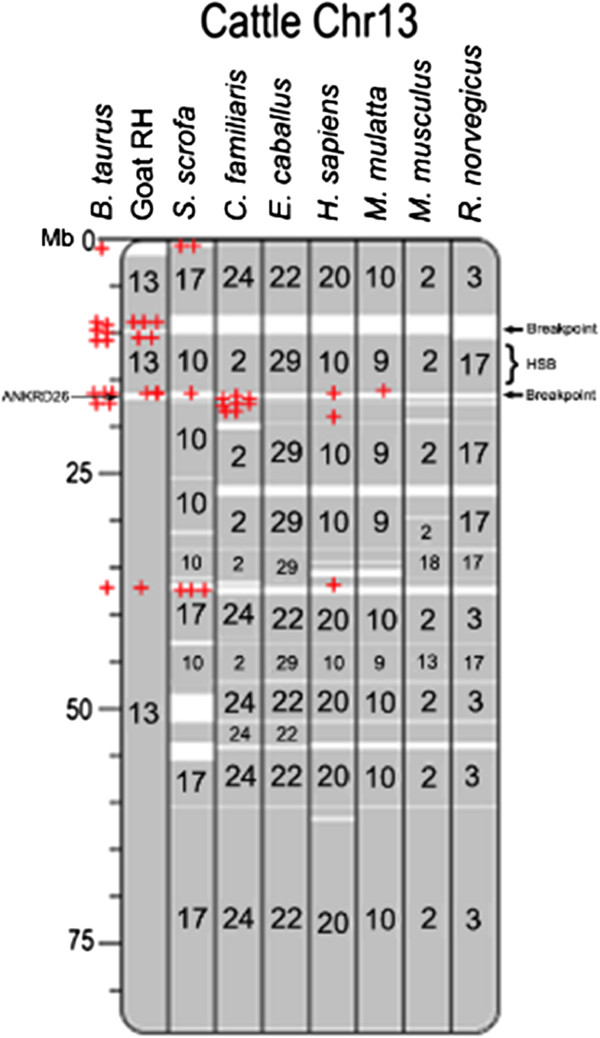
**An example of the map of mammalian breakpoints and ANKRD26 homologs.** Cattle chromosomes are used as reference chromosomes and are respectively compared to goat RH maps, pig (*S. scrofa*), horse (*E. caballus*), dog (*C. familiaris*), human (*H. sapiens*), macaque (*M. mulatta*), mouse (*M. musculus*), and rat (*R. norvegicus*) in the Evolution Highyway program. Large blocks of homologous synteny and a high frequency of breakpoint reuse are presented on mammalian chromosomes. We combined of this map of mammalian breakpoints and the genomic distribution ofANKRD26 family (Additional file 8) and take examples of cattle chromosome 13 in this figure. Most members of ANKRD26 family locate on species-specific or lineage- specific rearrangement regions or centromeres (red cross), whereas a very few (blue cross) were located on HSBs. The homologous copies of ANKRD26 are spotted intensively in two neighboring breakpoint regions, where ANKRD26 locate. One likely reason for of this excessive gene duplication is that the HSB between the two neighboring breakpoints have undergone repeatedly inversion, thus ANKR26 copies accumulated.

From goat-cattle the comparison, we found that ANKRD26 has expanded in ruminant breakpoints. We then explored the genomic distribution of members of ANKRD26 homologs to other mammalian genomes. The ortholog of ANKRD26 is a highly conserved protein gene in reptiles, birds and mammals. We found that ANKRD26 have a single copy in each reptile genome or each bird genome as far as we can find in their available genome sequences, whereas it was expanded in mammals. Phylogenetic analysis of ANKRD26 homologs revealed four groups of homologous genes including ancient ANKRD26-like (1), POTE (2), ANKRD18/ANKRD20 (3), and ANKRD36/ANKRD62 (4) and an over-expansion especially in primate and artiodactyla (Additional file 7). The analysis of evolutionary breakpoints using the Evolution highway program [33, 39, 40] was used to determinate whether a breakpoint is species-specific or lineage-specific (i.e. reused). We combined of the map of mammalian breakpoints and the genomic distribution ofANKRD26 homologs (Figure 9 and Additional file 8). The result showed that most of ANKRD26 homologs locate on species-specific or lineage- specific rearrangement regions or centromeres, whereas a very few (6/115) were located on homologous synteny blocks (HSBs). For instance, the POTE family, made of primate-specific paralogs of ANKRD26, was extensively reshaped involving segmental loss and internal duplication in human [41]. Genes in the POTE family are located near the centromeres of numerous chromosomes including the site of human-chimpanzee chromosome 2 fusion [42]. Of the 22 human autosomes, 11 centromeres including all five acrocentric ones (that always resulted from Robinson translocation) have ANKR26 paralogs. The ANKRD26, as the ancestor of this big family, is located on cattle (or goat) chromosome 13 and human chromosome 10p, and its homologous copies are spotted intensively in two neighboring breakpoint regions. One likely reason for of this excessive duplication is that the HSB between the two neighboring breakpoint have undergone repeatedly inversions, thereby provoking an accumulation of ANKR26 (Figure 9 and Additional file 6). This means that species-specific shifts in ANKRD26 duplication are coincident with breakpoint reuse in divergent lineages.

Evolutionary breakpoints are supposed to contribute importantly to new genetic variation and novel genes at these fragile sites [39]. However, the dynamics between evolutionary breakpoints and genomic elements locally have not been completely understood yet [43]. A current hypothesis is that genetic innovation and change at these sites may, as a driving force of local adaptation [44], have influences in subsequent chromosome stabilization. Indeed, most of the proteins belonging to this gene cluster conserved the Ankyrin domains near to N-terminal and the structural maintenance of chromosomes protein (SMC) domains near the C-terminal (Additional file 9). SMC proteins interact with DNA in chromosome condensation, sister-chromatic cohesion, recombination, DNA double-strand break repair and epigenetic silencing of gene expression. It may indicate that ANKRD26 is responsive to DNA damage [45], pericentromeric cohesin and condensing [46], and even centromere formation. The members of ANKRD26 usually linked with LINEs near the C-terminal [47] may indicate their duplication by retrotransposition.

Although little is known about the regulation of ANKRD26, previous studies demonstrated that ANKRD26–involved in chromosome rearrangements are responsible for several inherited disease: a gene conversion of ANKRD26 is associated with type-2 diabetes [48], which were supported by the studies of ANKRD26-inactivation [49] and familial thrombocytopenia, associated with a chromosome breakage [50] on human chromosome 10p [51] appears to be due to an ANKRD26 mutation [52, 53].

## Conclusions

In the present study, we constructed dense goat RH maps of SNP markers from closely related species via a novel method of genotype calling and recent methodologies. Overall, there is good agreement between the current genome assembly and the robust RH maps. We were able to propose correction to the goat genome assembly and add previously unplaced scaffolds. We combined this information in the form of a new goat genome sequence CHI_1.1.We think this updated sequence, with a more accurate ordering of scaffolds will can be useful for many genetic studies on goat, for instance, linkage disequilibrium analysis and QTL mapping. We used our new dense chromosomal maps to annotate genomic rearrangements and chromosome evolution of ruminants. We compared sequence architecture of the evolutionary breakpoints including representative repeat elements and genes in ruminant rearrangements. Breakpoint intervals from two neighboring synteny blocks in goat gnome reveal a mosaic of new insertions at rearrangement sites. The Breakpoints in the goat genome presents more complex architectures than breakpoints of the cattle genome suggesting that goat centromeres of CHI9 and CHI14 underwent much shorter evolutionary time than those two cattle centromeres. Our findings highlight the complex interplay between duplicated sequences and chromosomal rearrangements, which favored a rearrangement mechanism of centromeric non-allelic homologous recombination in mammals. We detected a gene cluster, ANKRD26, which was expanded specifically in species as well as lineage specific evolutionary breakpoints and in centromeres. Their genomic distribution associated with the biological function of their conserved protein domains suggested it might play a role in chromosome stabilization during chromosome evolution.

## Methods

### RH panel and marker genotyping

We generated a 5000 rads goat-hamster RH panel [15]. In total, 108,242 *Bovidae*-derived SNP markers from BovineSNP50K BeadChip (54,001) and OvineSNP50K BeadChip (54,241) were genotyped against 94 RH clones and 2 controls (one positive Boer goat sample and one negative hamster A23 sample) using the IlluminaBeadStation 500G genotyping system.

We performed the genotype calling procedure described below independently for each SNP array. We called genotypes of clones at SNPs based on the raw signal intensities obtained from the Illumina genotyping system. Specifically, the statistic used for genotype calling of a given SNP in a given clone was the maximum observed intensity over the two possible alleles (herein called Imax). The distribution of Imax in a given clone can be seen as a mixture of two underlying distributions, where one for the non-retained SNPs in the clone and one for the retained SNPs (*e.g.* Figure 1B). The difficulty of the genotype calling procedure is to evaluate from which of these two distributions a given data point (Imax) comes from. More precisely, we aimed at evaluating the p-value of the observed Imax under the hypothesis H0 that the SNP is not retained in the clone (i.e. P (T > =Imax |H0)).To calculate this p-value an estimate of the distribution of Imax for a non-retained SNP is needed. For this, we exploited the fact that the SNP array was designed for a different species than the goat (either cattle or sheep). Looking at the intensity distribution in the positive whole genome goat sample, we observed that a number (Nneg) of SNPs showed very low intensities for both alleles in the goat (Figure 1A). We speculate that the sequences corresponding to these negative SNPs are not conserved enough in the goat genome to provide a signal with the SNP array. Finally, the p-value that a (non-negative) SNP is not retained was calculated as the proportion of Imax in the negative SNPs that exceeds the observed Imax. If none was found, we set the p-value at 1/Nneg.

After calculating p-values for all observations, we estimated their corresponding q-values using the Q-value R package [18]. This approach allows controlling the false positive and false negative rates and provides an estimate of the proportion of true null hypotheses in the data, which is in our case an estimate of one minus the panel retention fraction. We called "retained" data points at an FDR threshold of 1% and removed SNPs which retention fraction was either greater than 50% or lower than 10%. Furthermore, one RH clone (#73) showed a very high rate of retention (>90%) and was therefore removed from the analysis. Among the remaining data points, we called “missing” data points with q-values lower than 10% for the sheep array, and 5% for the bovine array. Finally, data points with q-values greater than 10% were called “un-retained”. The overall approach with these thresholds allowed us to control both the missing data rate and the false negative rate (see Results).

### Map construction

Our pipeline of RH mapping is very similar to that recently used for recently building porcine RH maps [13]. The Carthagene software [12, 14, 54] was used to compute efficiently marker ordering for candidate maps with reference maps. This approach is based on a probabilistic Bayesian model integrating the usual RH probabilistic model with a probabilistic model of breakpoint occurrences with the reference order. In this probabilistic model, breakpoints induced by chromosomal rearrangements are considered as rare events, following a Poisson law. Meaning, the model considers that genome assembly errors create rare spurious breakpoints between the RH map order and the current goat genome assembly order [12].

The map construction process was conducted in parallel to the goat genome assembly. As our first goal to build RH maps was to help ordering scaffolds of the goat genome, only SNPs that could be mapped unambiguously to a working draft of the goat genome were used to build RH maps. These summed up to 54,318 SNPs, with 35,203 SNPs from the Ovine array and 19,115 SNPs from the bovine array. We portioned 53,075 mapped SNPs (mSNPs) according to their assigned chromosome and 1243 unassigned SNPs (uSNPs) remained on assembly CHIR_1.0. For each autosome, we determined linkage groups among mSNPs, based on RH data alone. Of the 53,075 SNPs that generated RH vectors, 53,062were assigned to 30 single linkage groups based on two-point analysis at a LOD threshold of 10. In these linkage groups 13 markers did not show enough evidence for linkage.

Each RH linkage group was assigned to a chromosome based on homologous comparison between RH markers and the goat genome. (1) We manually increased the LOD threshold in a two-point analysis to remove minor marker groups (<5 markers) to remove possible error vector. (2) A primary map was first generated with Carthagene by converting the RH data into a TSP and solved using a LKH heuristic method (*lkh*1 1). The initial RH maps were compared to the assembly to observe the general discrepancies, and were used to inspect inter-markers intervals with extremely long distances (>25 cR). In this step, we manually removed possible questionable markers, most of which were located on map ends. (3) The command of find_errors in *RHMAPPER* [19] was used to flags marker/hybrid assay results that are likely to be the result of laboratory error, where the order for these results set to the primary map. A threshold of 3 was specified in the command *i.e.* reporting that this result is 1000:1 more likely to represent a laboratory error than a true result. Once again, we converted the RH data into a TSP and solved using the LKH heuristic method, and constructed the LKH map.

A novel approach [14] was applied next to build maps with a highly reliable ordering, called robust maps. Briefly, the principle of the method is to estimate a posterior distribution of marker ordering using an MCMC approach and then to extract from this distribution a subset of markers that show the same ordering across maps of high probability. We performed 5000 MCMC iterations and discarded the first 1000 as burning iterations in the command of Carthagene software, *mcmc*, using the LKH maps obtained above as starting orderings. We extracted robust maps from the posterior distribution using the metamap software [14]. An inclusion tree called *metamap* that summarizes the uncertainty lying in the map distribution was calculated. In the *metamap* tree, groups in which the best order have posterior probability higher than 95% in the robust map were include the final robust map. The distances of the robust maps were evaluated using the diploid equal retention model with an EM convergence set to 10^−9^ (command *cgtolerance*). This approach is closely related to the idea of constructing framework maps. In the case of framework maps, an order is accepted based on a maximum likelihood ratio, also called LOD (the logarithm of odds between the best order and the second best order must exceed a preset ratio, such as 1,000:1 for example (LOD 3)). In contrast, the construction of robust maps falls into the Bayesian paradigm [14].

For mapping the unplaced scaffolds on the goat genome, we constructed primary LKH maps with mSNP and uSNP together. We removed 852 uSNPs, which assigned to scaffolds shorter than 10 Kb in RH mapping from 1,243 unassigned SNPs (uSNPs) on assembly CHIR_1.0. Then 391 uSNPs were assigned to chromosomes based on their linkage with each of 30 linkage groups. The LKH maps were constructed as above. We aligned both of the robust maps of only mSNP and the LKH map of all SNPs to the goat scaffold data, then computed the orientation of scaffolds positions on the chromosomes and the orientation of the scaffold. A comparison of the two results showed that the assignments of placed scaffolds are very similar, so we proposed that the assignments of placed scaffolds using the LKH maps are accurate.

### Genome map comparisons and gene comparisons

Map discrepancies were determined by comparing the RH map with target genome data including goat scaffold sequence, goat assemblies (CHIR_1.0 and CHIR_1.1) and cattle genomes (BTA4.2 and UMD3.1). The probe sequences of markers in RH maps aligned with these target genome maps using BLASTN (version 2.2.21) with E-values < 1 × 10^−10^. We also conducted synteny-based comparisons between the goat genome sequence and other mammalian sequences. The comparisons were performed using NUCmer [55] with parameter “-c 500” (with cattle) or “-c 150” (with other mammals). The assignment of assembled sequences to chromosomes was done based on a best alignment and majority rule. The Interquartile Range method was used to filter out suspicious markers and compute the scaffold positions on chromosomes. The Least Square method was used to compute the orientation of the scaffold that had multiple markers aligned to them. Orientation was considered forward if the slope value was positive, reverse if the slope value was negative and unknown if the slope was close to be 0.

For the reconstruction of the evolutionary breakpoints, the Evolution Highway program [33, 39, 40] was used to compare more mammalian genomes including human, mouse, dog, horse, cattle, rat and goat RH maps. The data of orthologous gene pairs between cattle genome and other mammalian genome for generating homologous synteny block was downloaded from the Biomart database. We used lastz program and lastz-based Cassis program [56] to precisely detect sequence homology and genomic rearrangement breakpoints. RepeatMasker was run against both libraries separately to identify homologous repeats, which were classified into known classes of repeats [57]. SDs was identified using the identical whole-genome sequence comparison method [58]. The characterized elements of sequence were graphed by circus [59].

For phylogenetic analysis of gene family, the sequence of goat ANKRD26 was blasted (BlastP) against the NCBI-nr database. In the results, genes with less than 40% homology or 40% coverage were filtered out. The annotated genes of ANKRD26 in public protein databases also were retrieved. Multiple sequence alignment was performed and the Neighbor-joining tree were constructed based on Dayhoff PAM matrix method with a bootstrap value of 1000 [60].

## Electronic supplementary material

Additional file 1: **Radiation hybrid maps of the Illumina SNP markers.** This text file contains the position (in centiRays) on final robust RH maps of 45,953 SNPs from Illumina BovineSNP50K BeadChip and OvineSNP50K BeadChip on radiation hybrid maps. The RH markers were mapped to goat assemblyCHIR_V1.0, cattle genome assembly BTA4.2, goat assembly CHIR_V1.1, goat scaffolds and goat super-scaffolds, respectively. (TXT 4 MB)

Additional file 2: **Characteristics of the RH maps.** The table contains detailed characteristics of the RH maps obtained for each chromosome: number of SNPs mapped; number of distinct position for each panel, resolution, average marker distance and the retention fraction. (XLS 40 KB)

Additional file 3: **Detailed comparison of RH maps with the CHIR_1.0 and with the CHIR_1.1.** This file contains comprehensive pictures comparing (i) CHIR_1.0 and the RH maps, as well as (ii) the goat genome sequence CHIR_1.1 and the RH maps. (PDF 348 KB)

Additional file 4: **Our RH maps can be applied to improve goat sequence map accurately in local region, by both of rectifying the arrangements of scaffolds and adding the unplaced scaffolds.** Forward alignments are plotted as red lines/dots while inverse (reverse compliment) alignments are plotted as blue lines/dots. Panel **A**: The region from 8.3 Mb to 8.9 Mb of CHI14 [where the order of scaffolds is scaffold406 (−), scaffold940 (+), scaffold1516 (−)], showed an inversion and two deletions with cattle genome, that presents either true chromosome evolution or artificial errors. Panel **B**: The RH map suggested an order of scaffolds in the region [scaffold1942(+), scaffold1305(−), scaffold940(−), scaffold406(+), scaffold1542(+), scaffold1424(−), scaffold1896(+), scaffold2162(+), scaffold1331(+), scaffold1733(−), scaffold1187(+), scaffold2528(−), scaffold1516(−)]. The new sequence, ordered using our RH map showed good colinearity with the cattle genome, demonstrating that the inversion and the two deletions were artificial errors. Thus, the goat sequence can be improved. Panel **C**: The cattle genome suggested an order of scaffolds in the region. The information of conserved syntenies is used to the new order of scaffold by RH map. Panel **D**: The new ordering of scaffolds excluded three dubious scaffolds (scaffold1424, scaffold1896, and scaffold2162) and is used to the assembly CHIR_1.0. (PDF 200 KB)

Additional file 5: **The connections between the robust RH maps with sequence-based scaffolds and super-scaffolds.** Markers in the 30 robust RH maps were linked to 1884 scaffolds and then to 297 super-scaffolds, that were used to reassembling chromosome sequences. Only a few rearrangements (the red link lines between RH markers with chromosome sequences) exist between the RH map and the new assembly CHIR_1.1. Forward alignments between scaffolds and chromosome sequences (CHIR_1.1) are plotted as red lines/dots while inverse (reverse compliment) alignments are plotted as blue lines/dots. Forward alignments between super-scaffolds and CHIR_1.1 are also plotted. To better display the connection, each of neighboring scaffolds (or super-scaffolds) are compulsively divided to two paralleled lines. (PDF 342 KB)

Additional file 6: **Sequence architecture at synteny breaks in chromosome 13 between cattle and goat.** [α] Goat-cattle pair-wise alignments are highlighted by red and green colors; Self-comparisons are in grey. [β] Segmental duplications. [γ] LINEs (green), LTRs (yellow), SINEs (purple), and Simple repeats (blue). [δ] Genes with colors denotes transcriptional orientation (the white represents “+” and the black represent “-“). [ϵ] Names of ANKRD26 homologs are marked in red. (PDF 557 KB)

Additional file 7: **A phylogenetic tree including 142 proteins of ANKRD26 homologs in mammals (132 proteins), in birds (6 proteins) and in reptilians (4 proteins).** Proteins of GI number were clustered in four groups of ancient ANKRD26-like (red), ANKRD18/ANKRD20 (green), ANKRD36/ANKRD62 (orange), and POTE (blue). The proteins of birds and those of reptilians are marked as triangles and circles, respectively. (PDF 158 KB)

Additional file 8: **Cattle chromosomes are used as reference chromosomes and are respectively compared to goat RH maps, pig (** ***S. scrofa*** **), horse (** ***E. caballus*** **), dog (** ***C. familiaris*** **), human (** ***H. sapiens*** **), macaque (** ***M. mulatta*** **), mouse (** ***M. musculus*** **), and rat (** ***R. norvegicus*** **) in the Evolution Highyway program.** We detected the genomic location of 115 members of ANKRD26 family in nine mammalian genome (excluding unmapped scaffolds), of which 109 locate in breakpoints (red cross) and 6 locate in homologous synteny (blue cross). Most of the members expanded specifically in species and lineage specific evolutionary breakpoints or in centromeres. The duplication of ANKRD26 gene follows the patterns of convergent breakpoint reuse through chromosome evolution. (TIFF 2 MB)

Additional file 9: **Fast identification of conserved domains in protein sequences of human ANKRD26 homologs was performed using NCBI Conserved Domain Database.** Ankyrin domains are near to N-terminal and structural maintenance of chromosomes protein (SMC) domains are near to C-terminal. (PDF 411 KB)

## Abbreviations

BTA: *Bos taurus*
CHI: *Capra hircus*
HSB: Homologous synteny block
LKH: Lin-Kernighan heuristic
LOD scores: Log odds score method
MCMC: Markov Chain Monte Carlo
NAHR: Nonallelic homologous recombination
RH: Radiation hybrid
SD: Segmental duplication
SNP: Single nucleotide polymorphism
TSP: Traveling Salesman problem.
