# Supplementary material for: An update of the goat genome assembly using dense radiation hybrid maps allows detailed analysis of evolutionary rearrangements in Bovidae
Source: BMC Genomics. 2014 Jul 23;15(1):625. doi: 10.1186/1471-2164-15-625 (PMC4141111; doi:10.1186/1471-2164-15-625)
Supplement: Supplementary file 3 — Additional file 3: Detailed comparison of RH maps with the CHIR_1.0 and with the CHIR_1.1. This file contains comprehensive pictures comparing (i) CHIR_1.0 and the RH maps, as well as (ii) the goat genome sequence CHIR_1.1 and the RH maps. (PDF 348 KB) [file 12864_2013_6362_MOESM3_ESM.pdf]

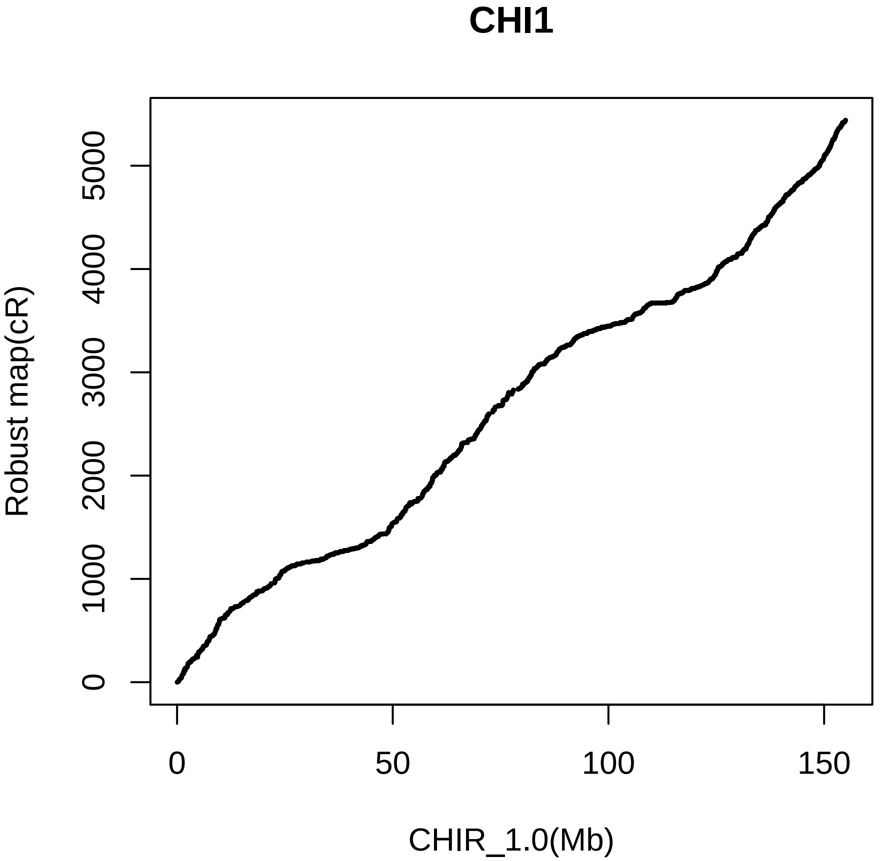
CHI1
Robust map(cR)
0
1000
2000
3000
4000
5000
0
50
100
150
CHIR_1.0(Mb)

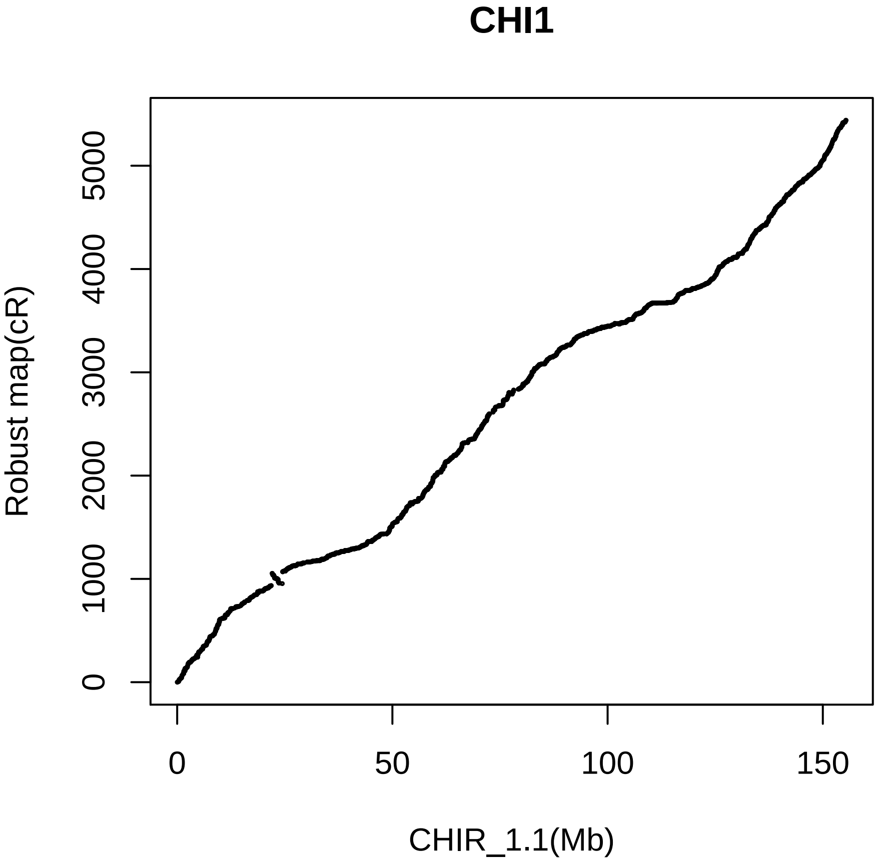
CHI1
Robust map(cR)
0
1000
2000
3000
4000
5000
0
50
100
150
CHIR_1.1(Mb)

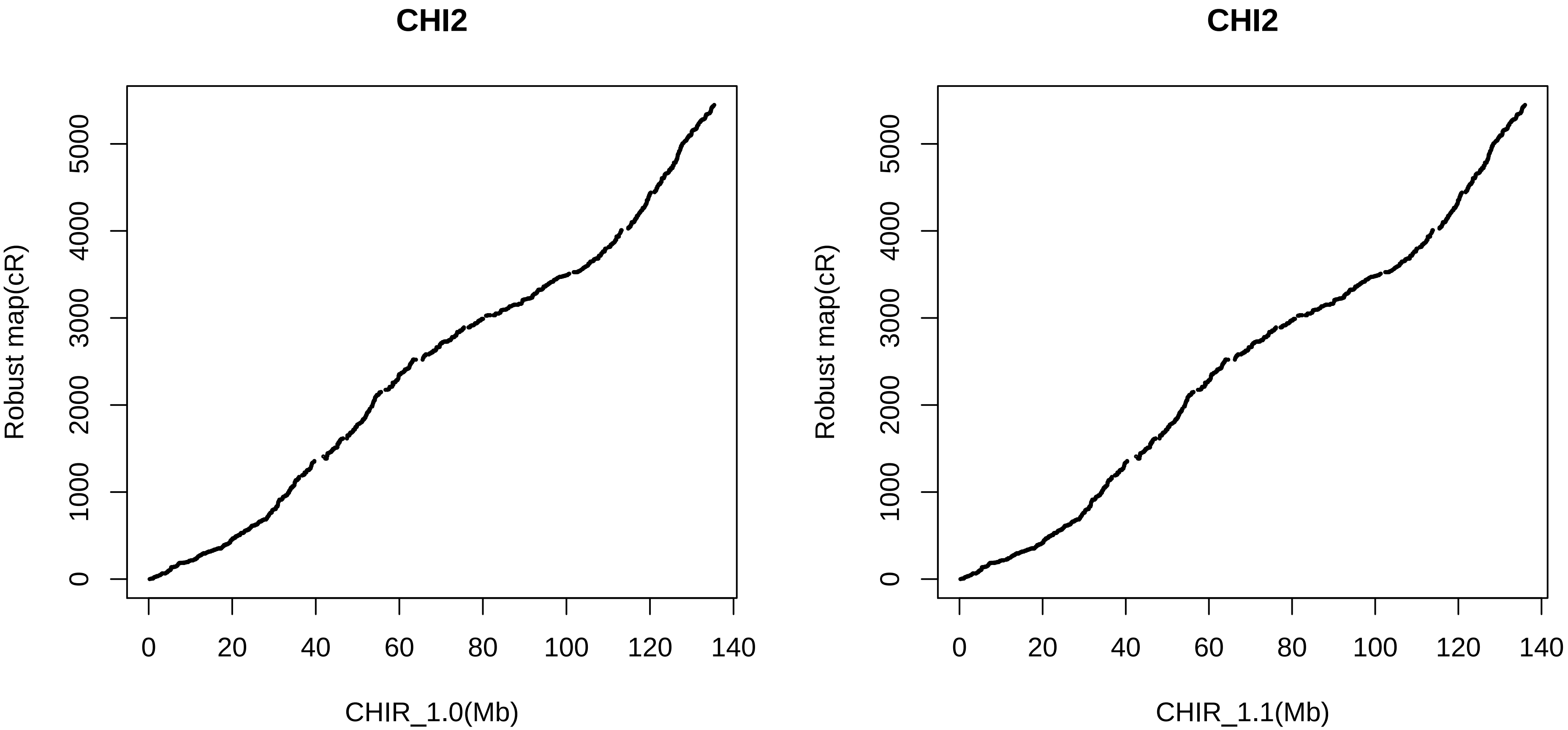
CHI2
Robust map(cR)
0
1000
2000
3000
4000
5000
0
20
40
60
80
100
120
140
CHIR_1.0(Mb)
CHI2
Robust map(cR)
0
1000
2000
3000
4000
5000
0
20
40
60
80
100
120
140
CHIR_1.1(Mb)

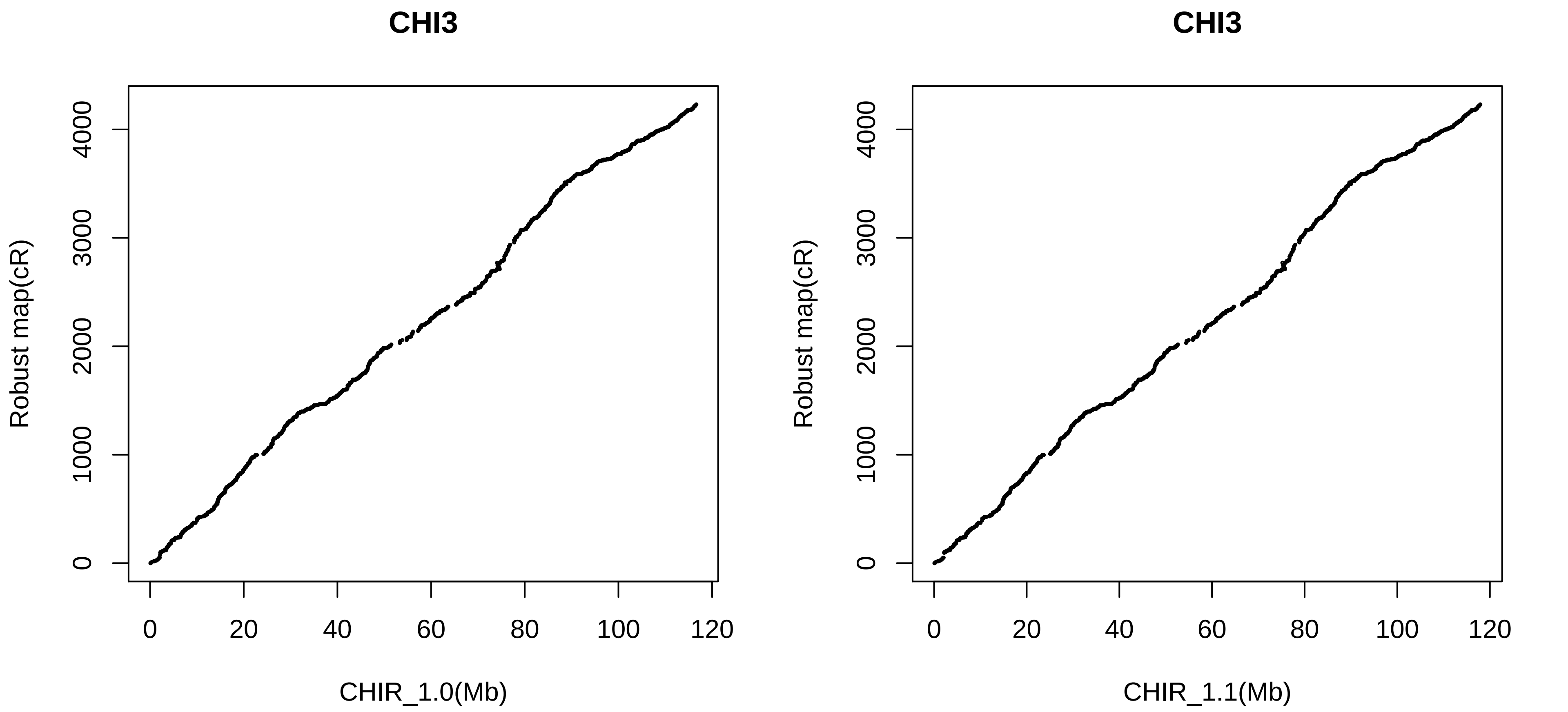
CHI3
Robust map(cR)
0
1000
2000
3000
4000
0
20
40
60
80
100
120
CHIR_1.0(Mb)
CHI3
Robust map(cR)
0
1000
2000
3000
4000
0
20
40
60
80
100
120
CHIR_1.1(Mb)

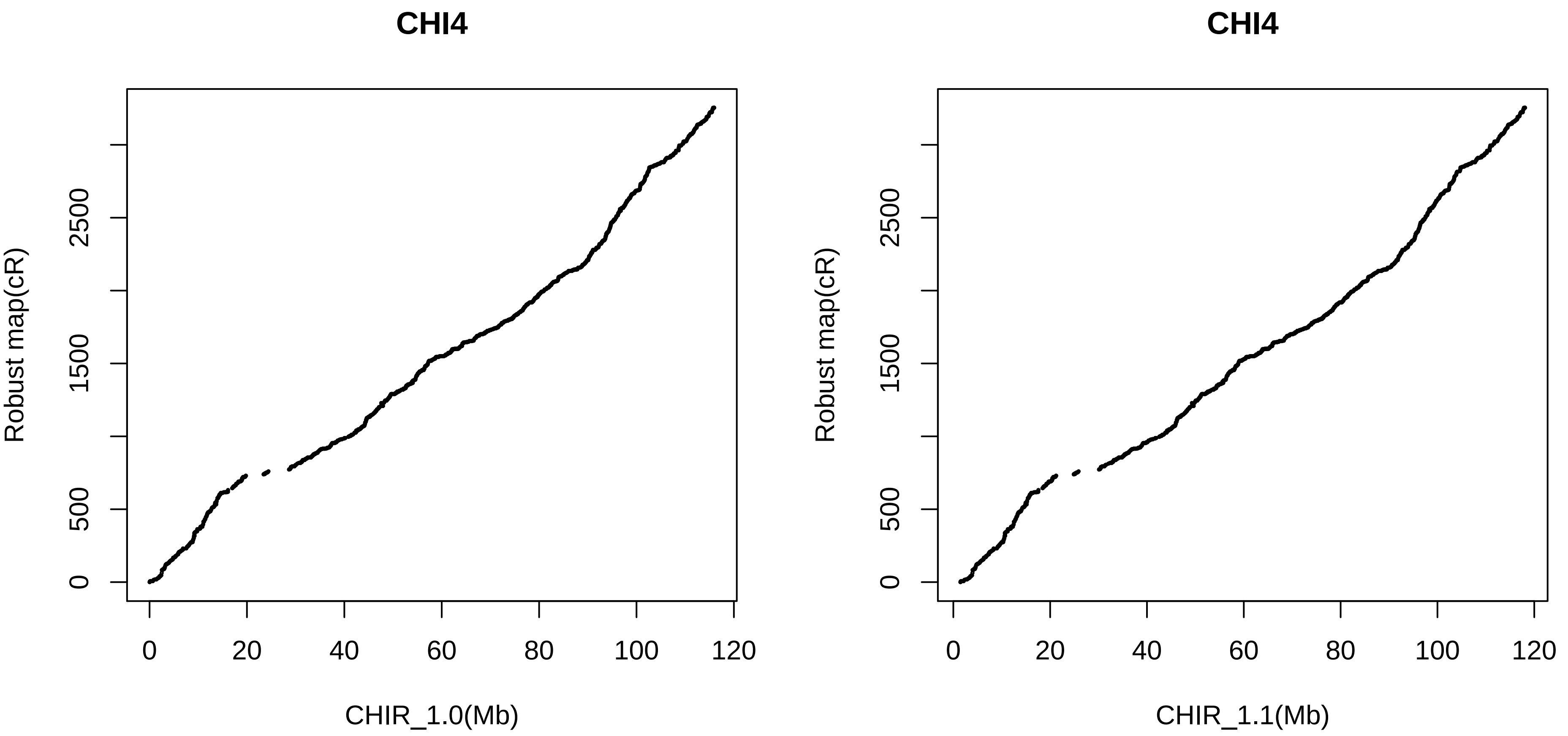

CHI4
Robust map(cR)
0
500
1500
2500
0
20
40
60
80
100
120
CHIR_1.0(Mb)
CHI4
Robust map(cR)
0
500
1500
2500
0
20
40
60
80
100
120
CHIR_1.1(Mb)

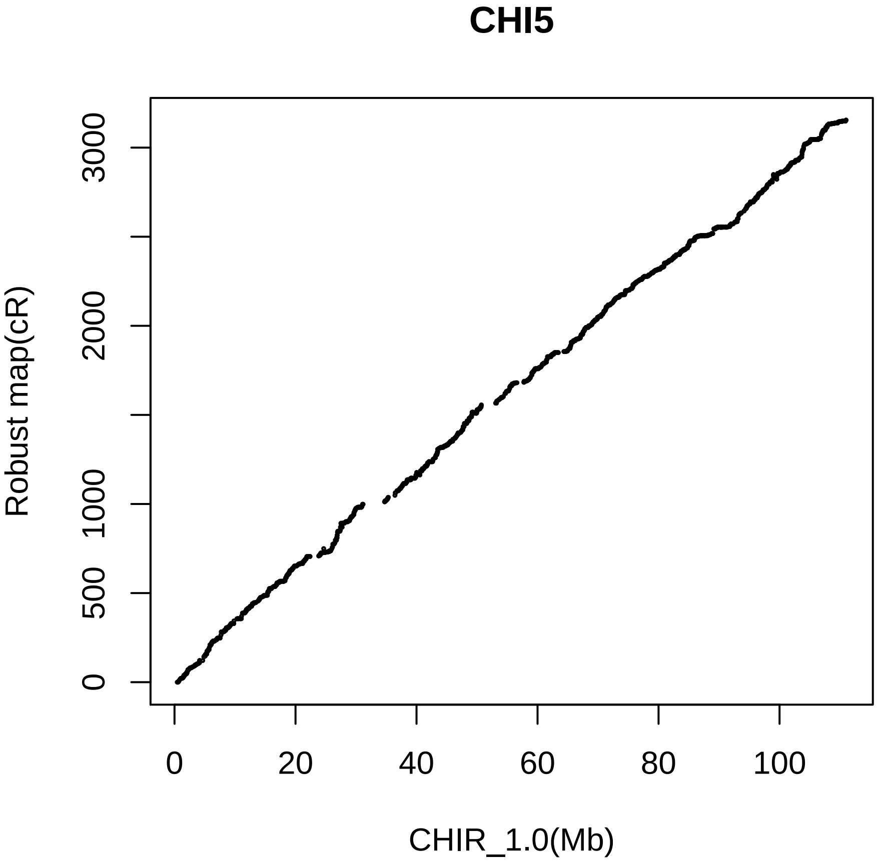
CHI5
Robust map(cR)
0
500
1000
2000
3000
0
20
40
60
80
100
CHIR_1.0(Mb)

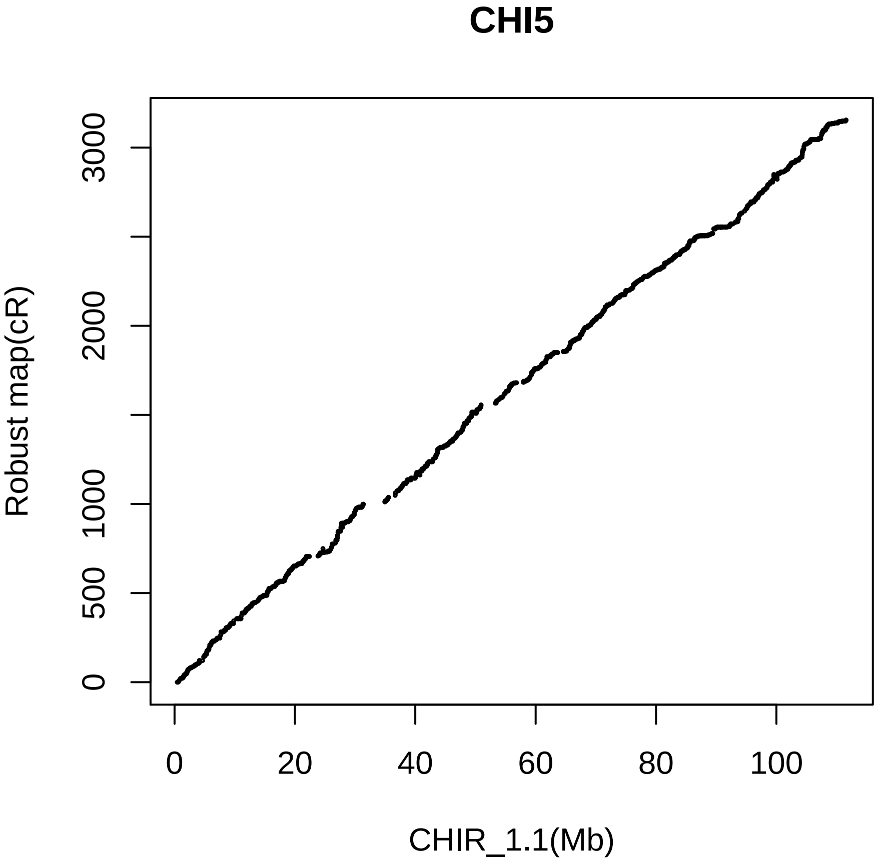
CHI5
Robust map(cR)
0
500
1000
2000
3000
0
20
40
60
80
100
CHIR_1.1(Mb)

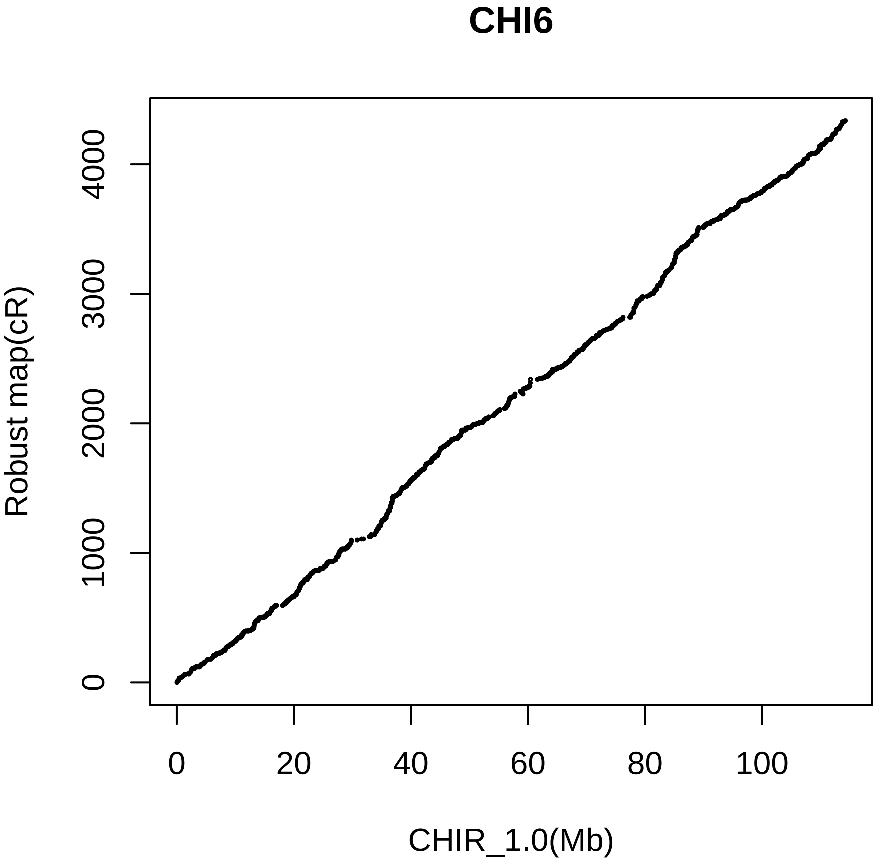
CHI6
Robust map(cR)
0
1000
2000
3000
4000
0
20
40
60
80
100
CHIR_1.0(Mb)

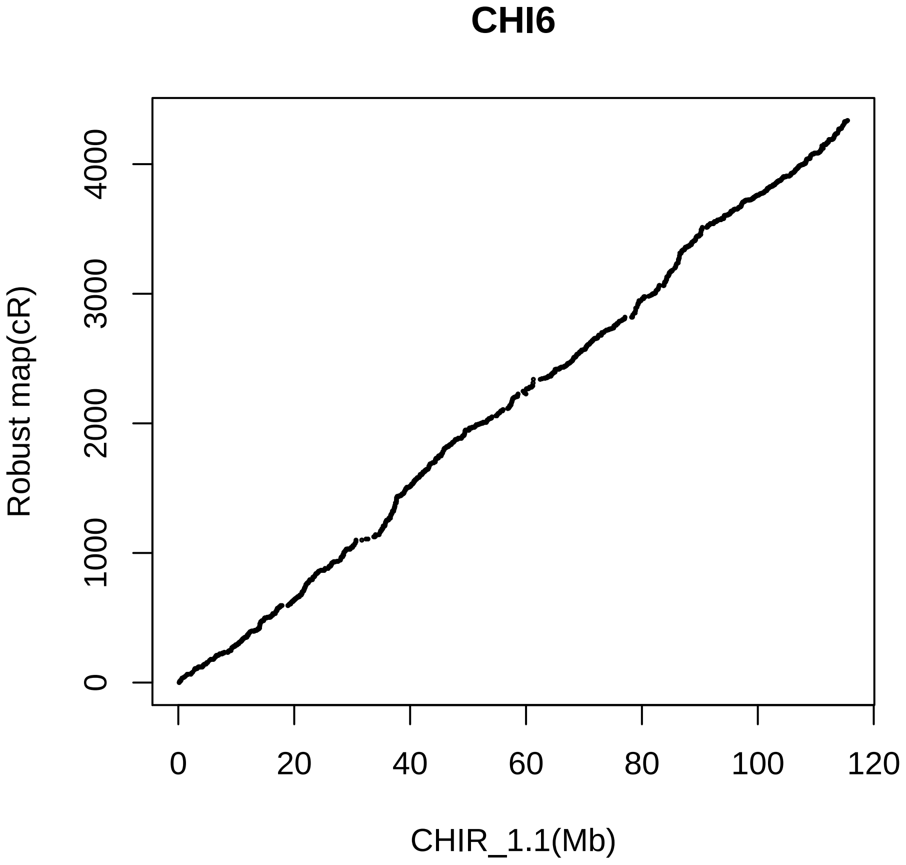
CHI6
Robust map(cR)
0
1000
2000
3000
4000
0
20
40
60
80
100
120
CHIR_1.1(Mb)

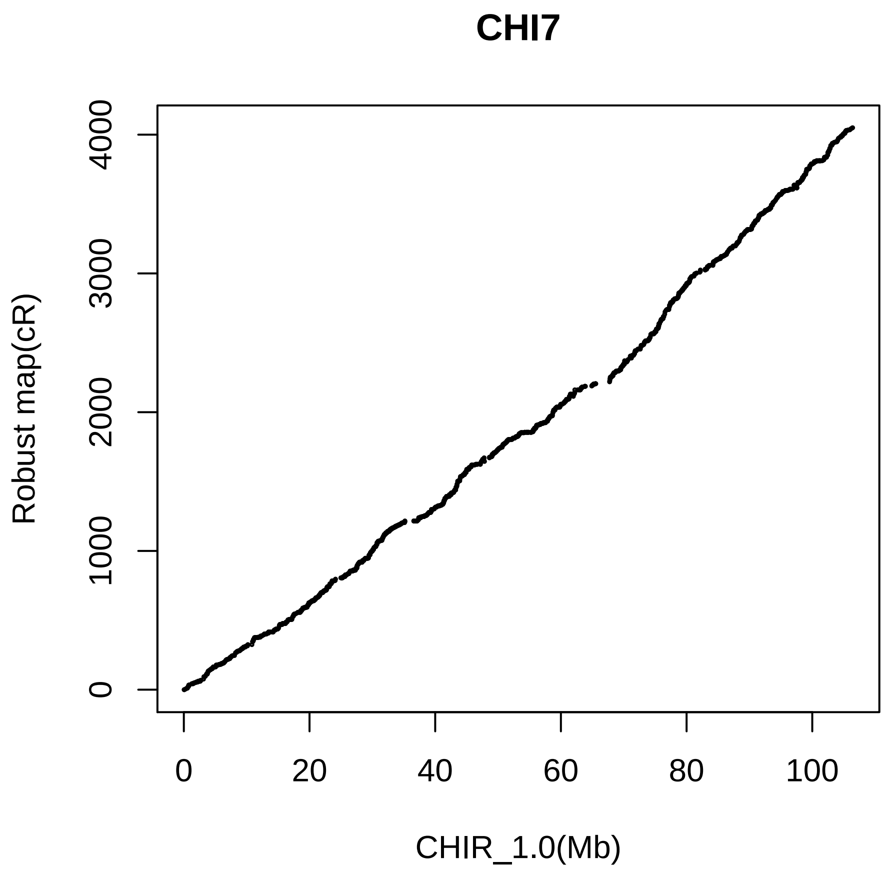
CHI7
Robust map(cR)
CHIR_1.0(Mb)
0
1000
2000
3000
4000
0
20
40
60
80
100

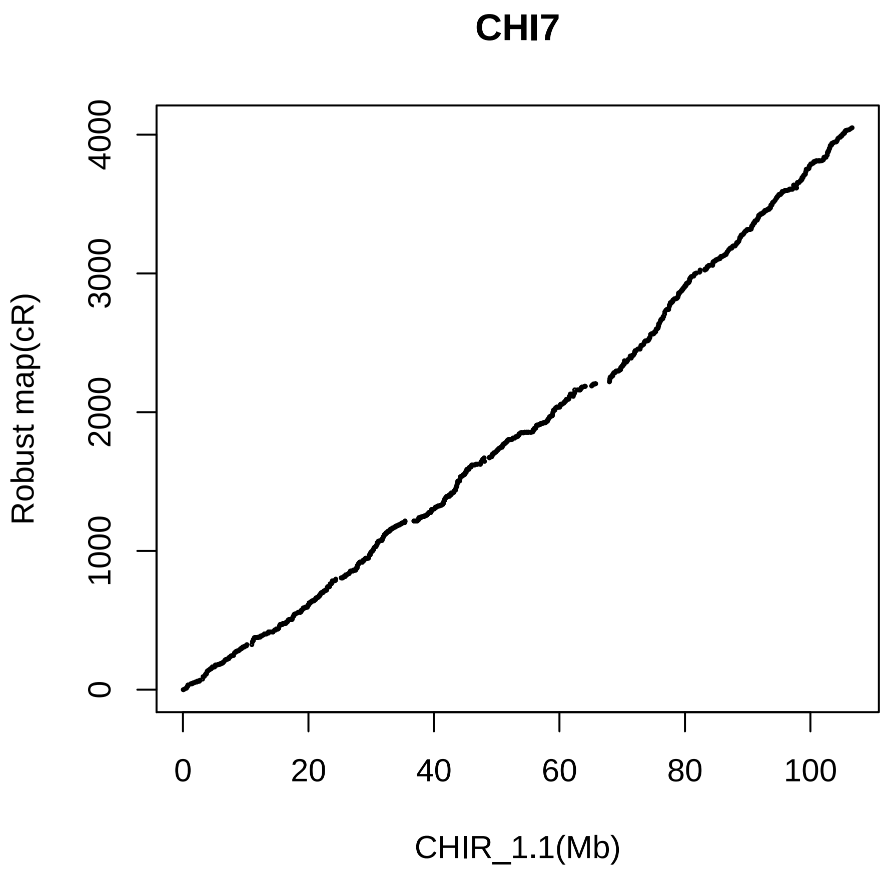
CHI7
Robust map(cR)
CHIR_1.1(Mb)
0
1000
2000
3000
4000
0
20
40
60
80
100

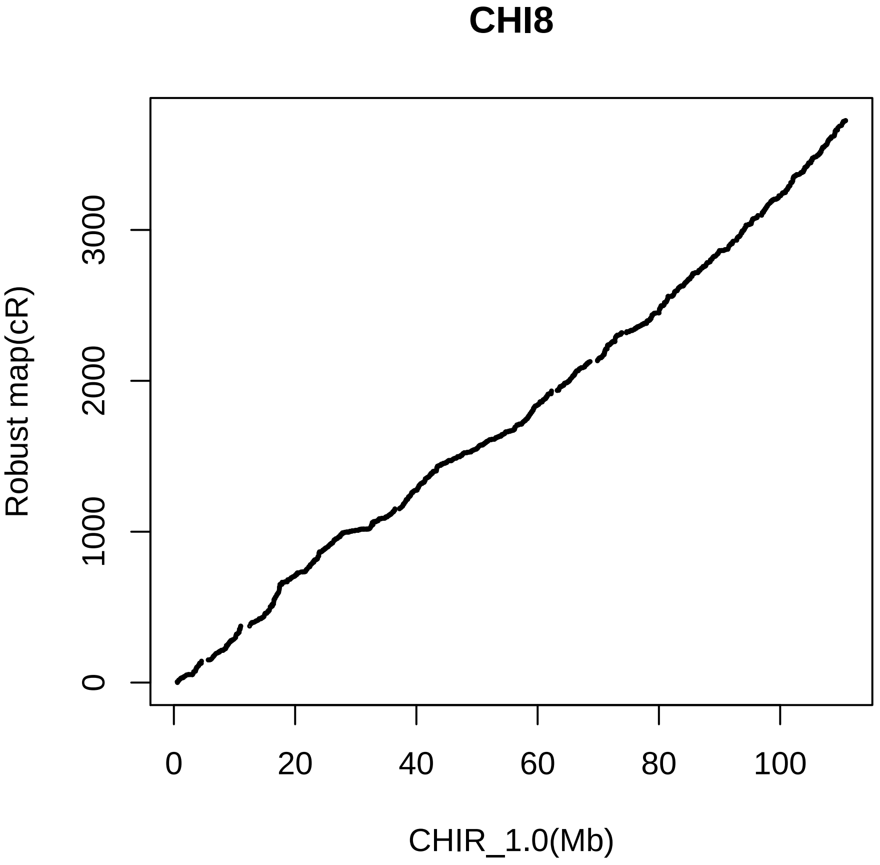

CHI8
Robust map(cR)
0
1000
2000
3000
0
20
40
60
80
100
CHIR_1.0(Mb)

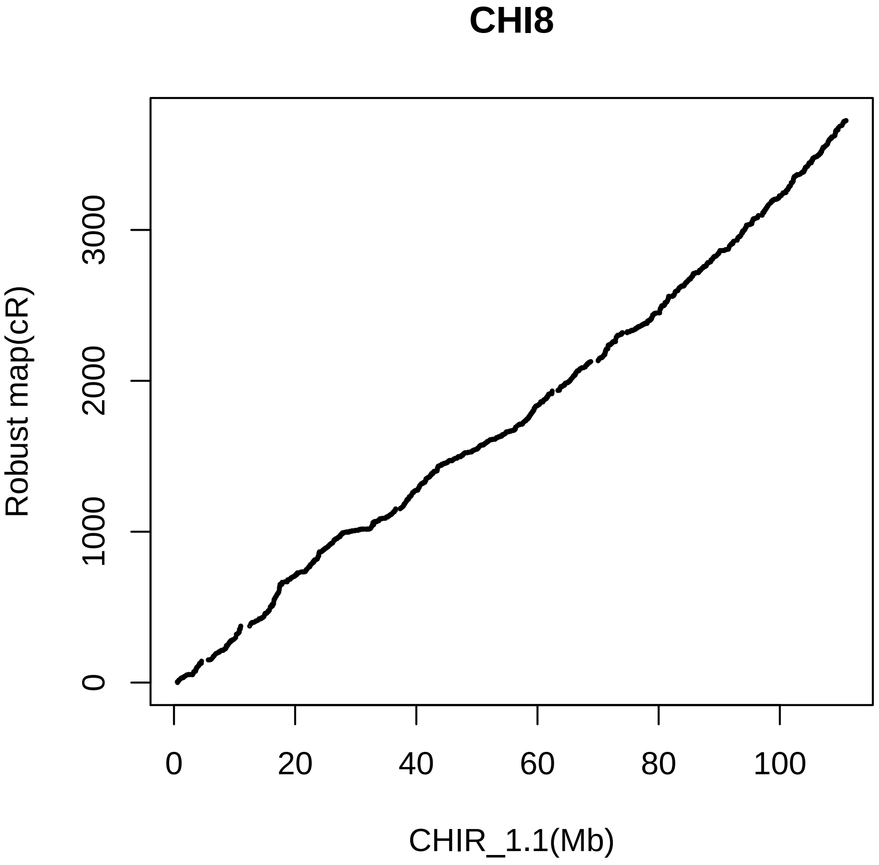

CHI8
Robust map(cR)
0
1000
2000
3000
0
20
40
60
80
100
CHIR_1.1(Mb)

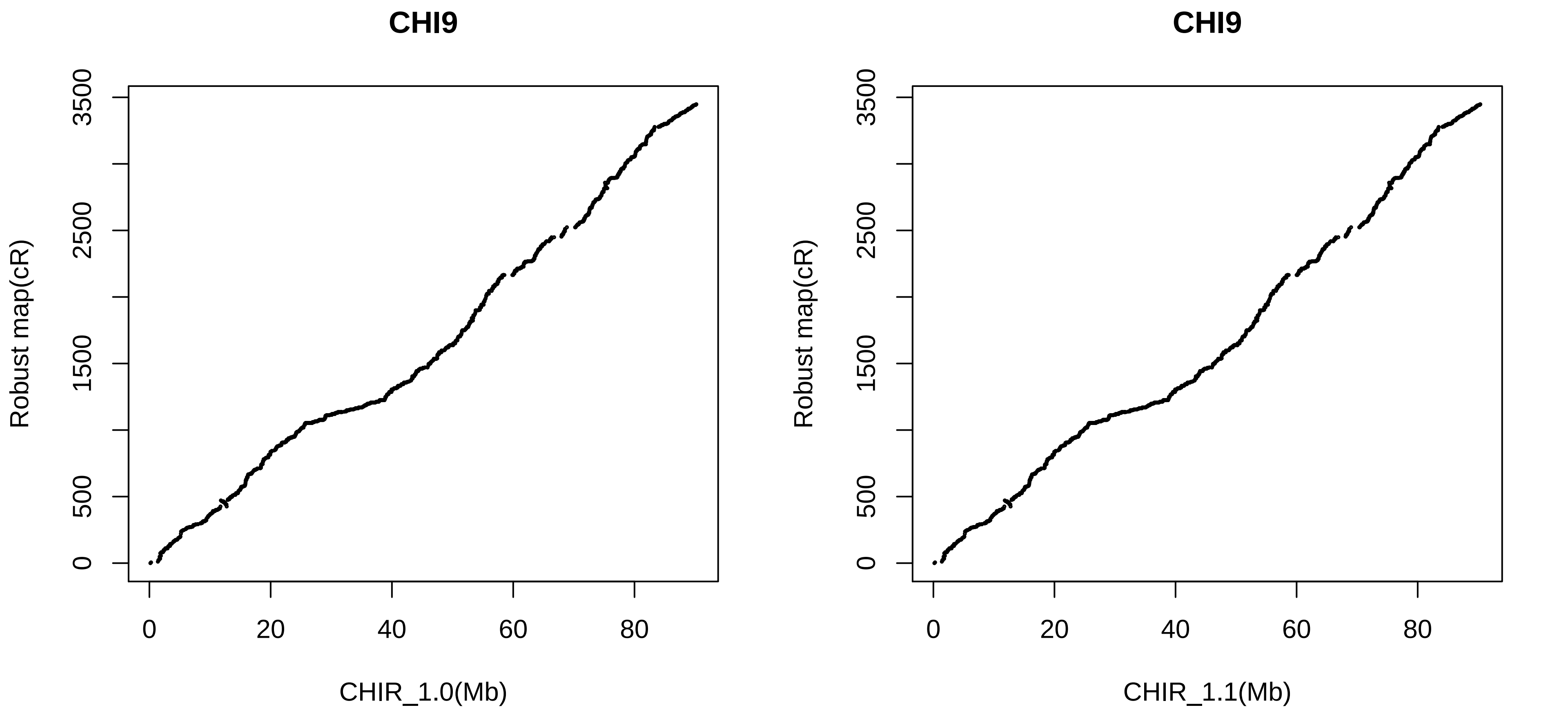
CHI9
Robust map(cR)
0
500
1500
2500
3500
0
20
40
60
80
CHIR_1.0(Mb)
CHI9
Robust map(cR)
0
500
1500
2500
3500
0
20
40
60
80
CHIR_1.1(Mb)

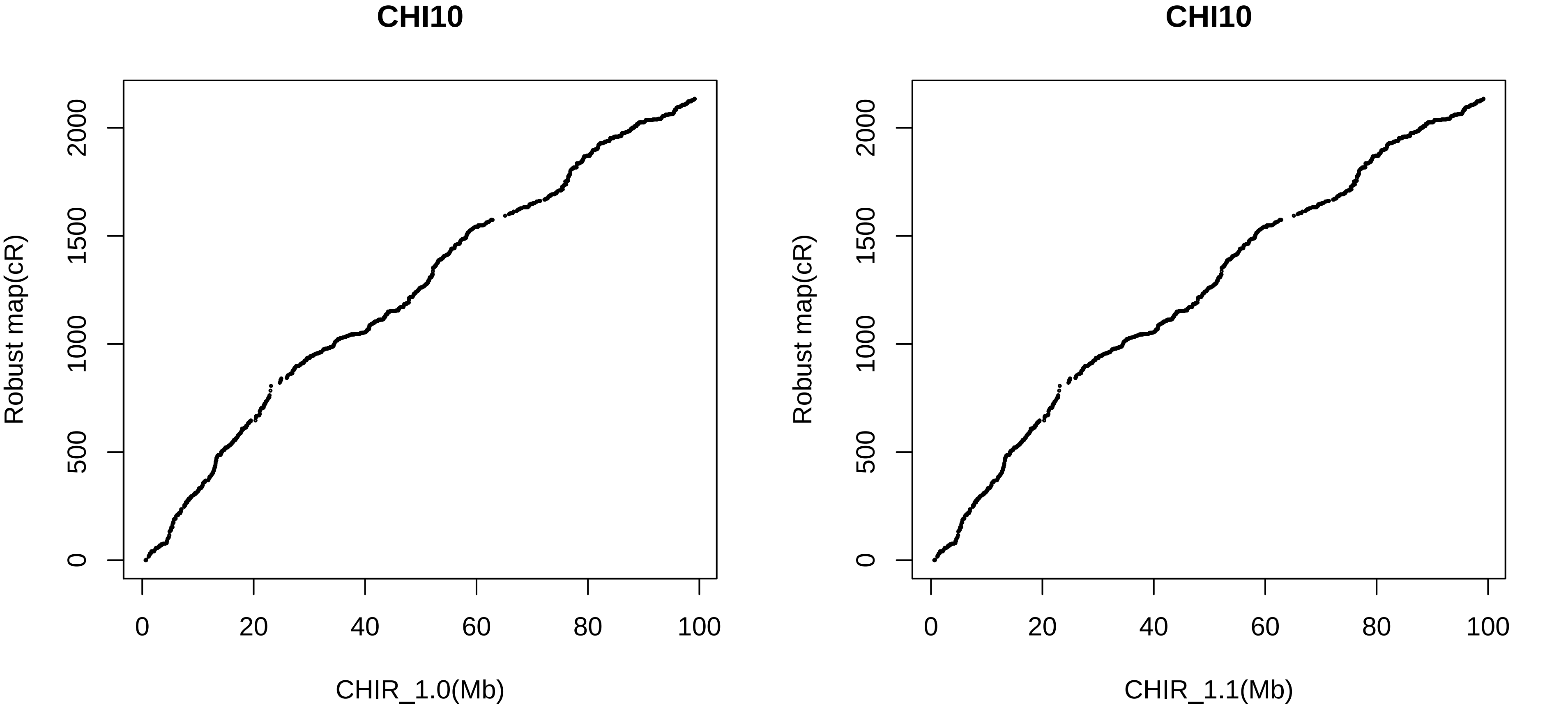
CHI10
Robust map(cR)
CHIR_1.0(Mb)
CHI10
Robust map(cR)
CHIR_1.1(Mb)

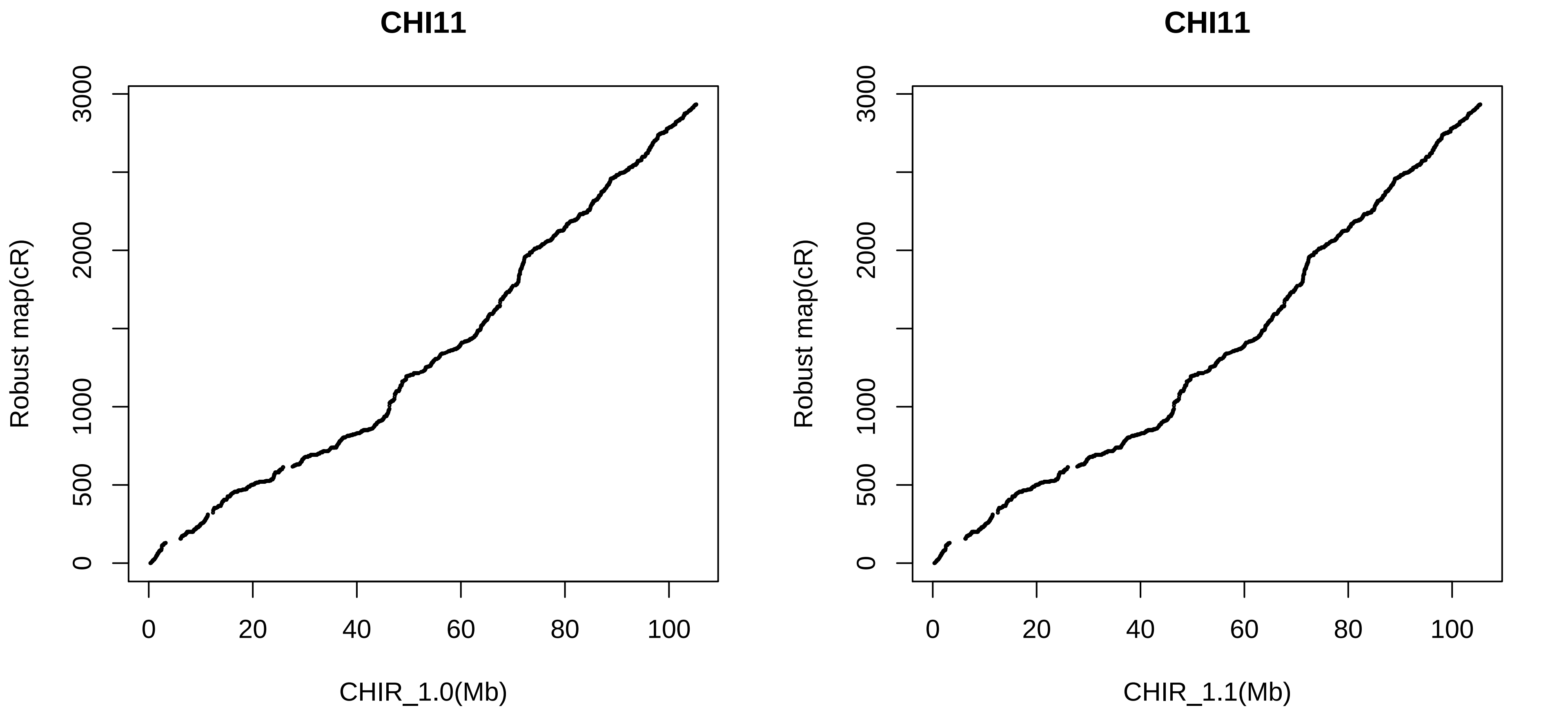

CHI11
Robust map(cR)
0
500
1000
2000
3000
0
20
40
60
80
100
CHIR_1.0(Mb)
CHI11
Robust map(cR)
0
500
1000
2000
3000
0
20
40
60
80
100
CHIR_1.1(Mb)

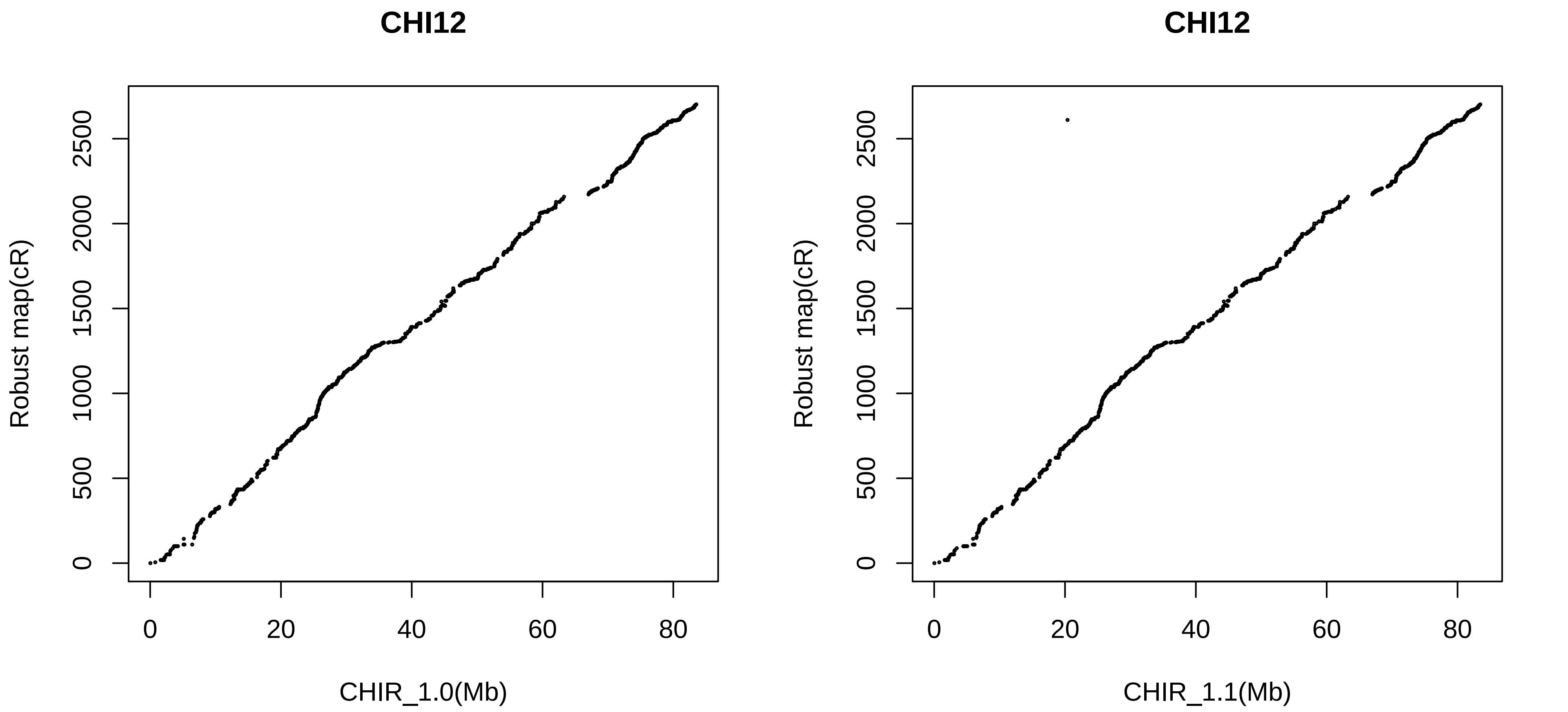

CHI12
Robust map(cR)
0
500
1000
1500
2000
2500
0
20
40
60
80
CHIR_1.0(Mb)
CHI12
Robust map(cR)
0
500
1000
1500
2000
2500
0
20
40
60
80
CHIR_1.1(Mb)

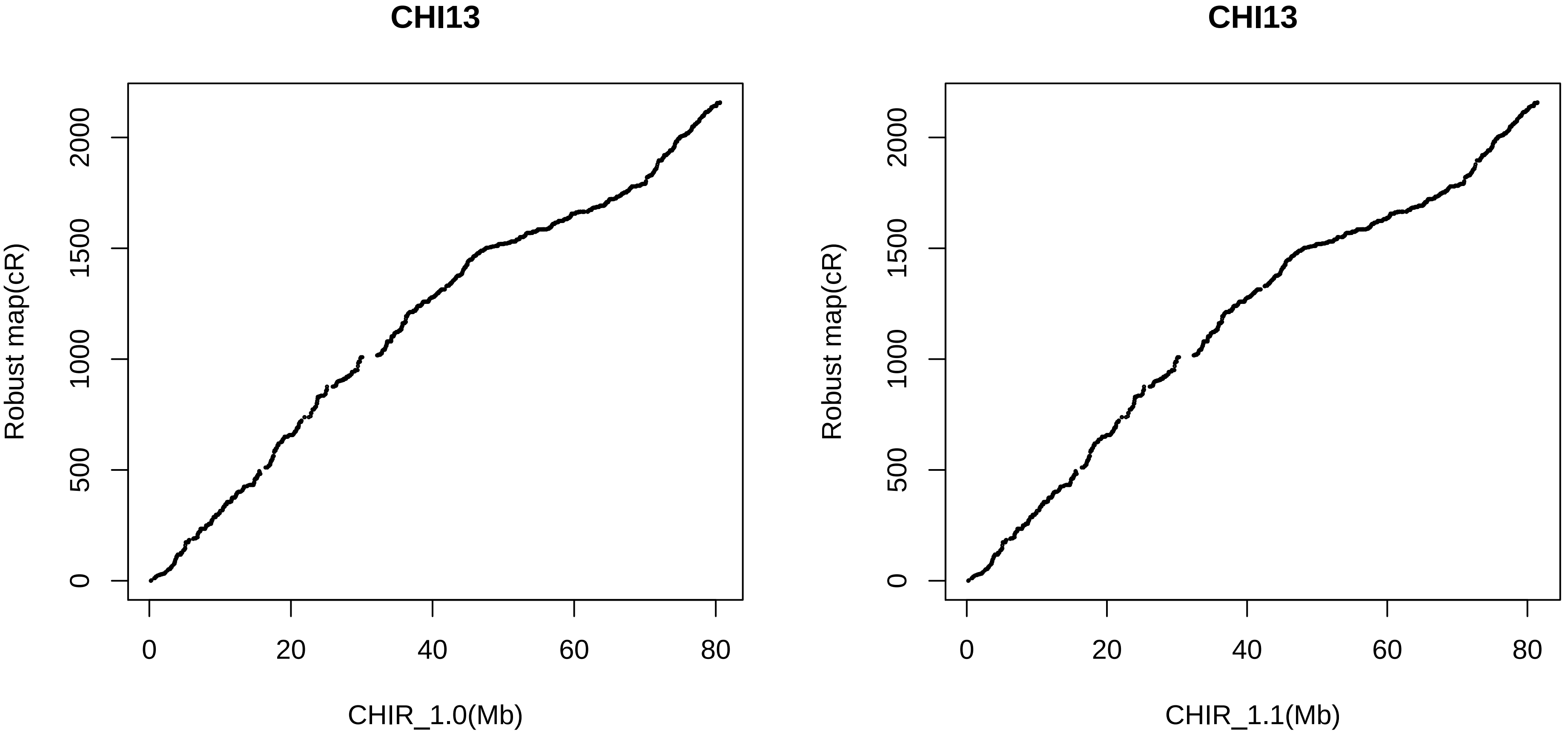

CHI13
Robust map(cR)
0
500
1000
1500
2000
0
20
40
60
80
CHIR_1.0(Mb)
CHI13
Robust map(cR)
0
500
1000
1500
2000
0
20
40
60
80
CHIR_1.1(Mb)

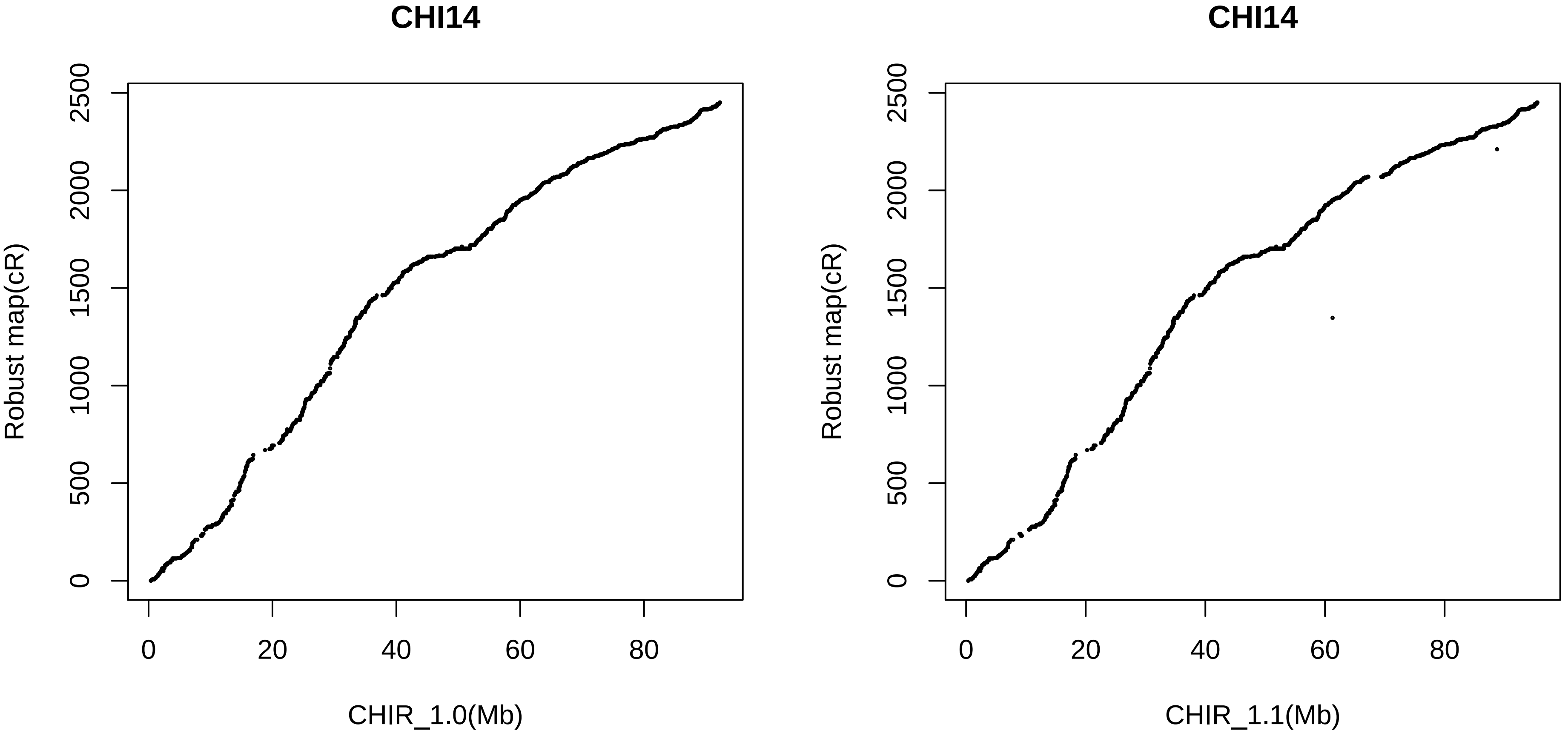
CHI14
Robust map(cR)
0
500
1000
1500
2000
2500
0
20
40
60
80
CHIR_1.0(Mb)
CHI14
Robust map(cR)
0
500
1000
1500
2000
2500
0
20
40
60
80
CHIR_1.1(Mb)

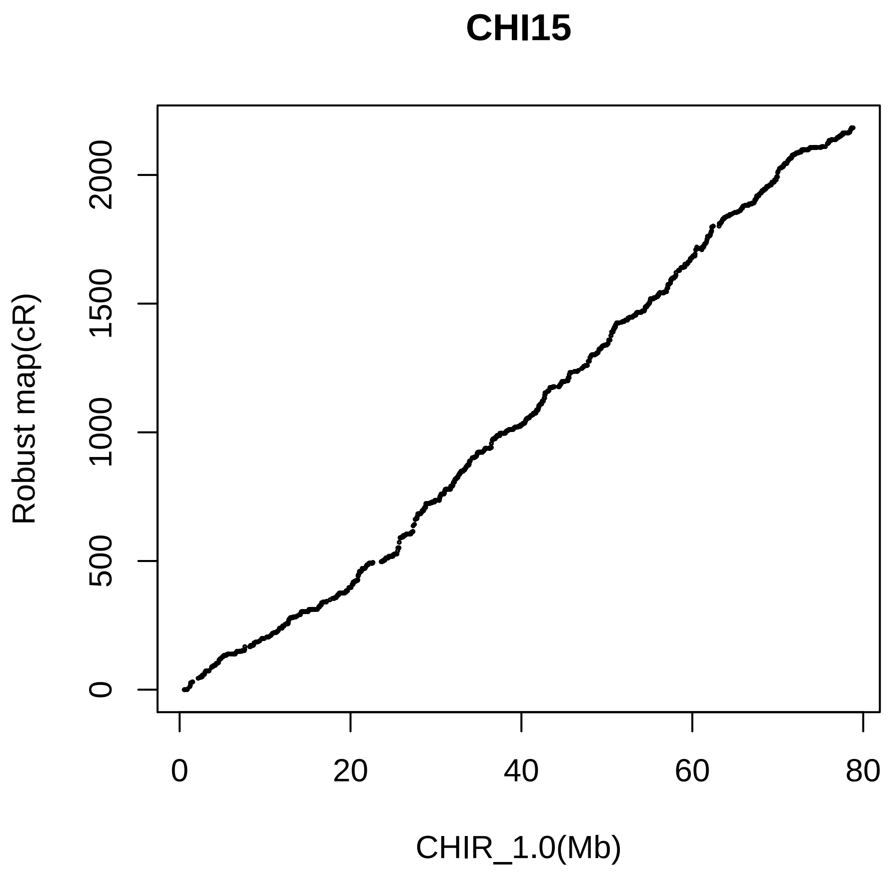
CHI15
Robust map(cR)
0
500
1000
1500
2000
0
20
40
60
80
CHIR_1.0(Mb)

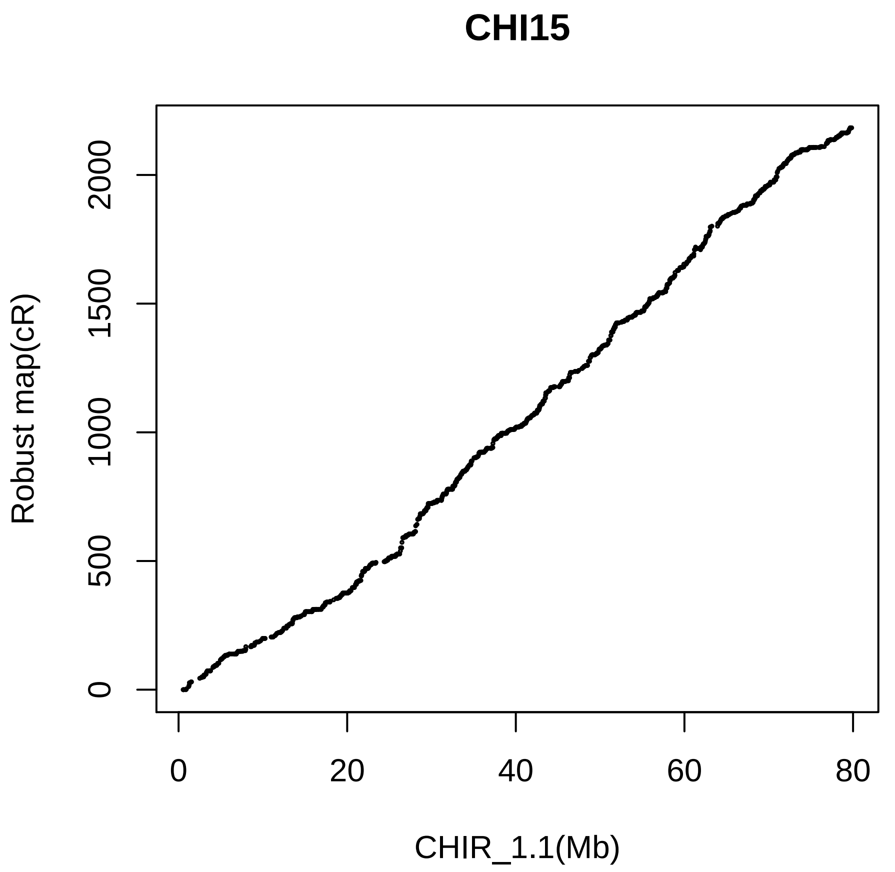
CHI15
Robust map(cR)
0
500
1000
1500
2000
0
20
40
60
80
CHIR_1.1(Mb)

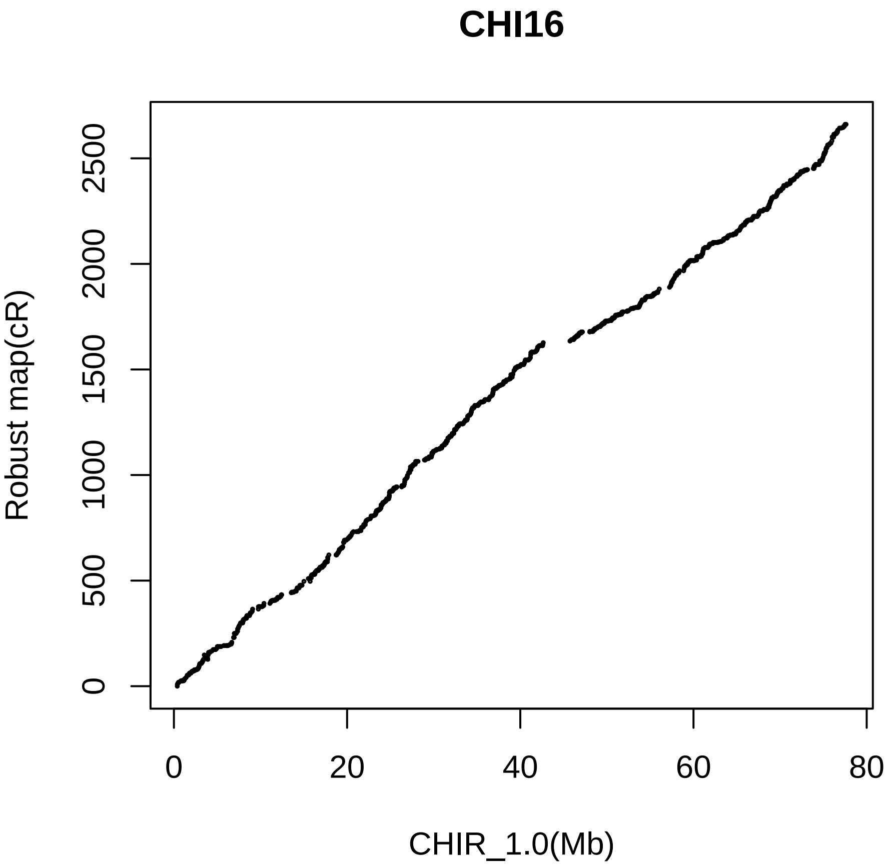
CHI16
Robust map(cR)
CHIR_1.0(Mb)
0
500
1000
1500
2000
2500
0
20
40
60
80

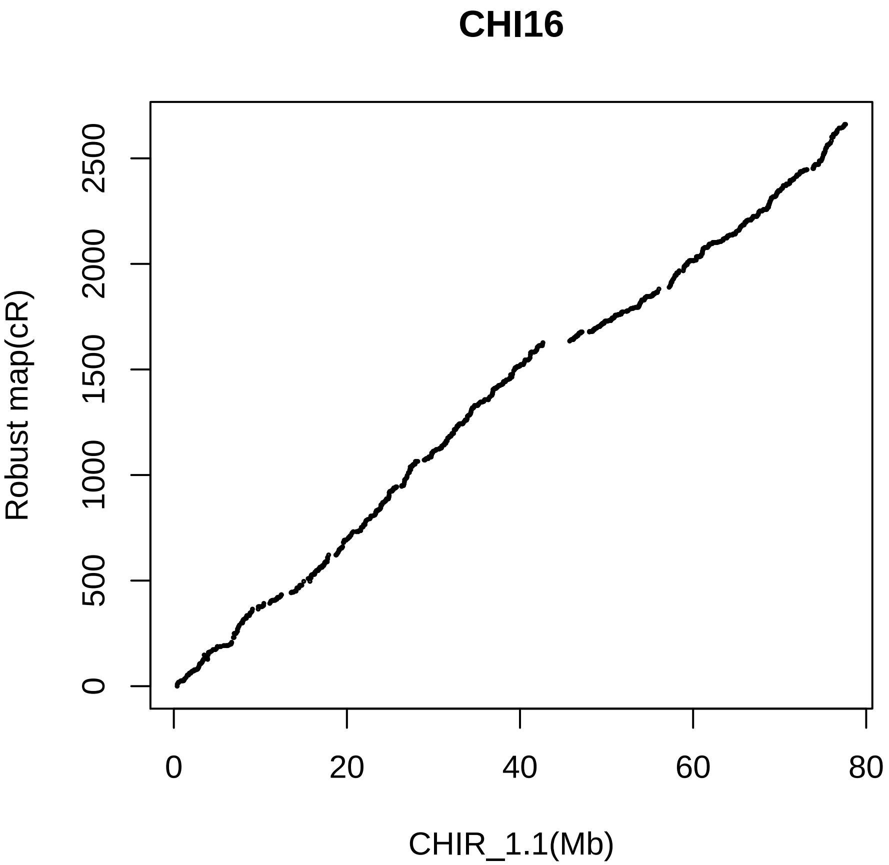
CHI16
Robust map(cR)
CHIR_1.1(Mb)
0
500
1000
1500
2000
2500
0
20
40
60
80

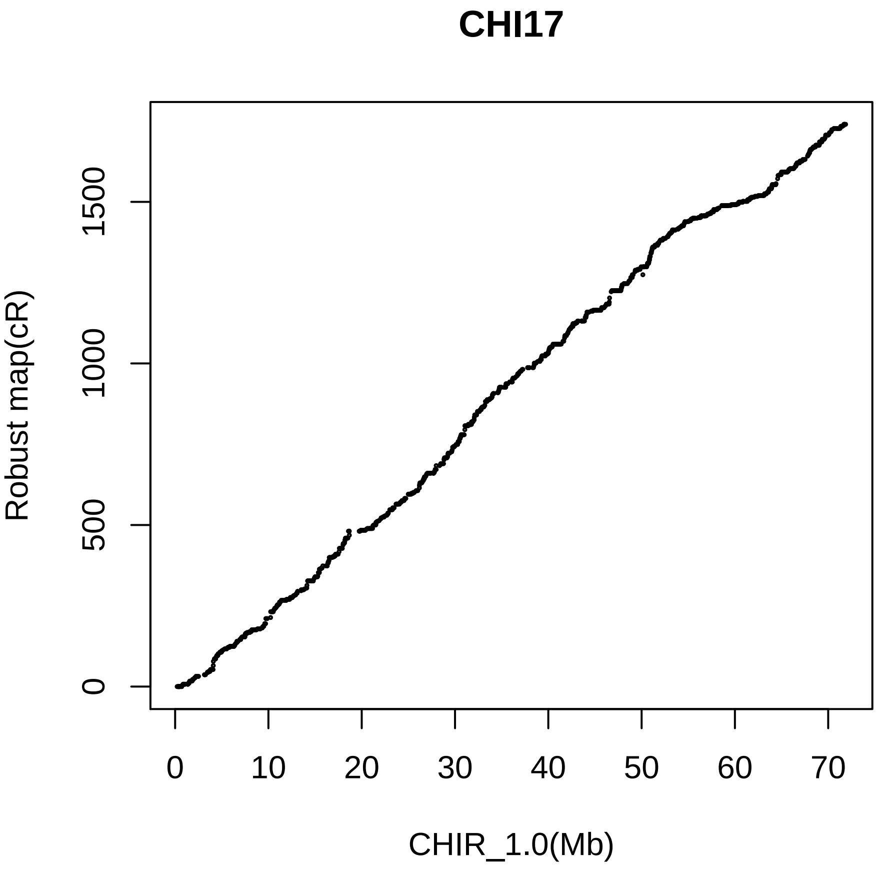
CHI17
Robust map(cR)
0
500
1000
1500
0
10
20
30
40
50
60
70
CHIR_1.0(Mb)

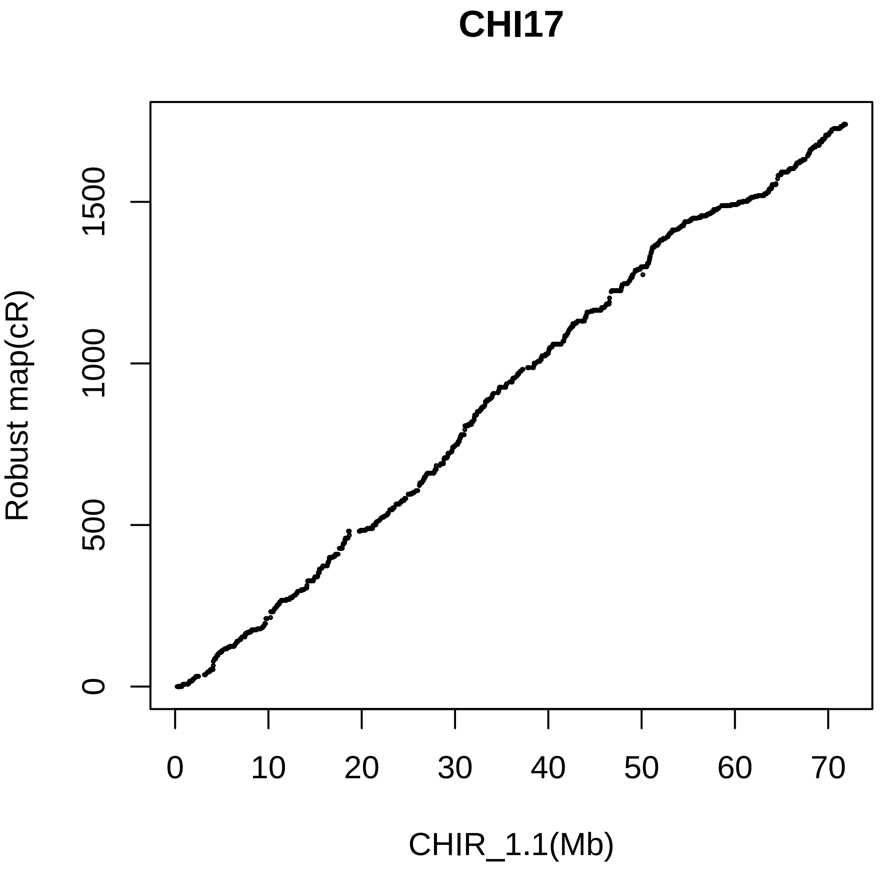
CHI17
Robust map(cR)
0
500
1000
1500
0
10
20
30
40
50
60
70
CHIR_1.1(Mb)

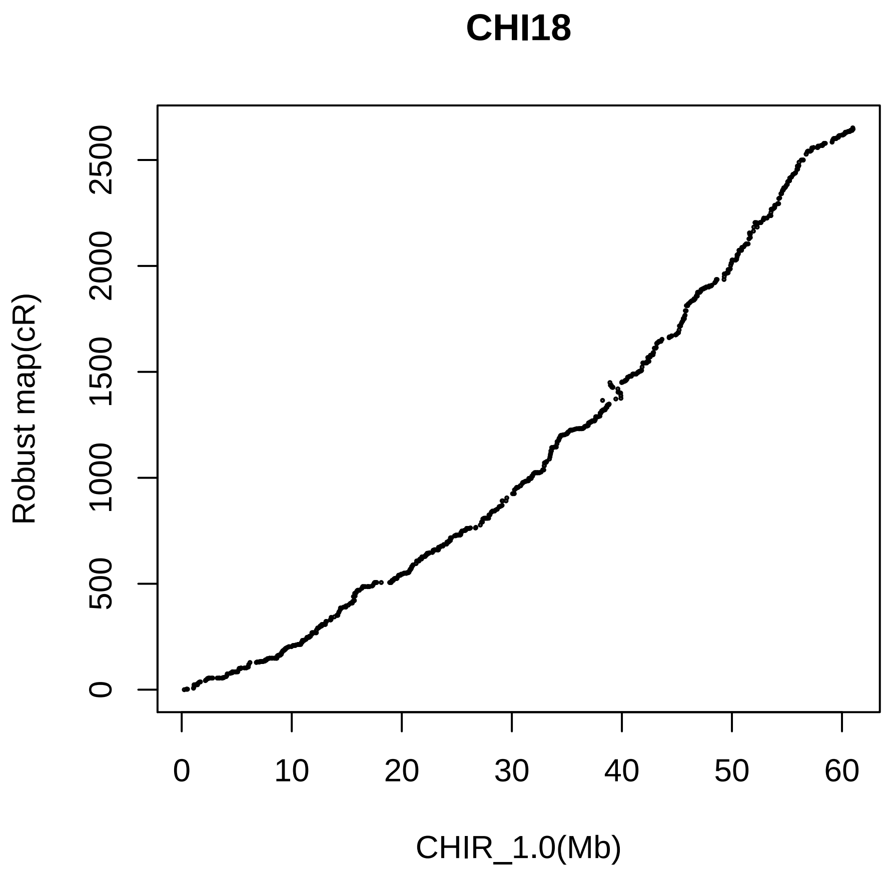

CHI18
Robust map(cR)
0
500
1000
1500
2000
2500
0
10
20
30
40
50
60
CHIR_1.0(Mb)

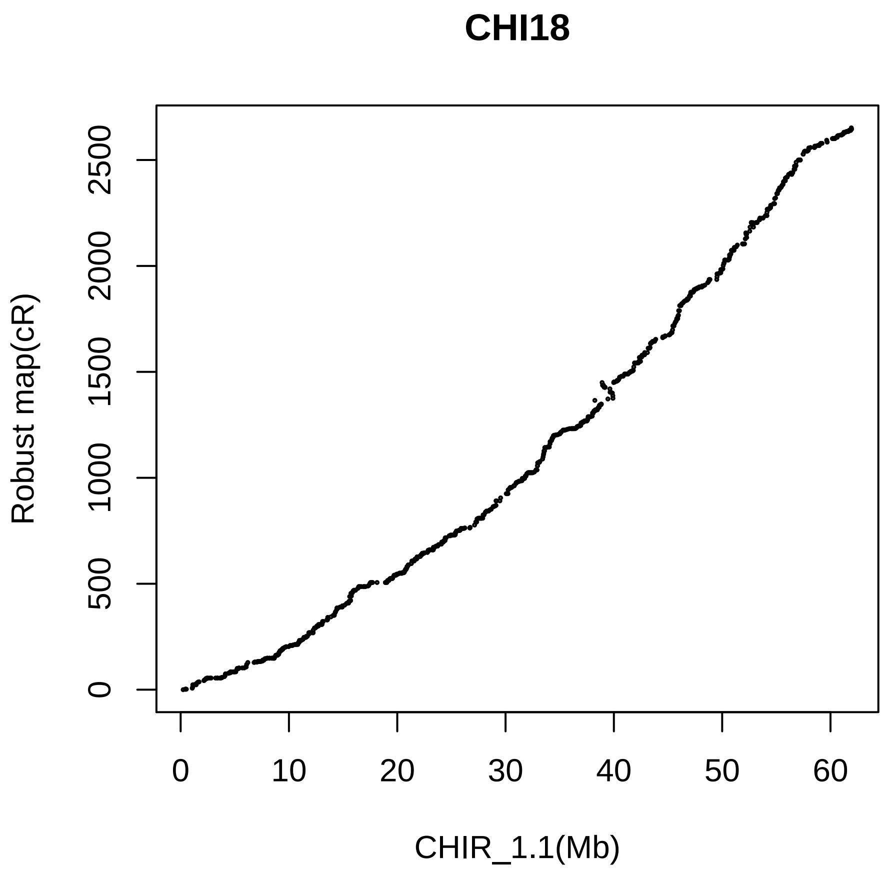

CHI18
Robust map(cR)
0
500
1000
1500
2000
2500
0
10
20
30
40
50
60
CHIR_1.1(Mb)

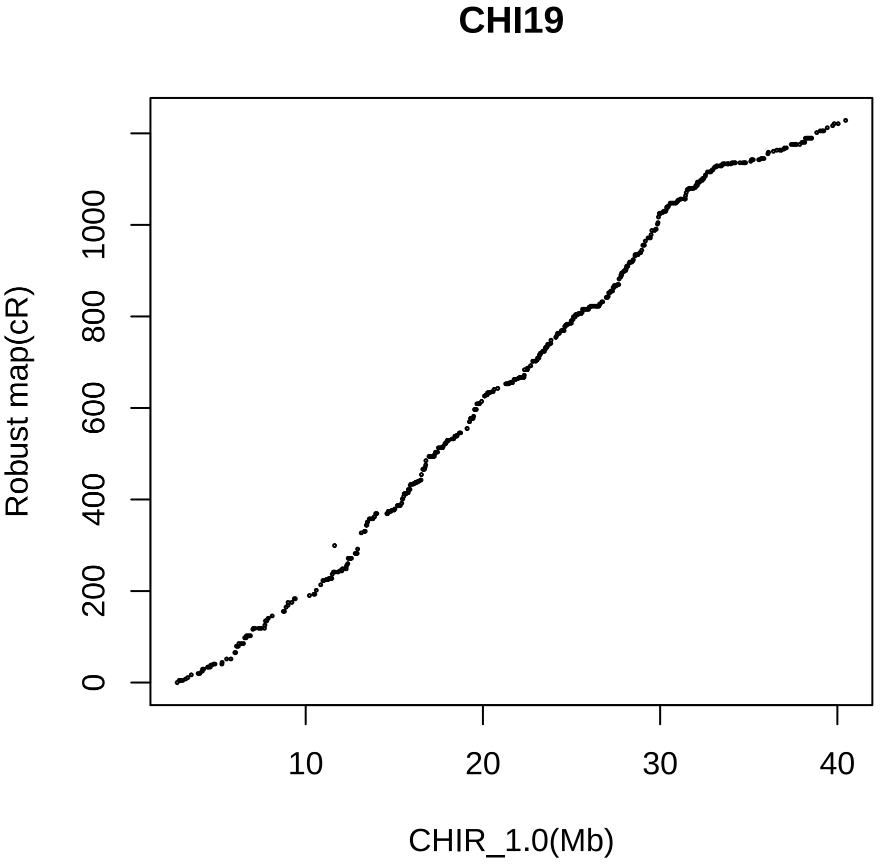
CHI19
Robust map(cR)
0
200
400
600
800
1000
10
20
30
40
CHIR_1.0(Mb)

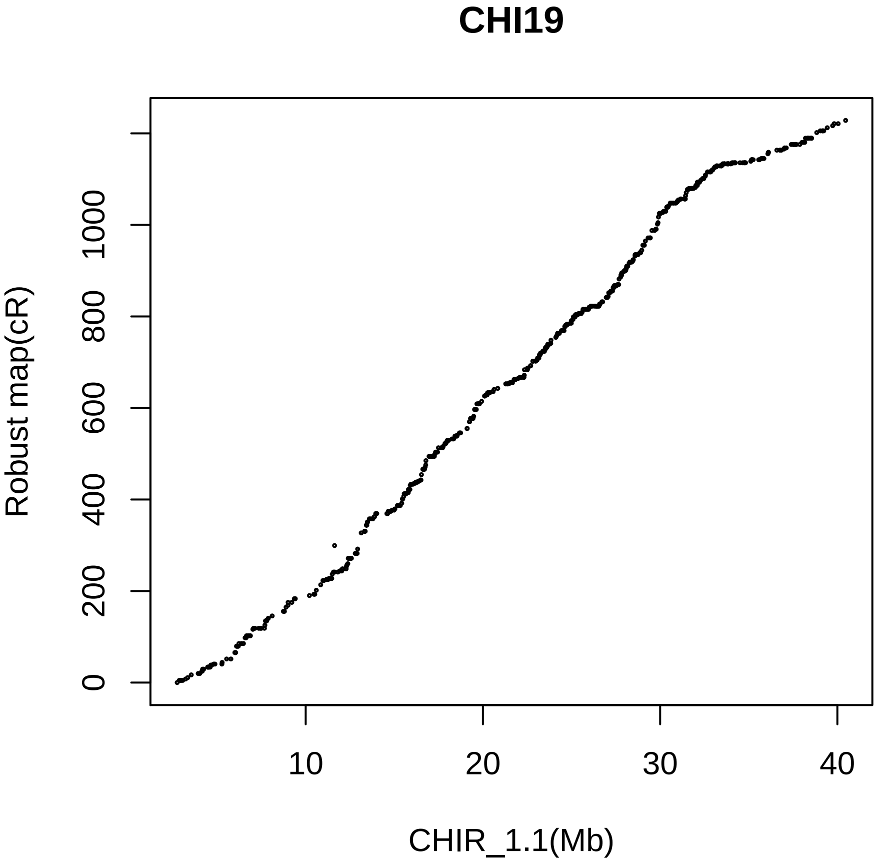
CHI19
Robust map(cR)
0
200
400
600
800
1000
10
20
30
40
CHIR_1.1(Mb)

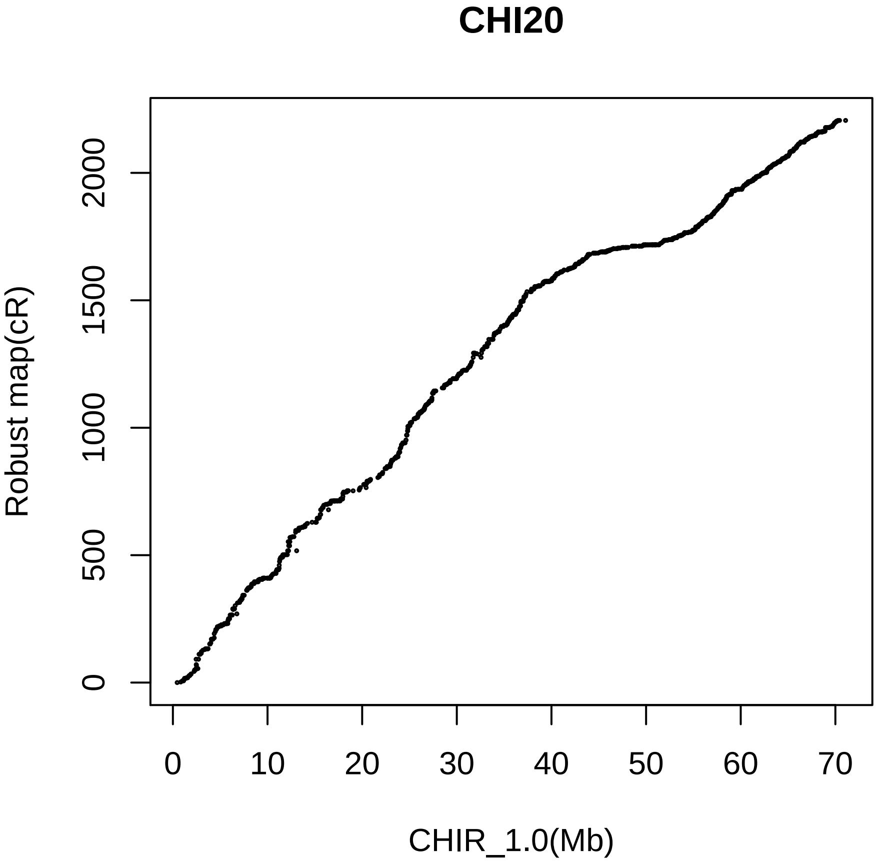

CHI20
Robust map(cR)
0
500
1000
1500
2000
0
10
20
30
40
50
60
70
CHIR_1.0(Mb)

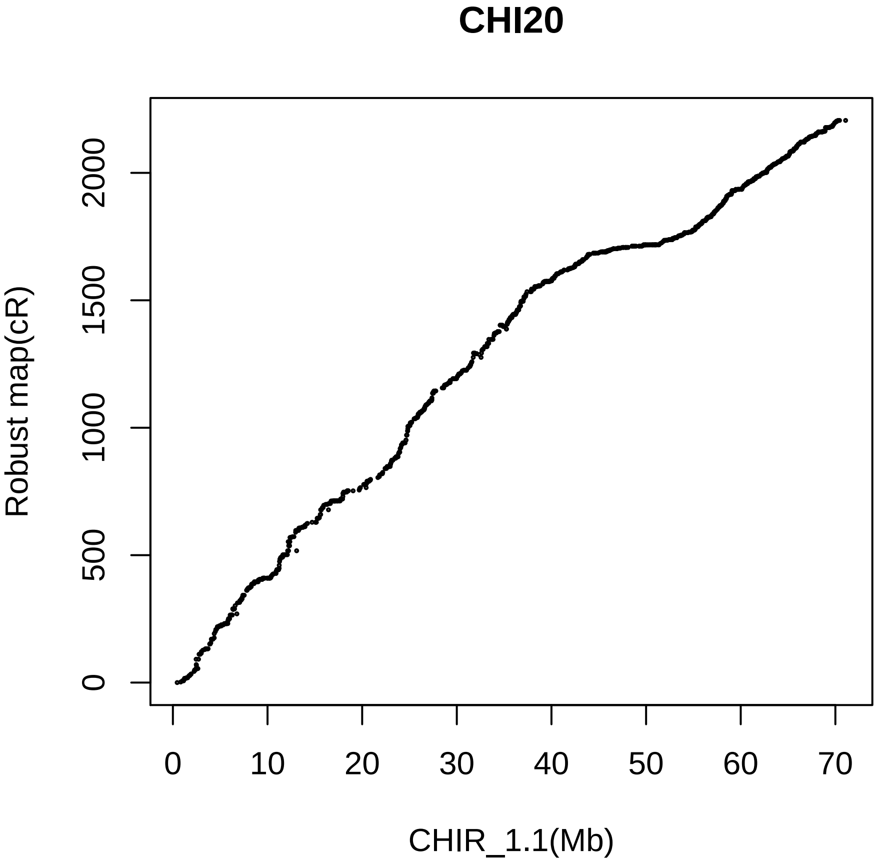

CHI20
Robust map(cR)
0
500
1000
1500
2000
0
10
20
30
40
50
60
70
CHIR_1.1(Mb)

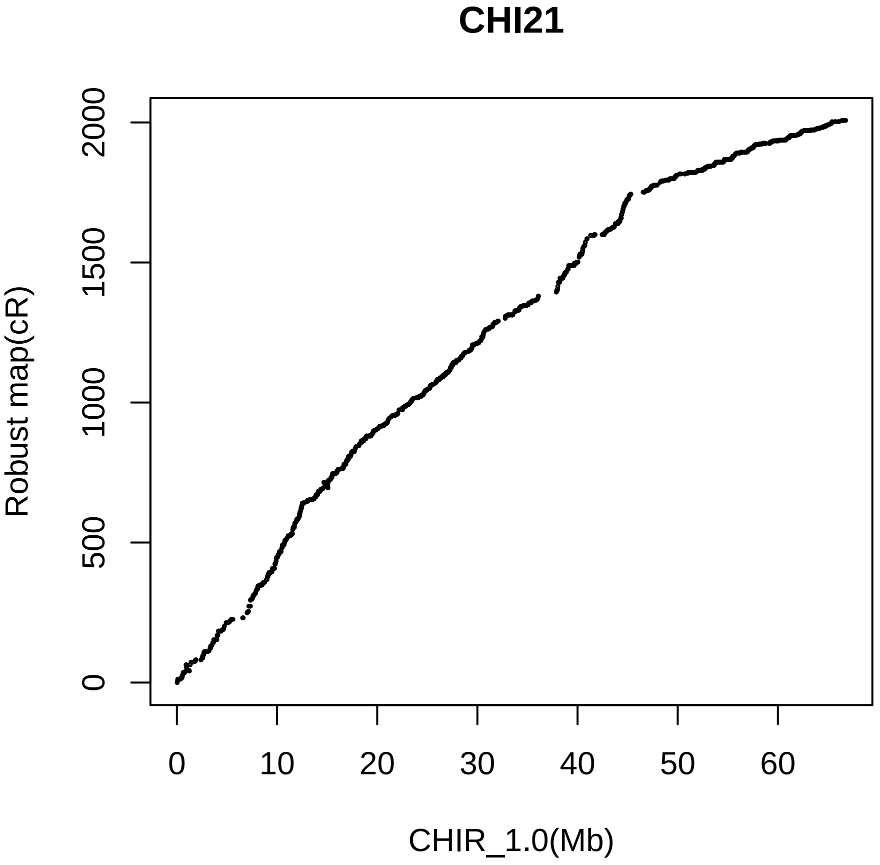
CHI21
Robust map(cR)
0
500
1000
1500
2000
0
10
20
30
40
50
60
CHIR_1.0(Mb)

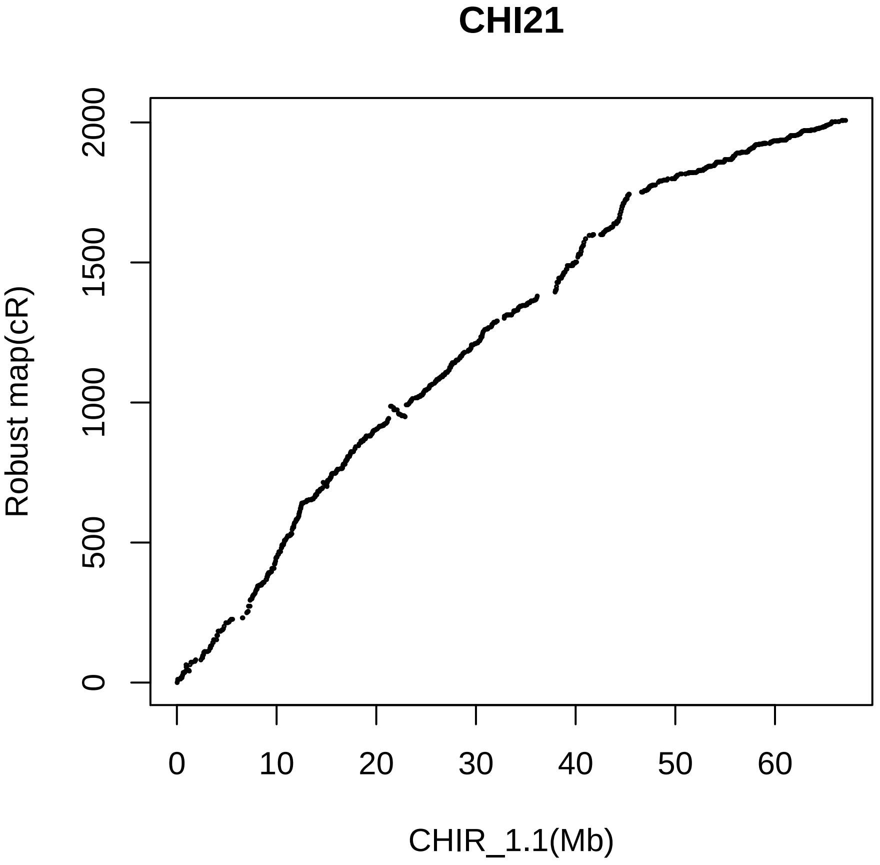
CHI21
Robust map(cR)
0
500
1000
1500
2000
0
10
20
30
40
50
60
CHIR_1.1(Mb)

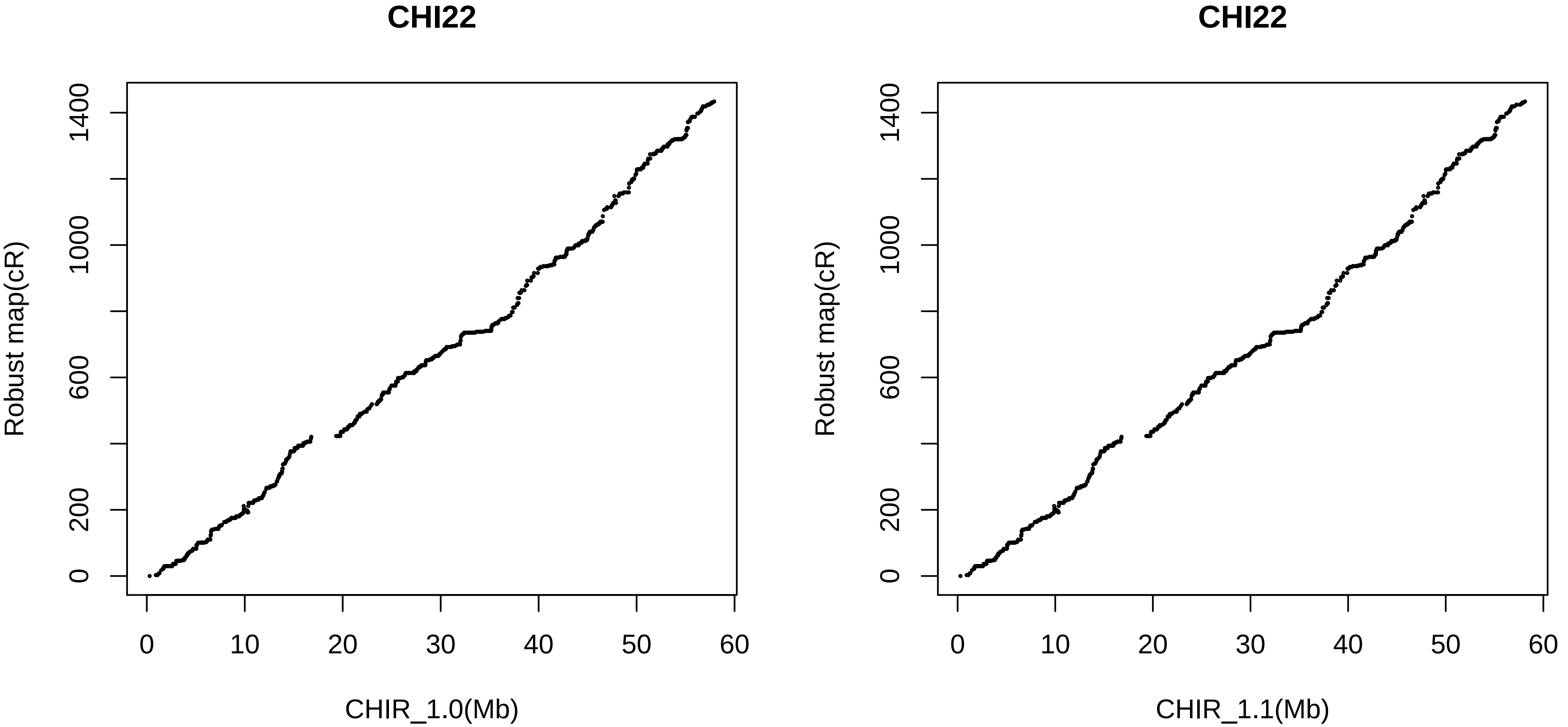
CHI22
Robust map(cR)
0
200
600
1000
1400
0
10
20
30
40
50
60
CHIR_1.0(Mb)
CHI22
Robust map(cR)
0
200
600
1000
1400
0
10
20
30
40
50
60
CHIR_1.1(Mb)

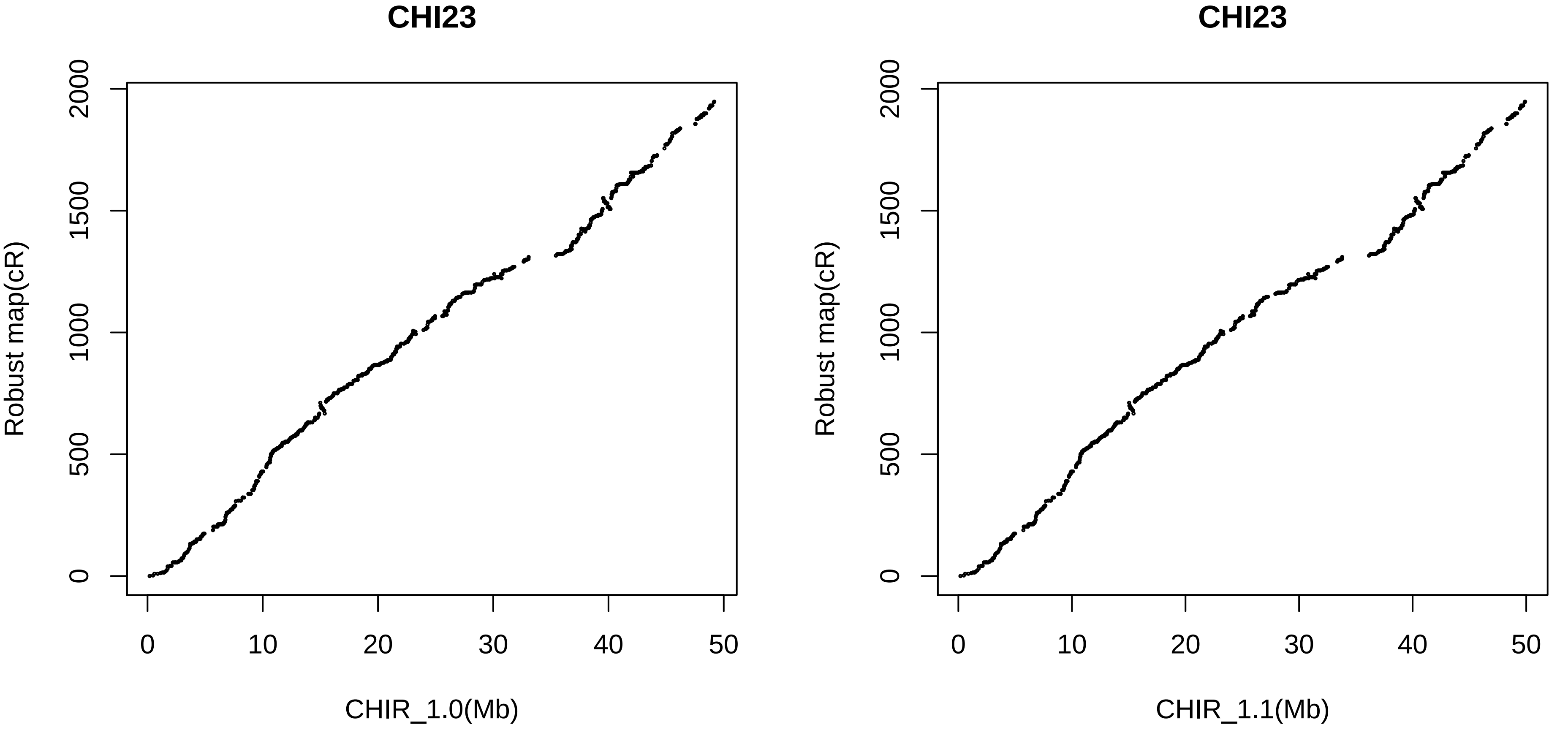
CHI23
Robust map(cR)
0
500
1000
1500
2000
0
10
20
30
40
50
CHIR_1.0(Mb)
CHI23
Robust map(cR)
0
500
1000
1500
2000
0
10
20
30
40
50
CHIR_1.1(Mb)

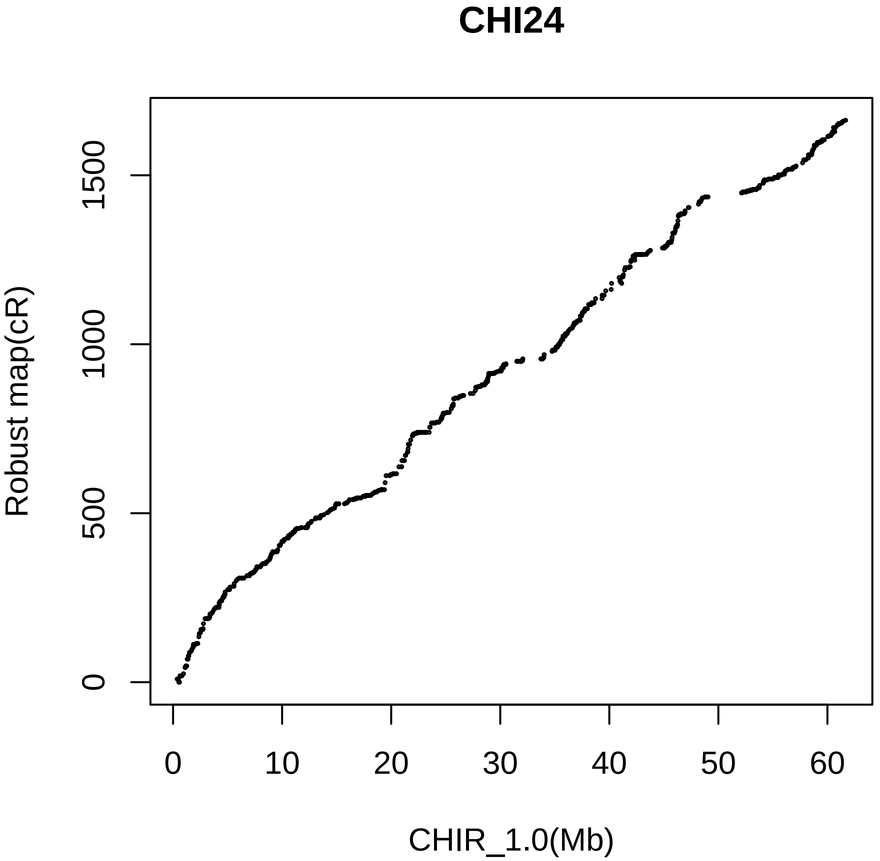
CHI24
Robust map(cR)
0
500
1000
1500
0
10
20
30
40
50
60
CHIR_1.0(Mb)

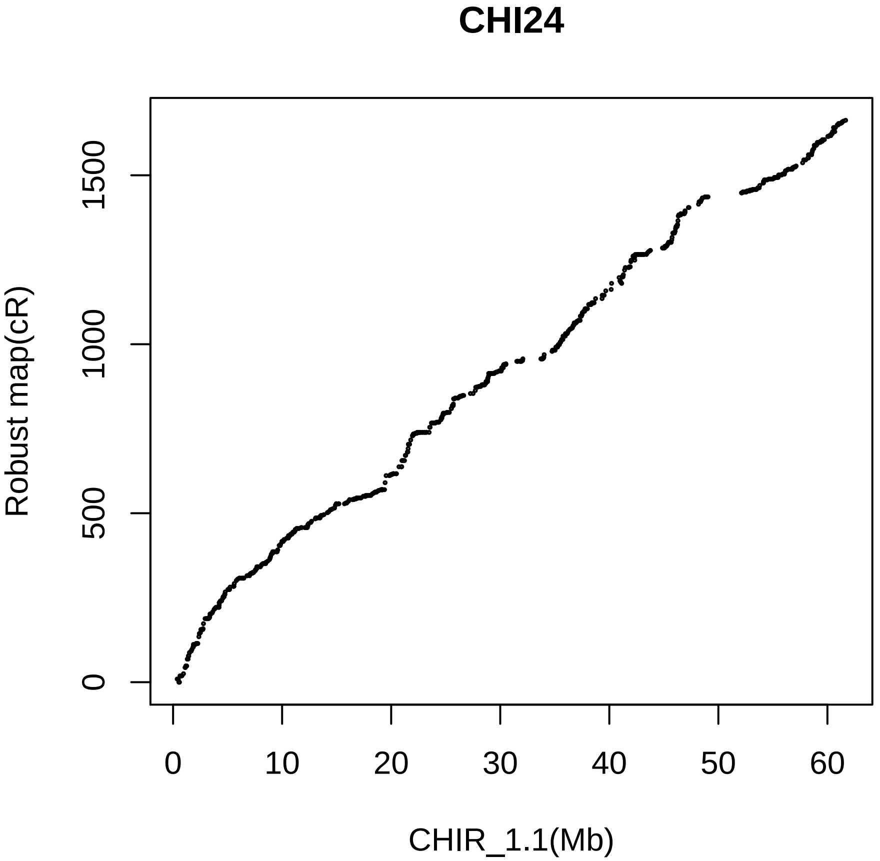
CHI24
Robust map(cR)
0
500
1000
1500
0
10
20
30
40
50
60
CHIR_1.1(Mb)

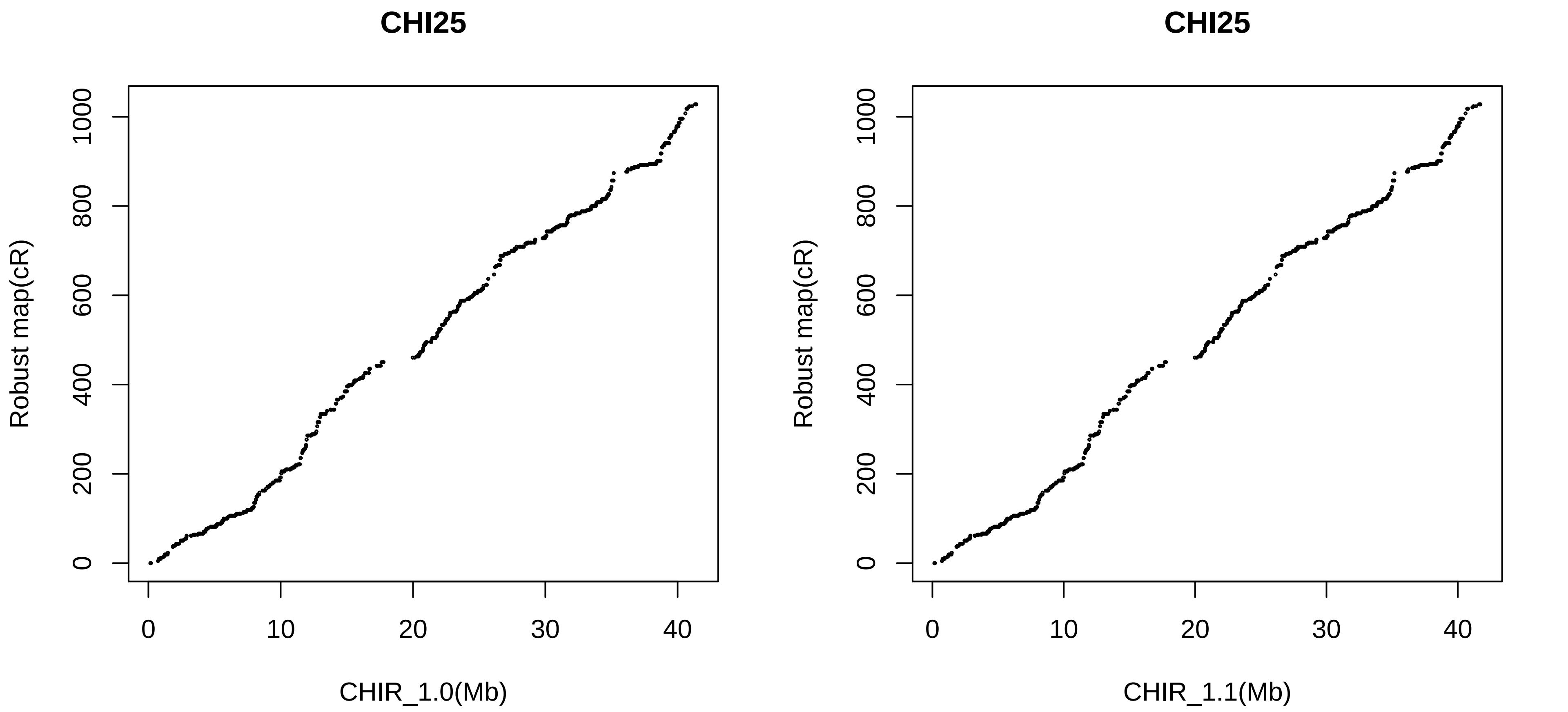

CHI25
Robust map(cR)
CHIR_1.0(Mb)
CHI25
Robust map(cR)
CHIR_1.1(Mb)

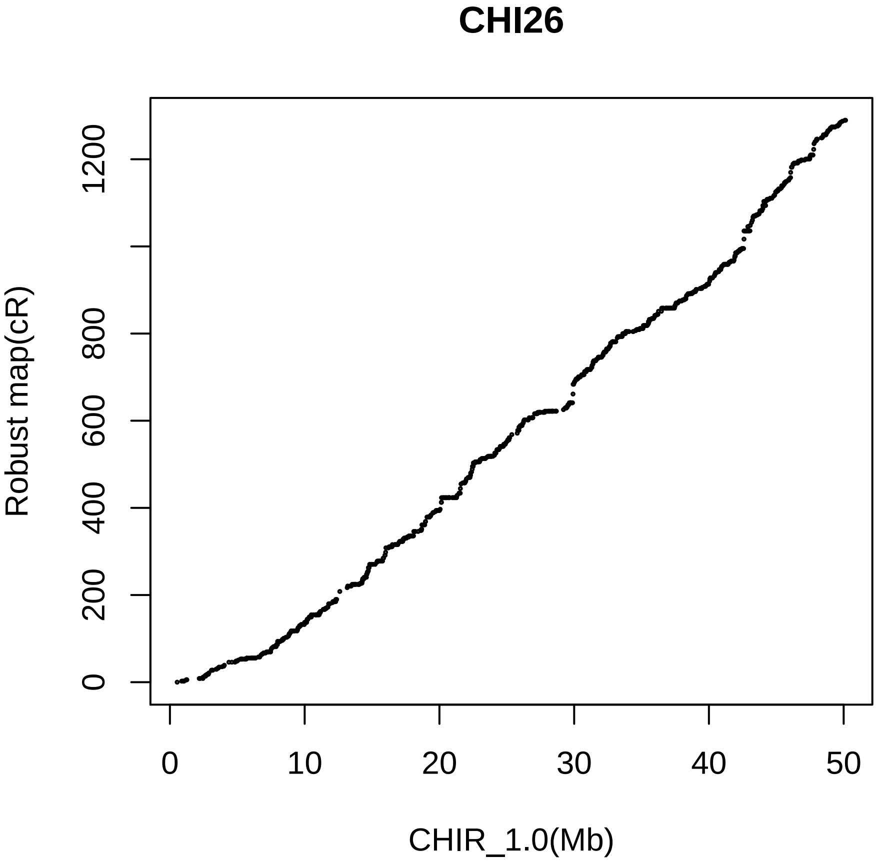
CHI26
Robust map(cR)
0
200
400
600
800
1200
0
10
20
30
40
50
CHIR_1.0(Mb)

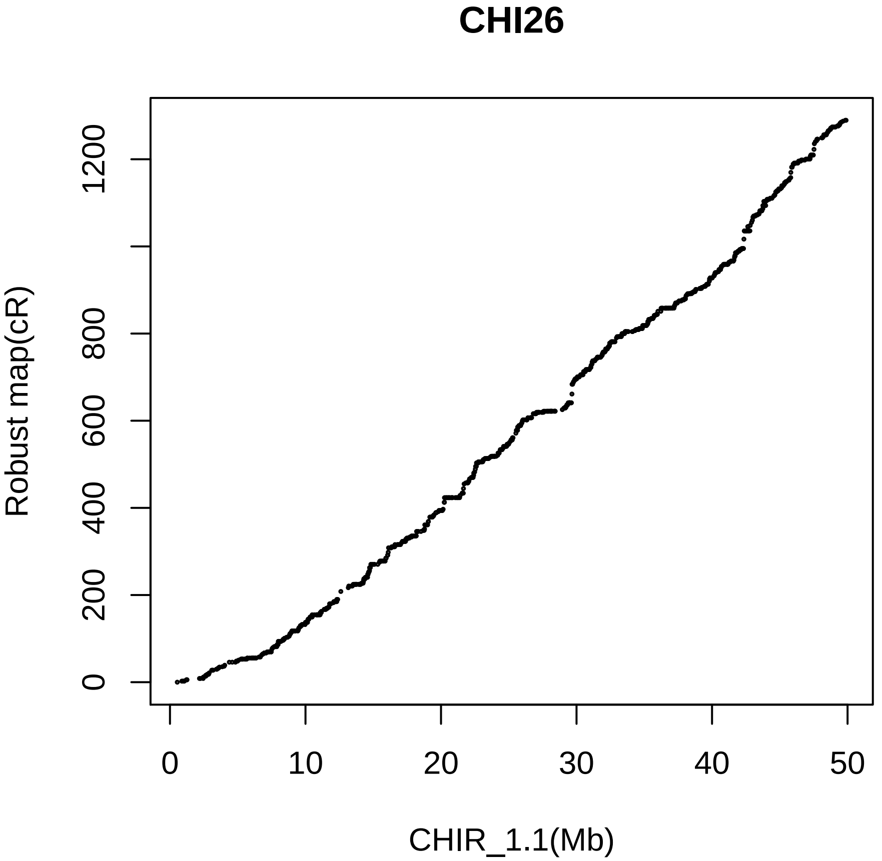
CHI26
Robust map(cR)
0
200
400
600
800
1200
0
10
20
30
40
50
CHIR_1.1(Mb)

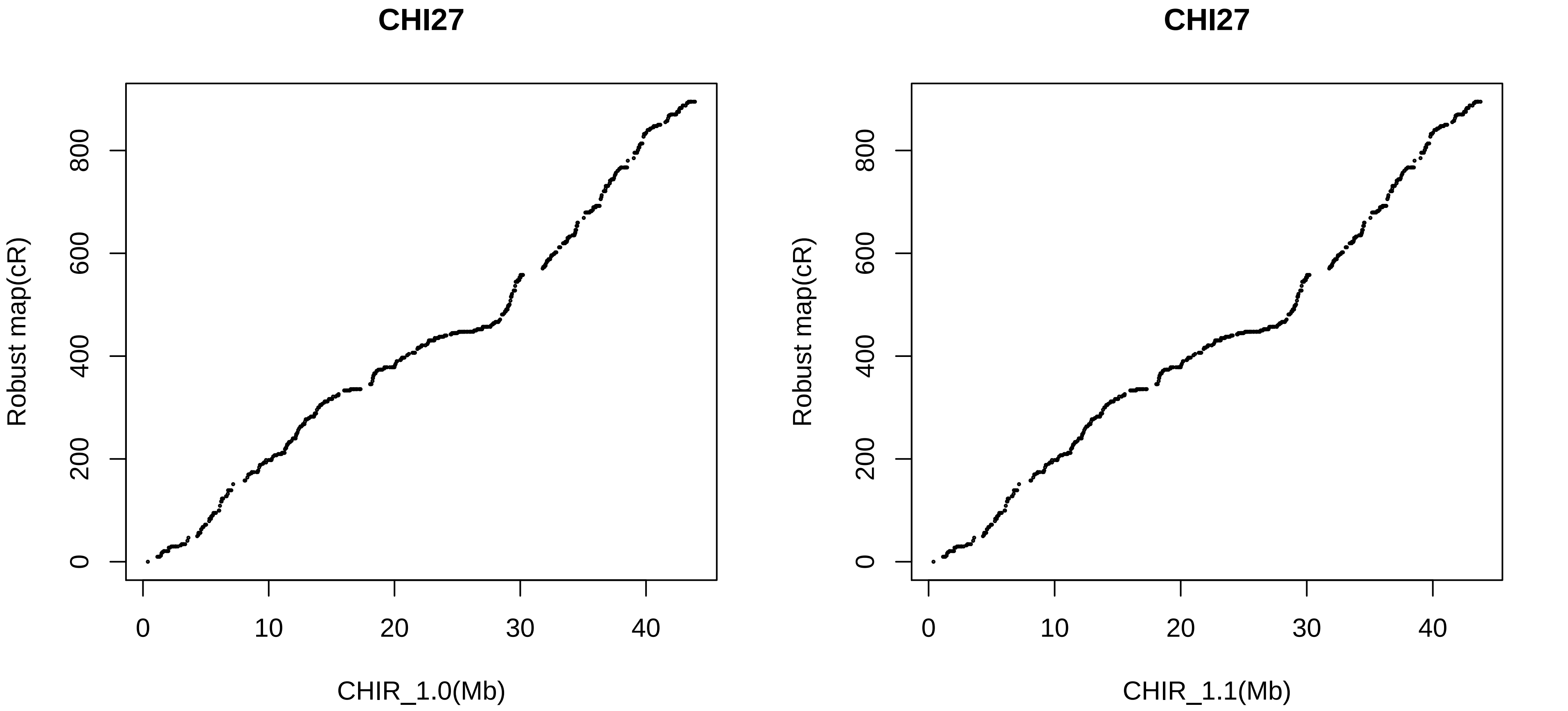
CHI27
Robust map(cR)
0
200
400
600
800
0
10
20
30
40
CHIR_1.0(Mb)
CHI27
Robust map(cR)
0
200
400
600
800
0
10
20
30
40
CHIR_1.1(Mb)

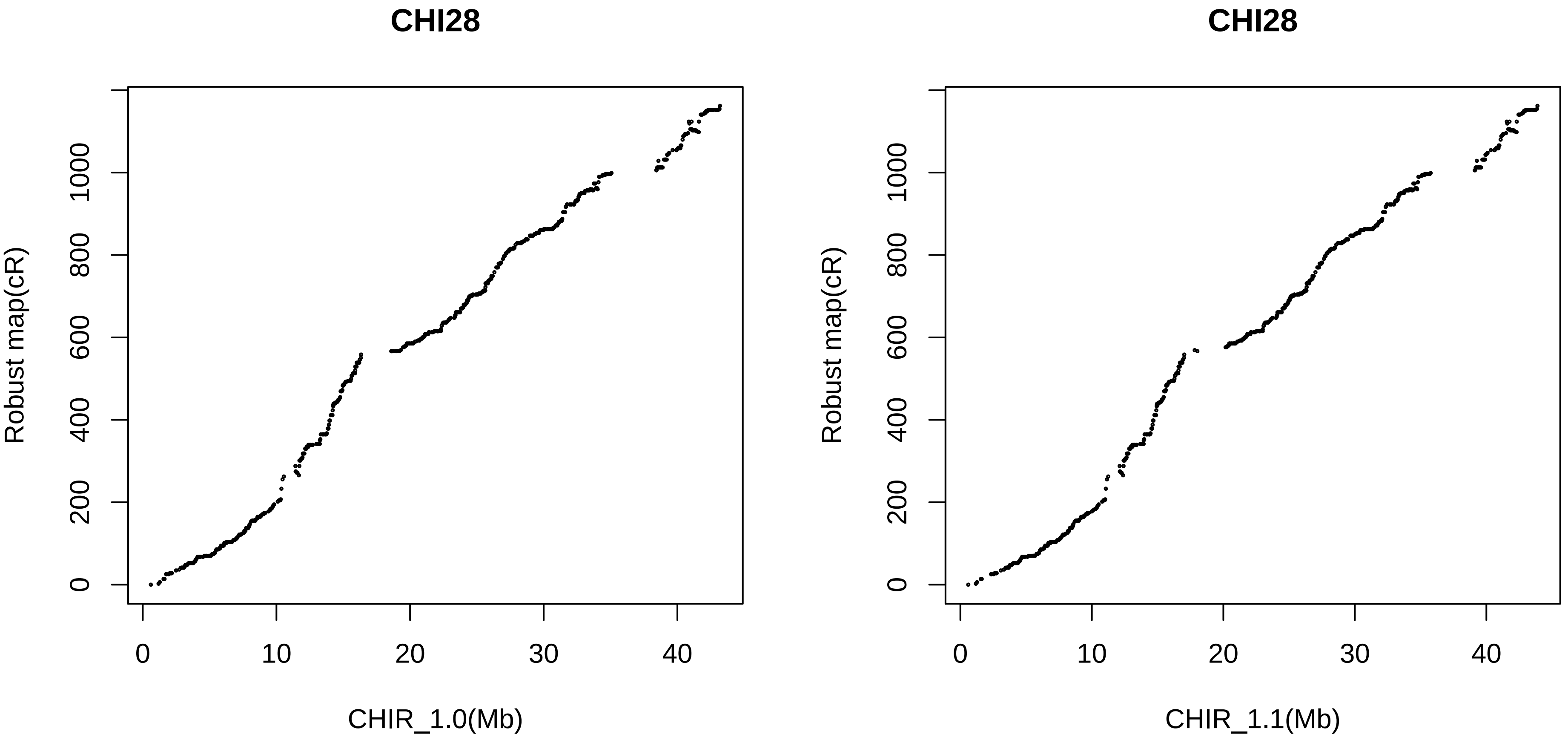
CHI28
Robust map(cR)
0
200
400
600
800
1000
0
10
20
30
40
CHIR_1.0(Mb)
CHI28
Robust map(cR)
0
200
400
600
800
1000
0
10
20
30
40
CHIR_1.1(Mb)

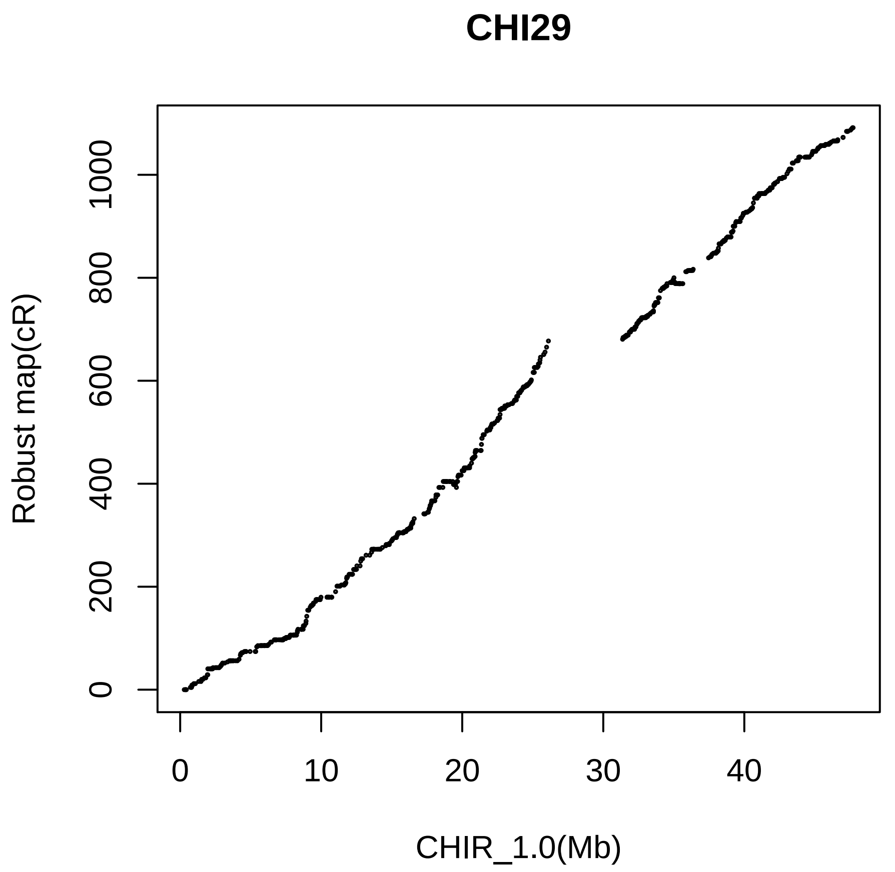
CHI29
Robust map(cR)
0
200
400
600
800
1000
0
10
20
30
40
CHIR_1.0(Mb)

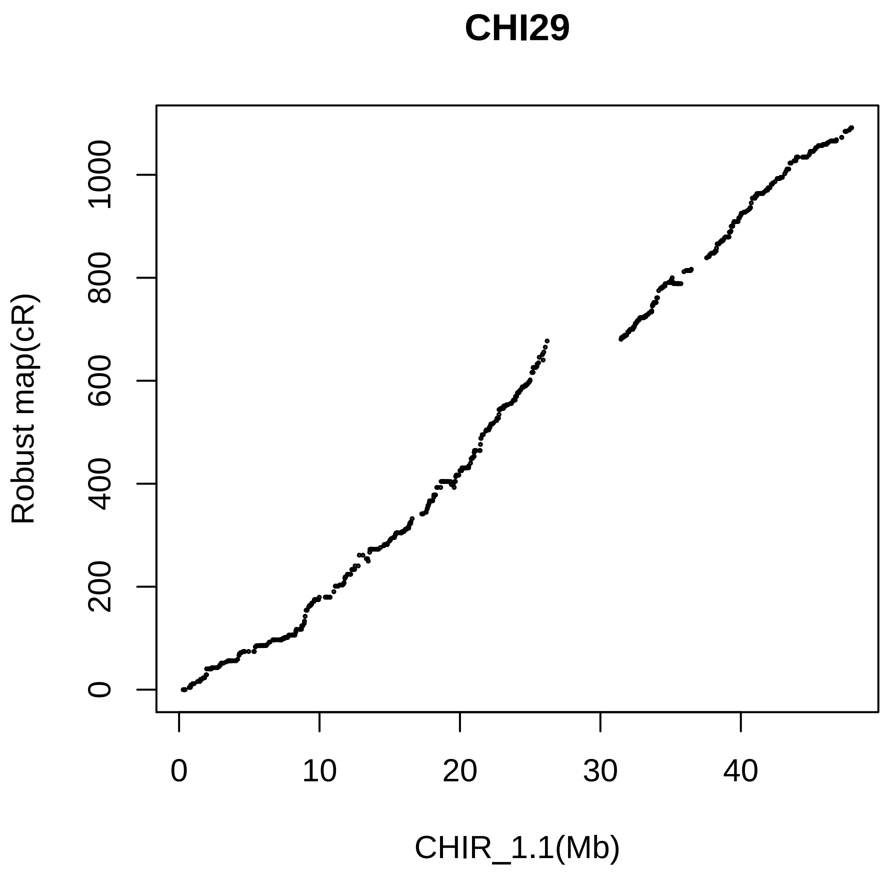
CHI29
Robust map(cR)
0
200
400
600
800
1000
0
10
20
30
40
CHIR_1.1(Mb)

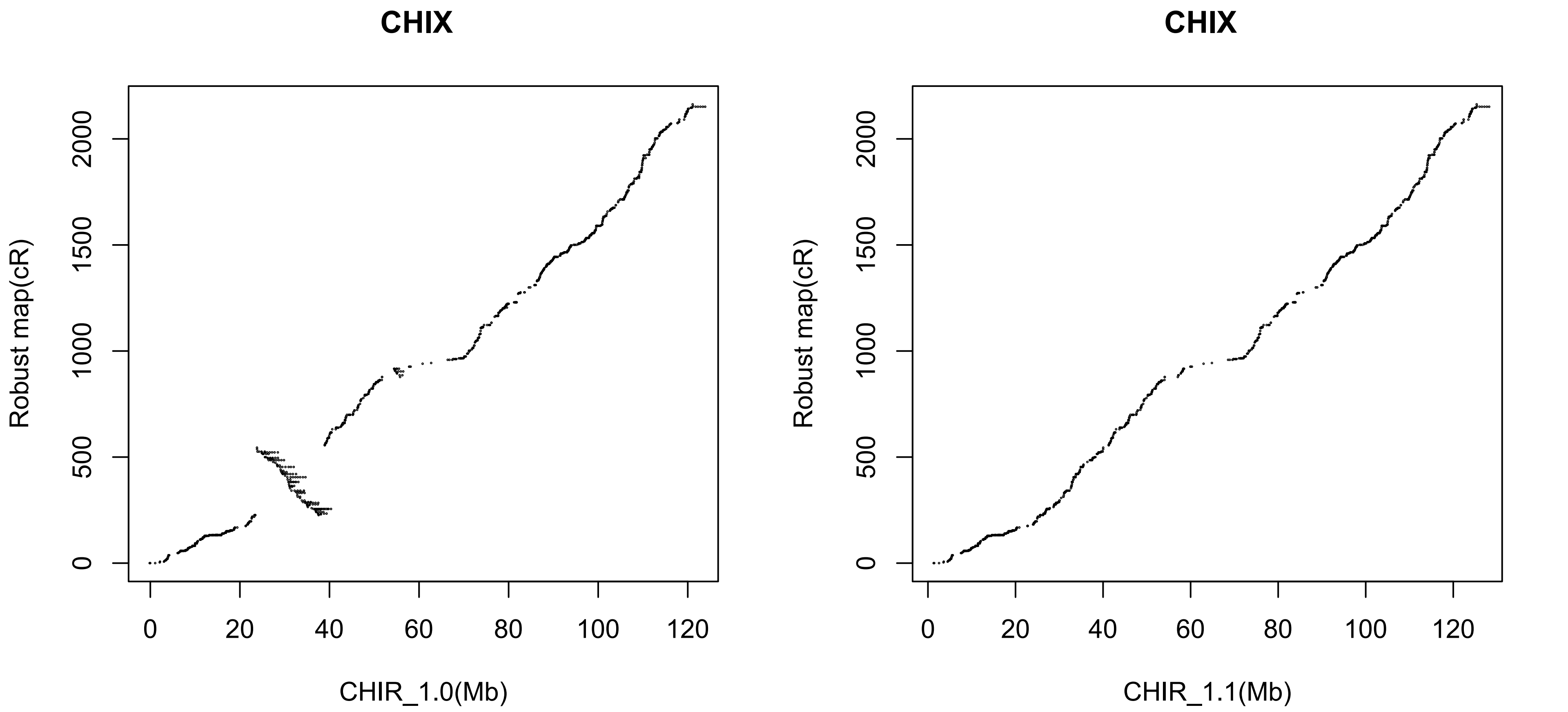

CHIX
Robust map(cR)
CHIR_1.0(Mb)
CHIX
Robust map(cR)
CHIR_1.1(Mb)
