# Supplementary figures and images for: An update of the goat genome assembly using dense radiation hybrid maps allows detailed analysis of evolutionary rearrangements in Bovidae
Source: BMC Genomics. 2014 Jul 23;15(1):625. doi: 10.1186/1471-2164-15-625 (PMC4141111; doi:10.1186/1471-2164-15-625)

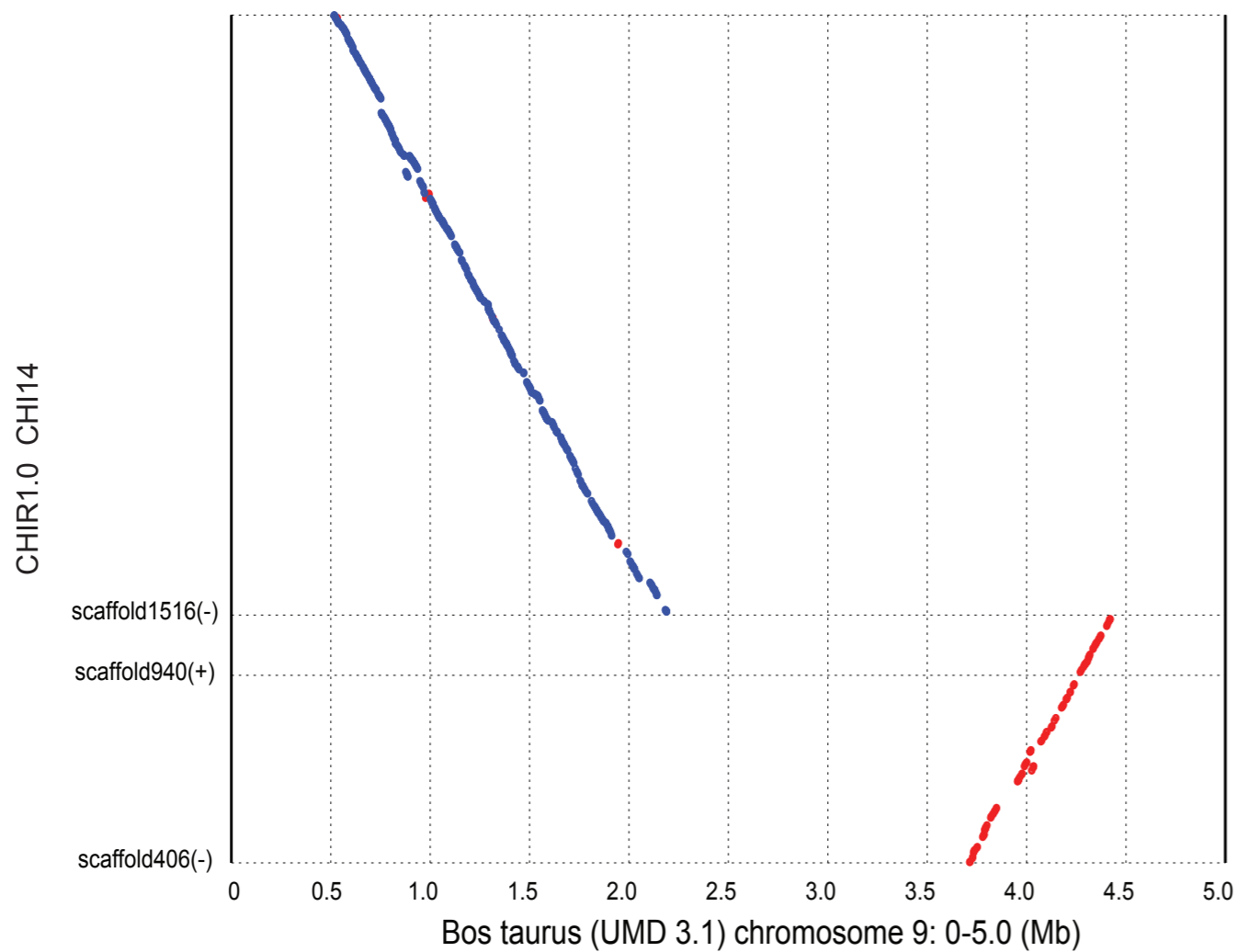

(A)

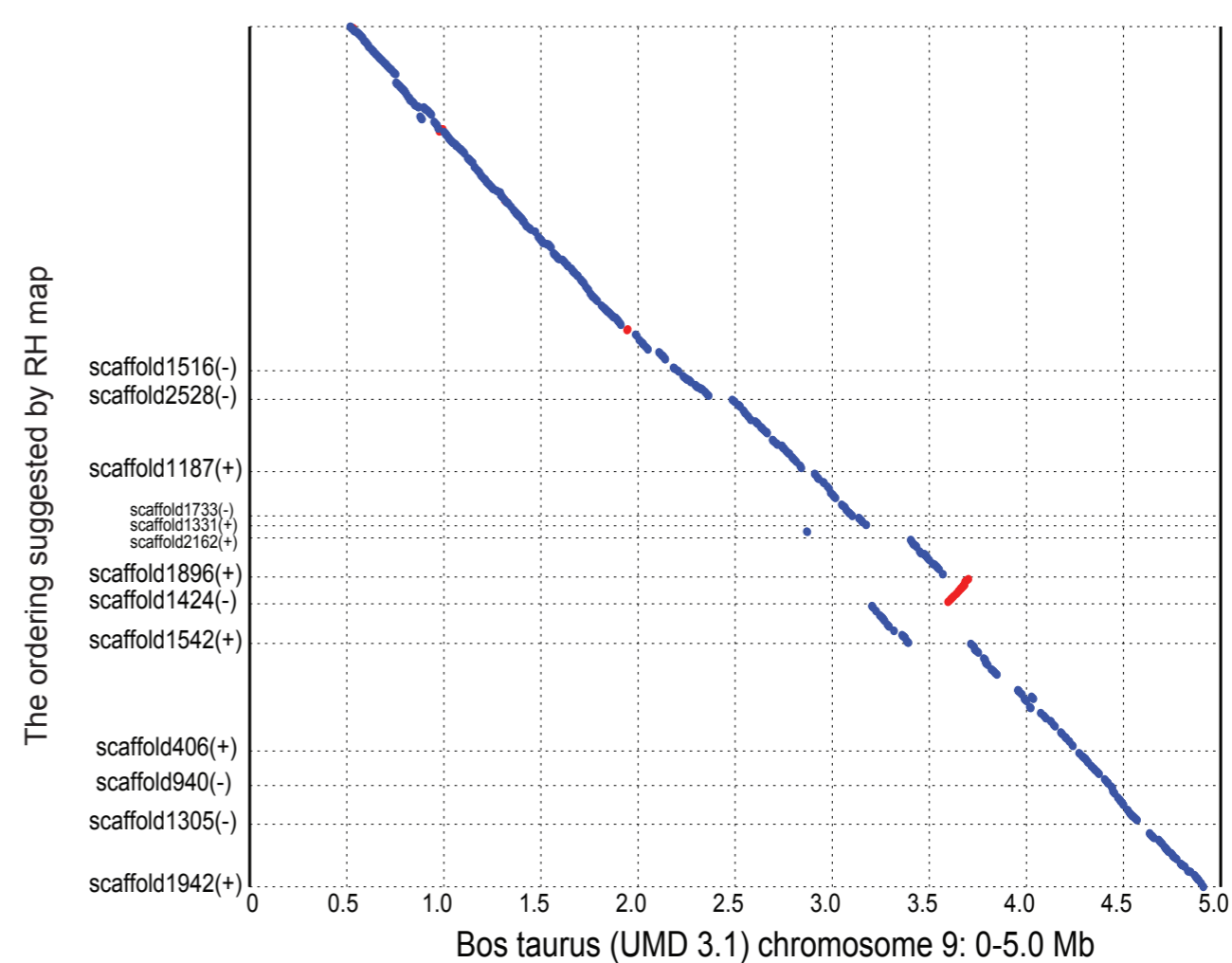

(B)

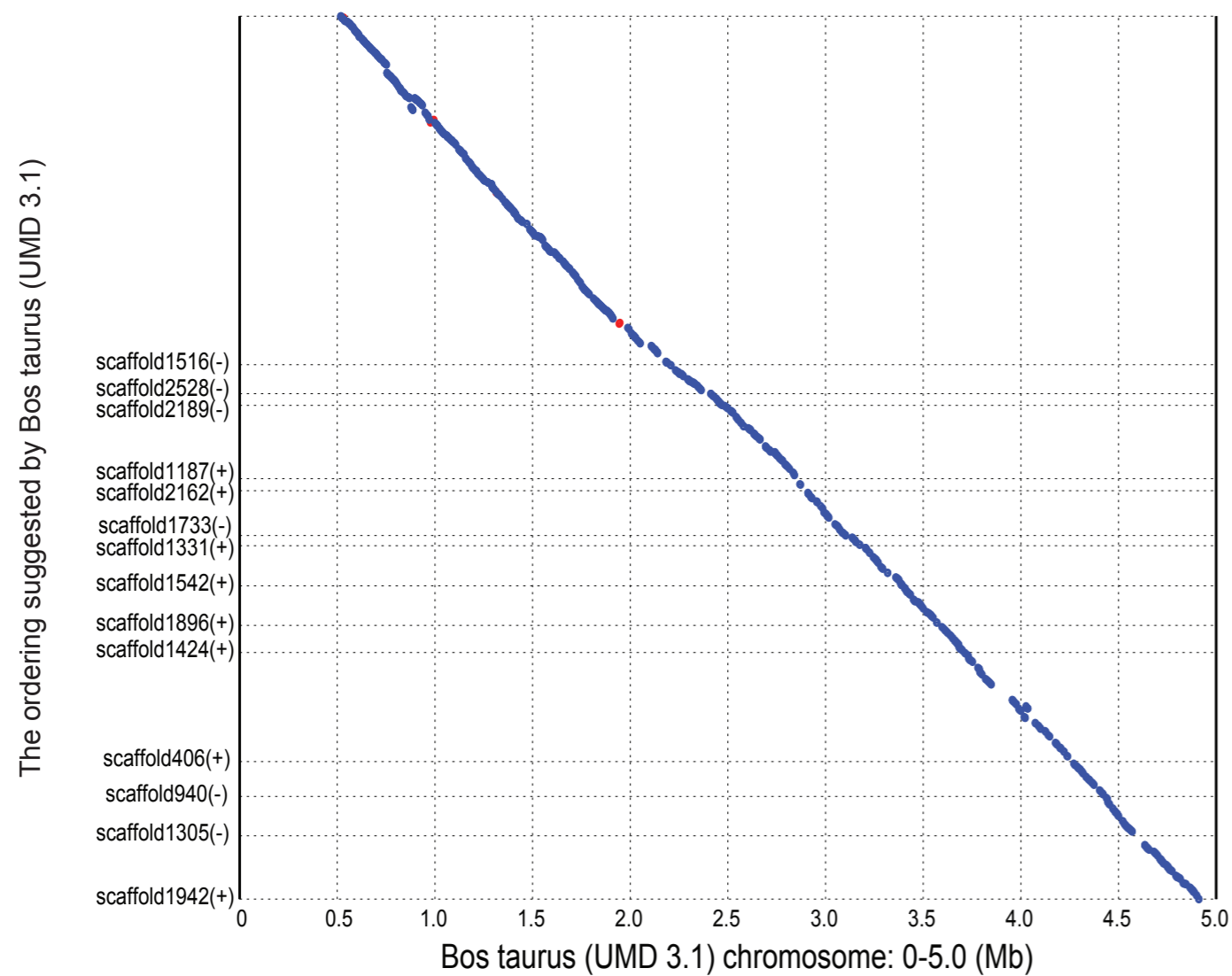

(C)

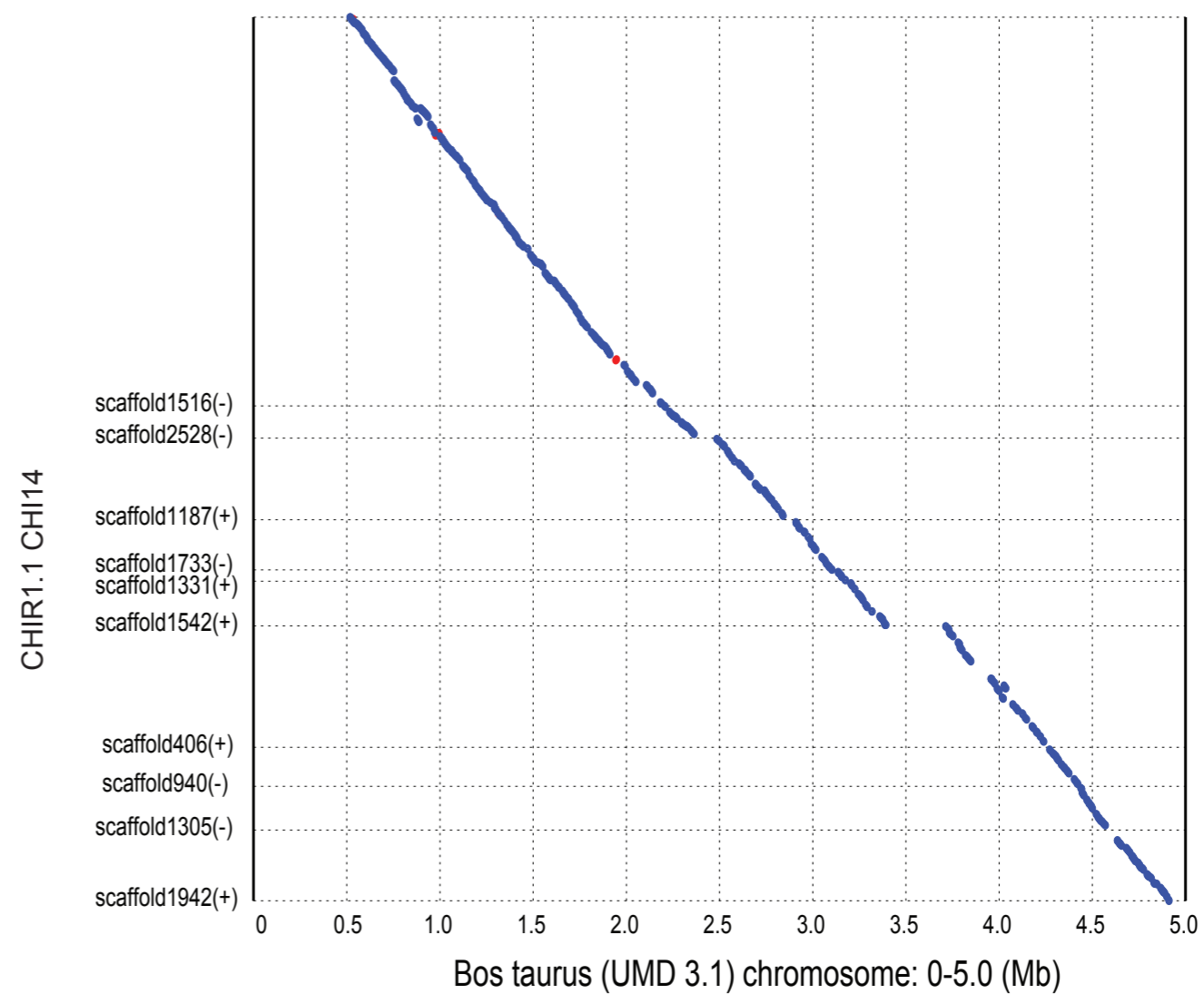

(D)

Supplement: Supplementary file 4 — Additional file 4: Our RH maps can be applied to improve goat sequence map accurately in local region, by both of rectifying the arrangements of scaffolds and adding the unplaced scaffolds. Forward alignments are plotted as red lines/dots while inverse (reverse compliment) alignments are plotted as blue lines/dots. Panel A: The region from 8.3 Mb to 8.9 Mb of CHI14 [where the order of scaffolds is scaffold406 (−), scaffold940 (+), scaffold1516 (−)], showed an inversion and two deletions with cattle genome, that presents either true chromosome evolution or artificial errors. Panel B: The RH map suggested an order of scaffolds in the region [scaffold1942(+), scaffold1305(−), scaffold940(−), scaffold406(+), scaffold1542(+), scaffold1424(−), scaffold1896(+), scaffold2162(+), scaffold1331(+), scaffold1733(−), scaffold1187(+), scaffold2528(−), scaffold1516(−)]. The new sequence, ordered using our RH map showed good colinearity with the cattle genome, demonstrating that the inversion and the two deletions were artificial errors. Thus, the goat sequence can be improved. Panel C: The cattle genome suggested an order of scaffolds in the region. The information of conserved syntenies is used to the new order of scaffold by RH map. Panel D: The new ordering of scaffolds excluded three dubious scaffolds (scaffold1424, scaffold1896, and scaffold2162) and is used to the assembly CHIR_1.0. (PDF 200 KB) [file 12864_2013_6362_MOESM4_ESM.pdf]

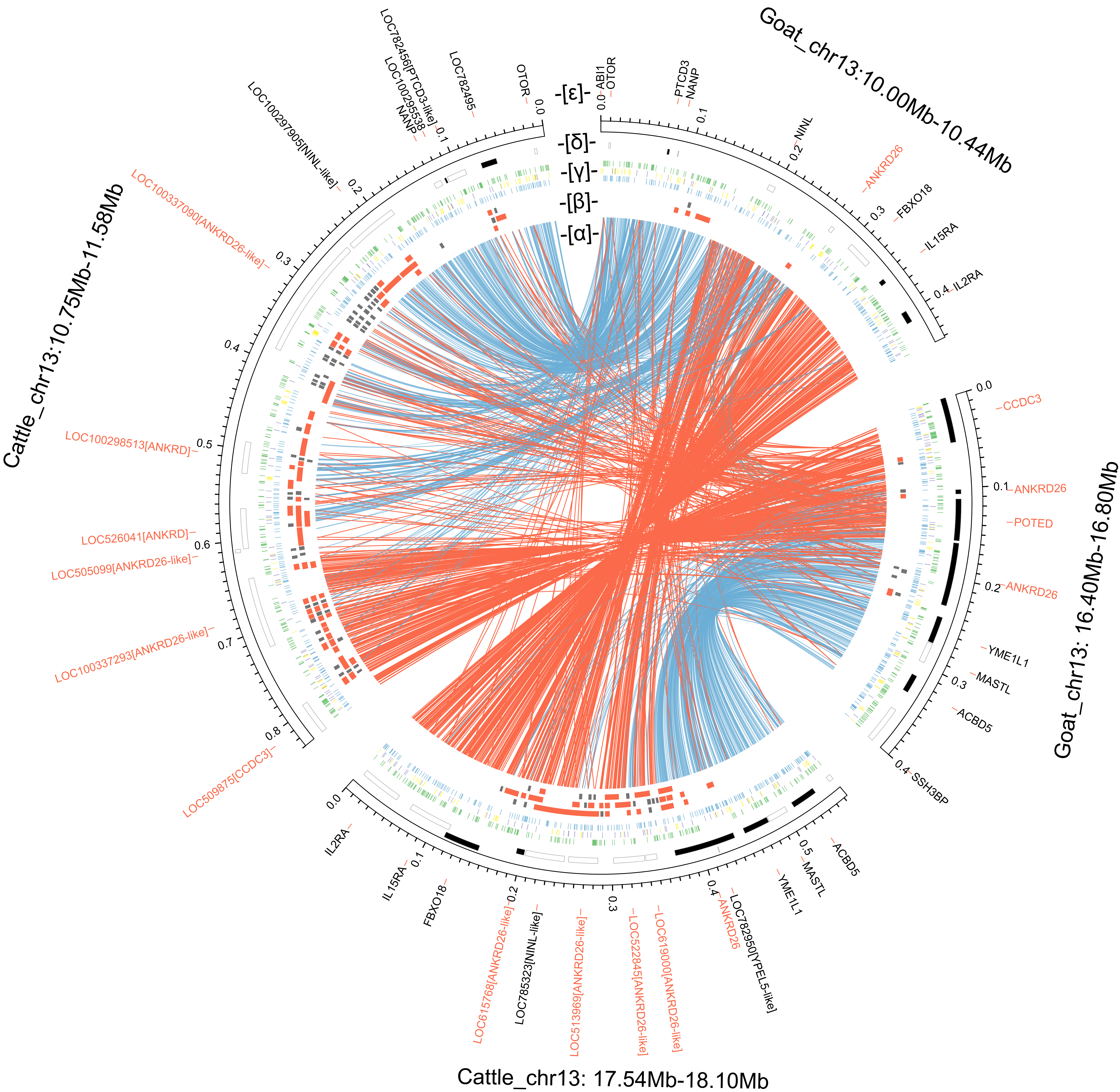

Supplement: Supplementary file 6 — Additional file 6: Sequence architecture at synteny breaks in chromosome 13 between cattle and goat. [α] Goat-cattle pair-wise alignments are highlighted by red and green colors; Self-comparisons are in grey. [β] Segmental duplications. [γ] LINEs (green), LTRs (yellow), SINEs (purple), and Simple repeats (blue). [δ] Genes with colors denotes transcriptional orientation (the white represents “+” and the black represent “-“). [ϵ] Names of ANKRD26 homologs are marked in red. (PDF 557 KB) [file 12864_2013_6362_MOESM6_ESM.pdf]

**POTE family**

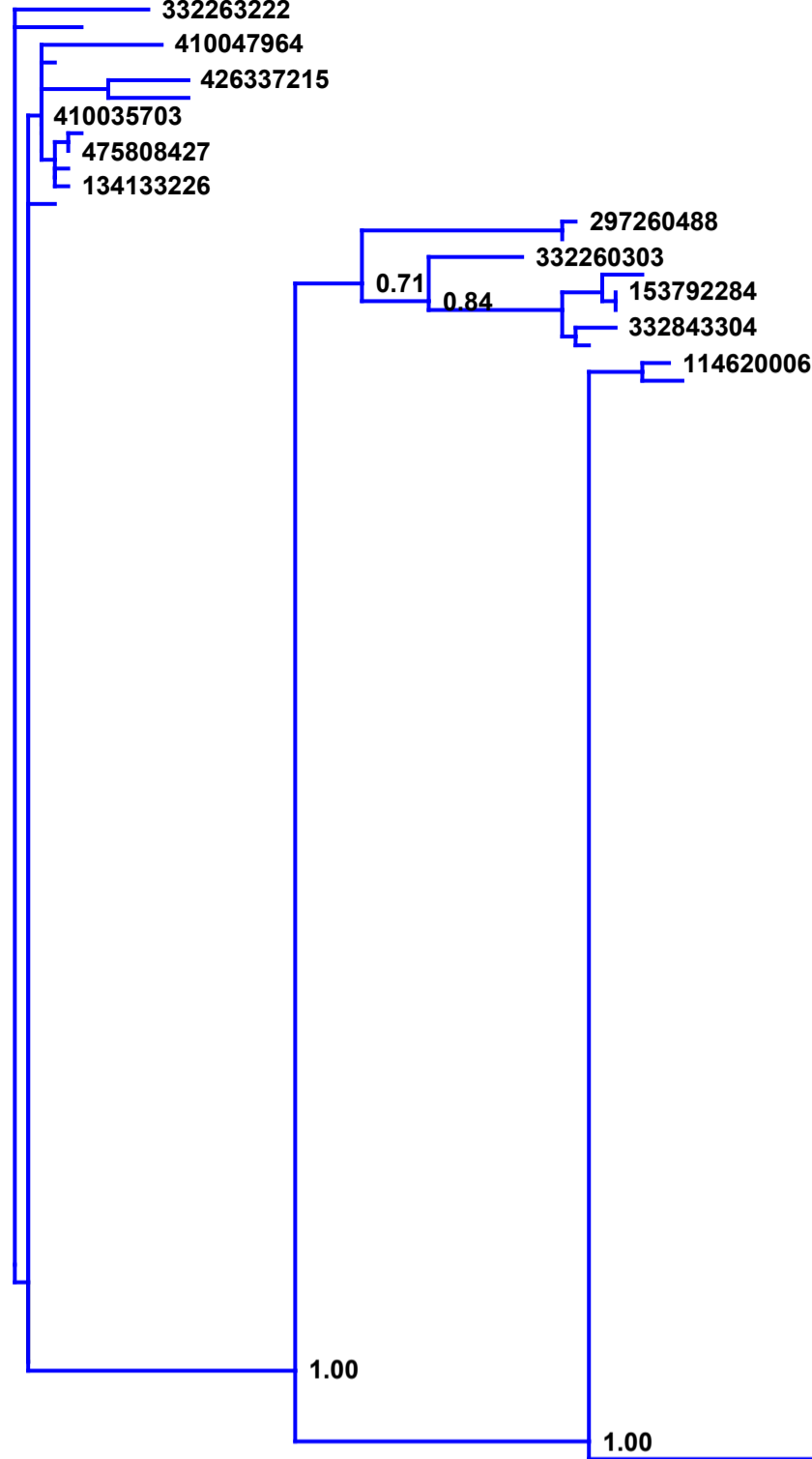

**ANKED36 family**

**ANKED62 family**

**ANKRD20 family**

**ANKRD18 family**

**ancient ANKRD26-like family**

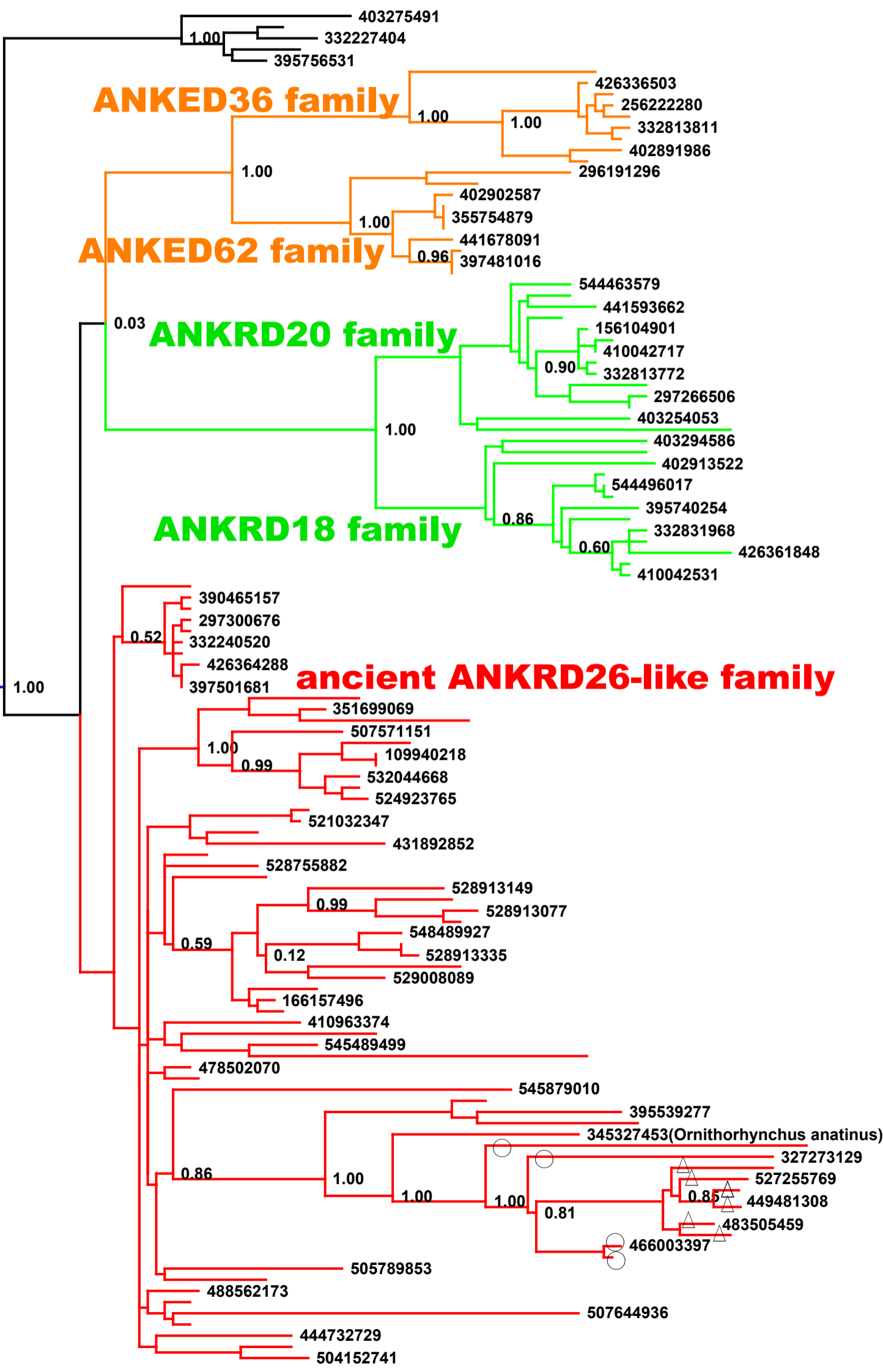

Supplement: Supplementary file 7 — Additional file 7: A phylogenetic tree including 142 proteins of ANKRD26 homologs in mammals (132 proteins), in birds (6 proteins) and in reptilians (4 proteins). Proteins of GI number were clustered in four groups of ancient ANKRD26-like (red), ANKRD18/ANKRD20 (green), ANKRD36/ANKRD62 (orange), and POTE (blue). The proteins of birds and those of reptilians are marked as triangles and circles, respectively. (PDF 158 KB) [file 12864_2013_6362_MOESM7_ESM.pdf]

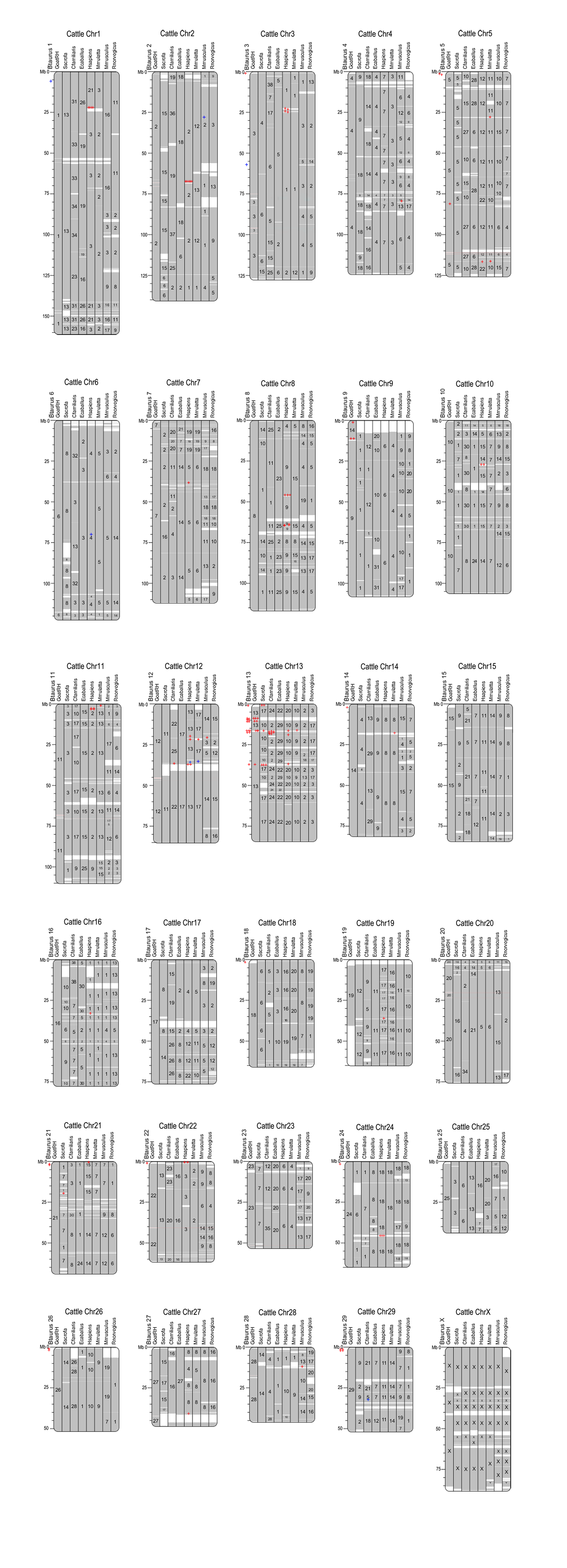

Supplement: Supplementary file 8 — Additional file 8: Cattle chromosomes are used as reference chromosomes and are respectively compared to goat RH maps, pig ( S. scrofa ), horse ( E. caballus ), dog ( C. familiaris ), human ( H. sapiens ), macaque ( M. mulatta ), mouse ( M. musculus ), and rat ( R. norvegicus ) in the Evolution Highyway program. We detected the genomic location of 115 members of ANKRD26 family in nine mammalian genome (excluding unmapped scaffolds), of which 109 locate in breakpoints (red cross) and 6 locate in homologous synteny (blue cross). Most of the members expanded specifically in species and lineage specific evolutionary breakpoints or in centromeres. The duplication of ANKRD26 gene follows the patterns of convergent breakpoint reuse through chromosome evolution. (TIFF 2 MB) [file 12864_2013_6362_MOESM8_ESM.tiff]
