# Supplementary material for: An update of the goat genome assembly using dense radiation hybrid maps allows detailed analysis of evolutionary rearrangements in Bovidae
Source: BMC Genomics. 2014 Jul 23;15(1):625. doi: 10.1186/1471-2164-15-625 (PMC4141111; doi:10.1186/1471-2164-15-625)

Chromosome 1

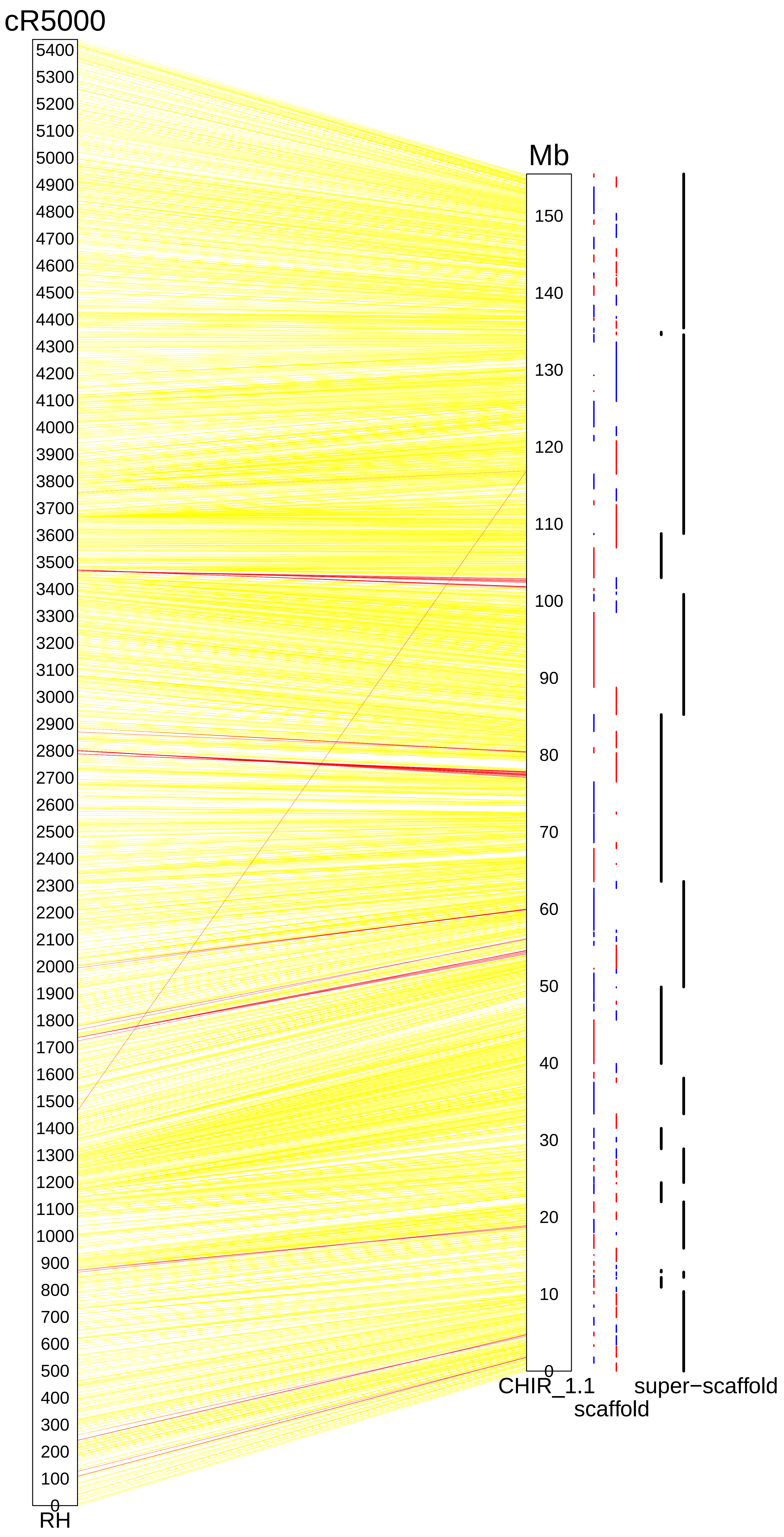

Chromosome 2

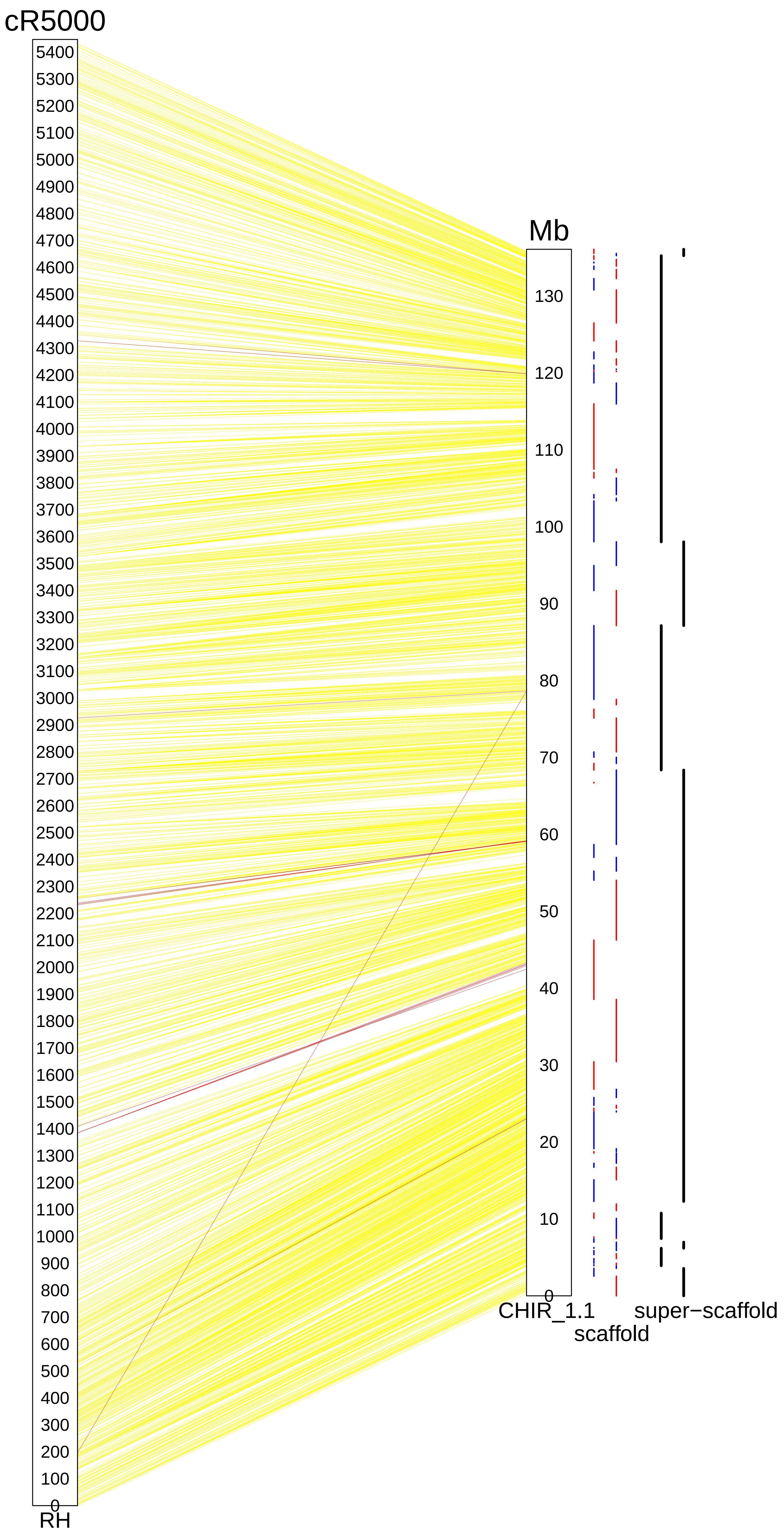

Chromosome 3

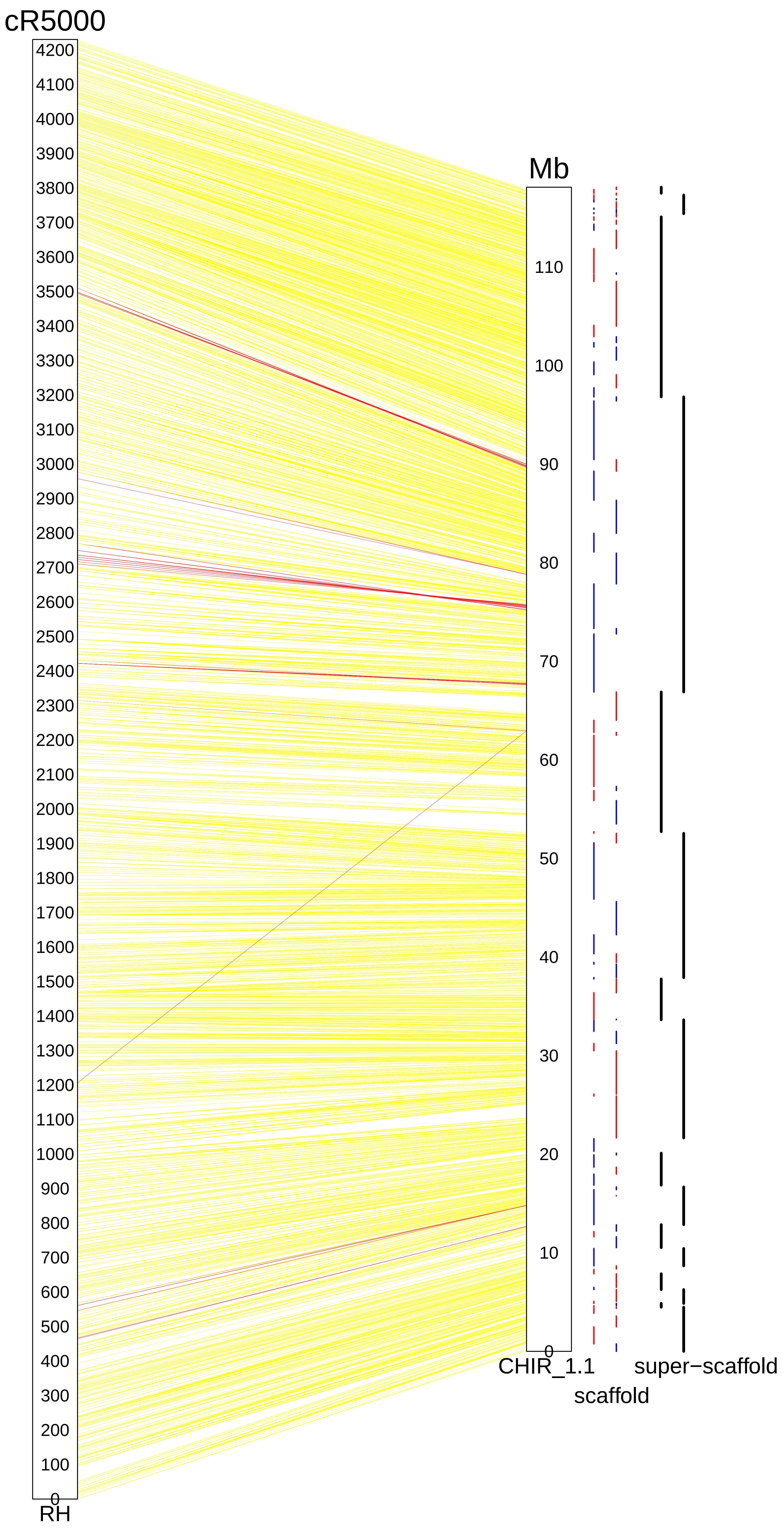

Chromosome 4

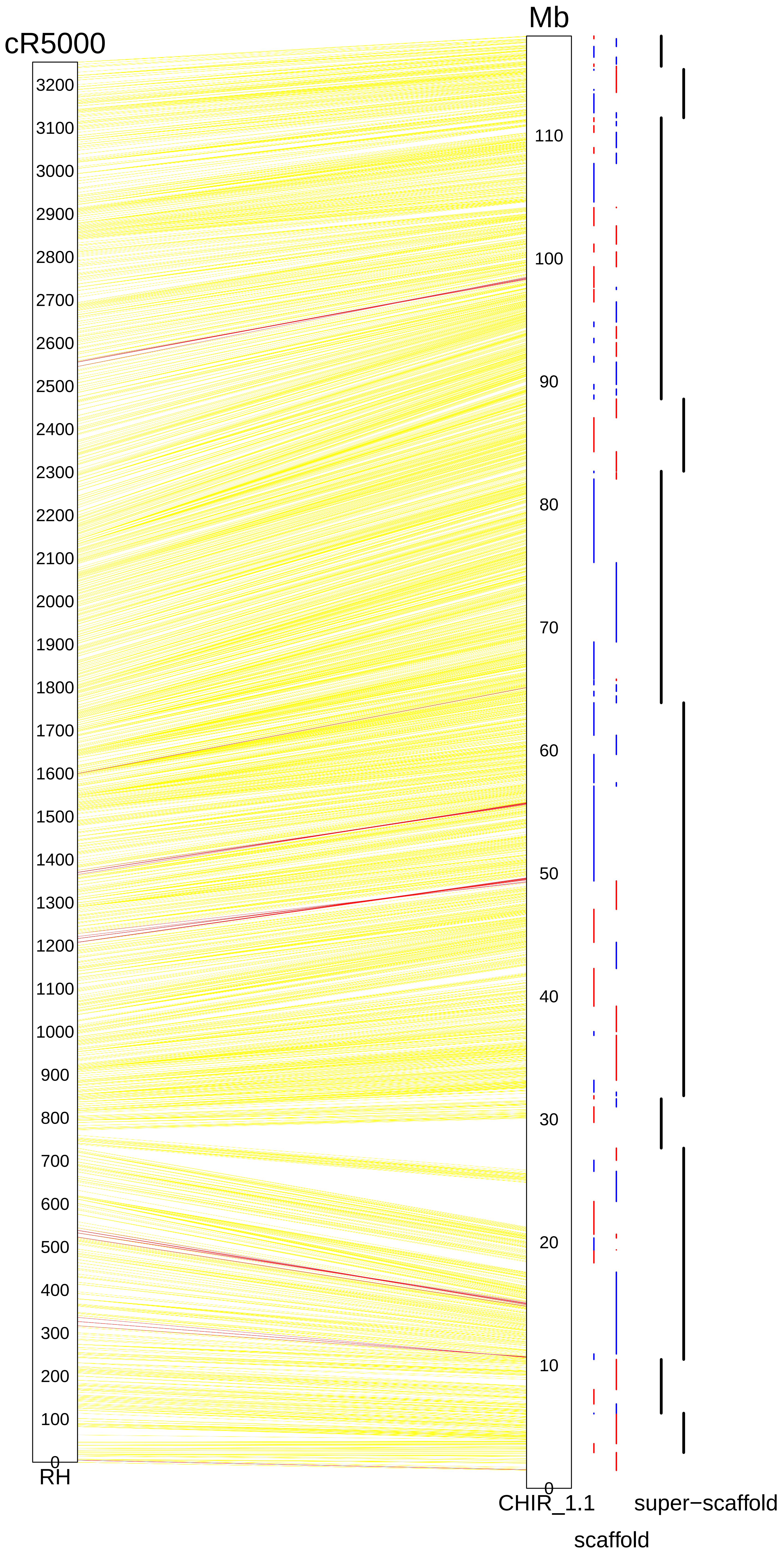

Chromosome 5

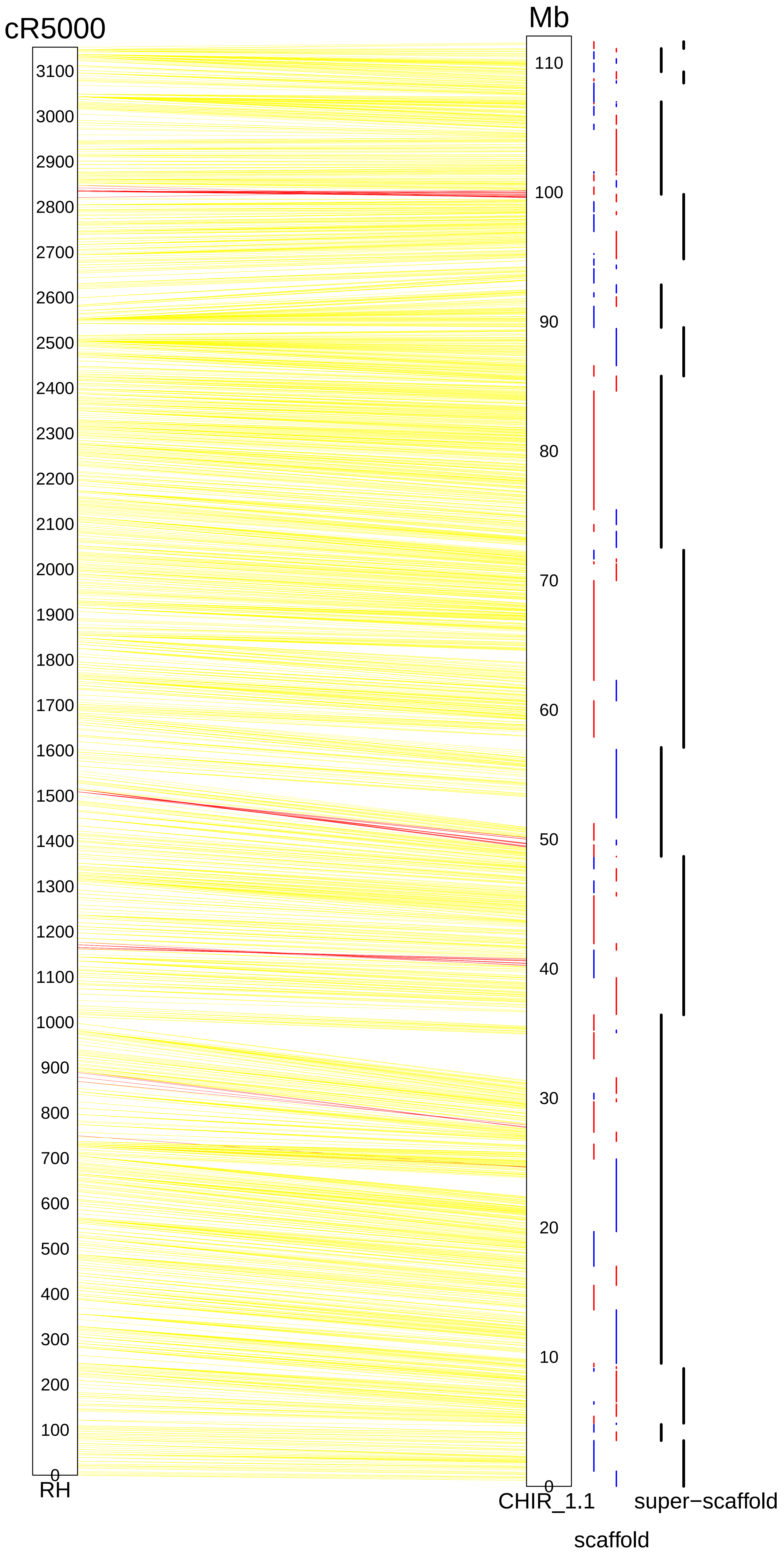

Chromosome 6

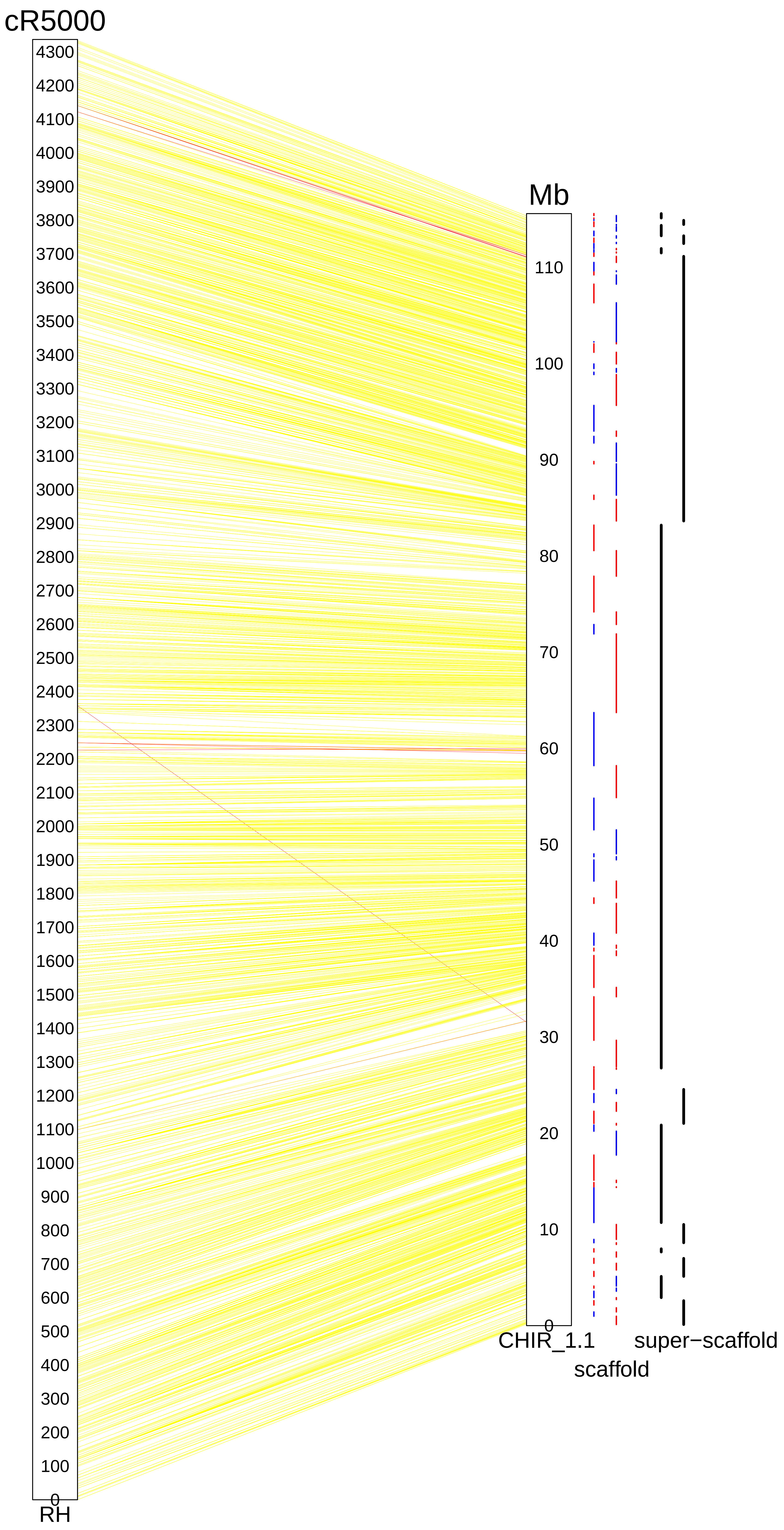

Chromosome 7

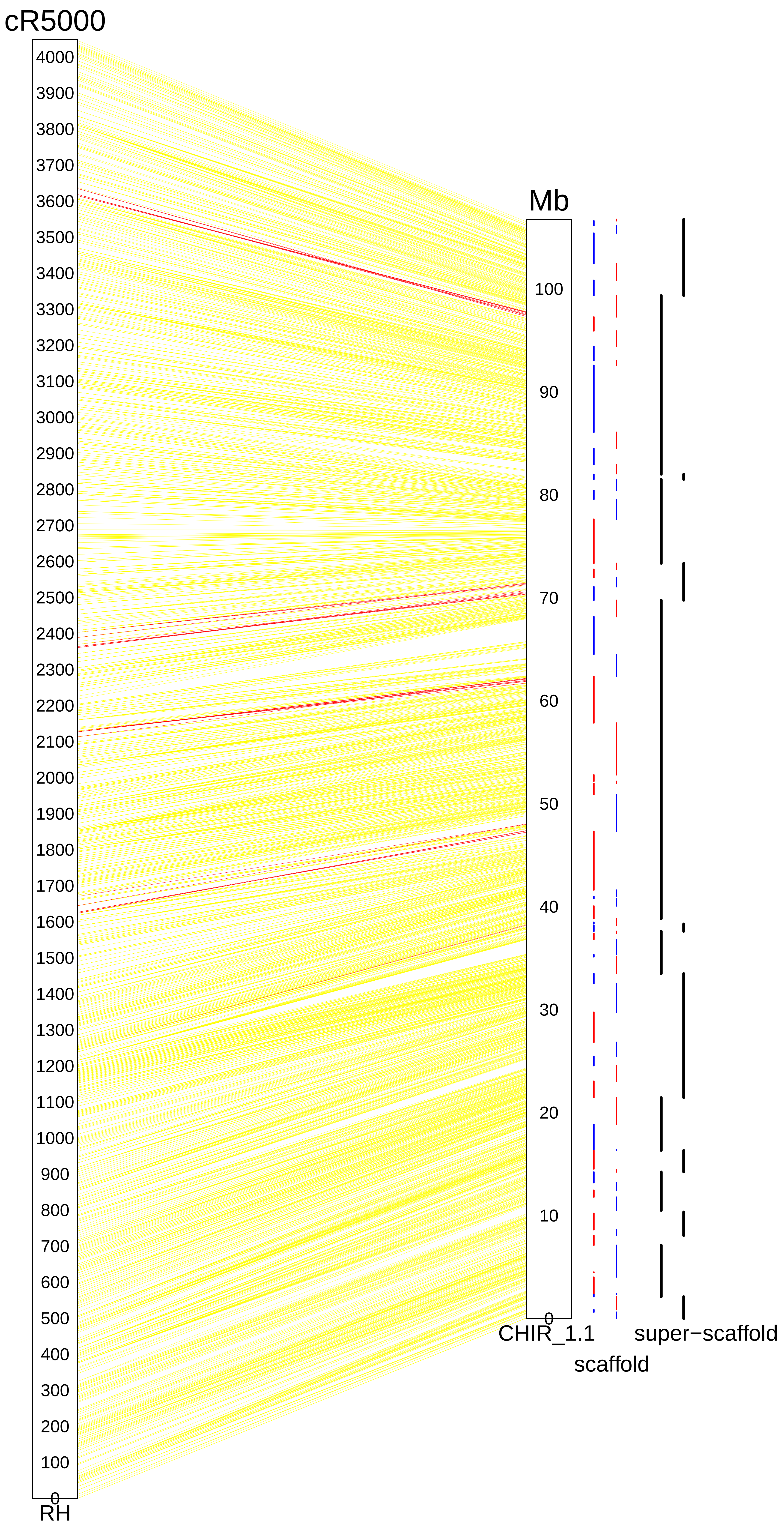

Chromosome 8

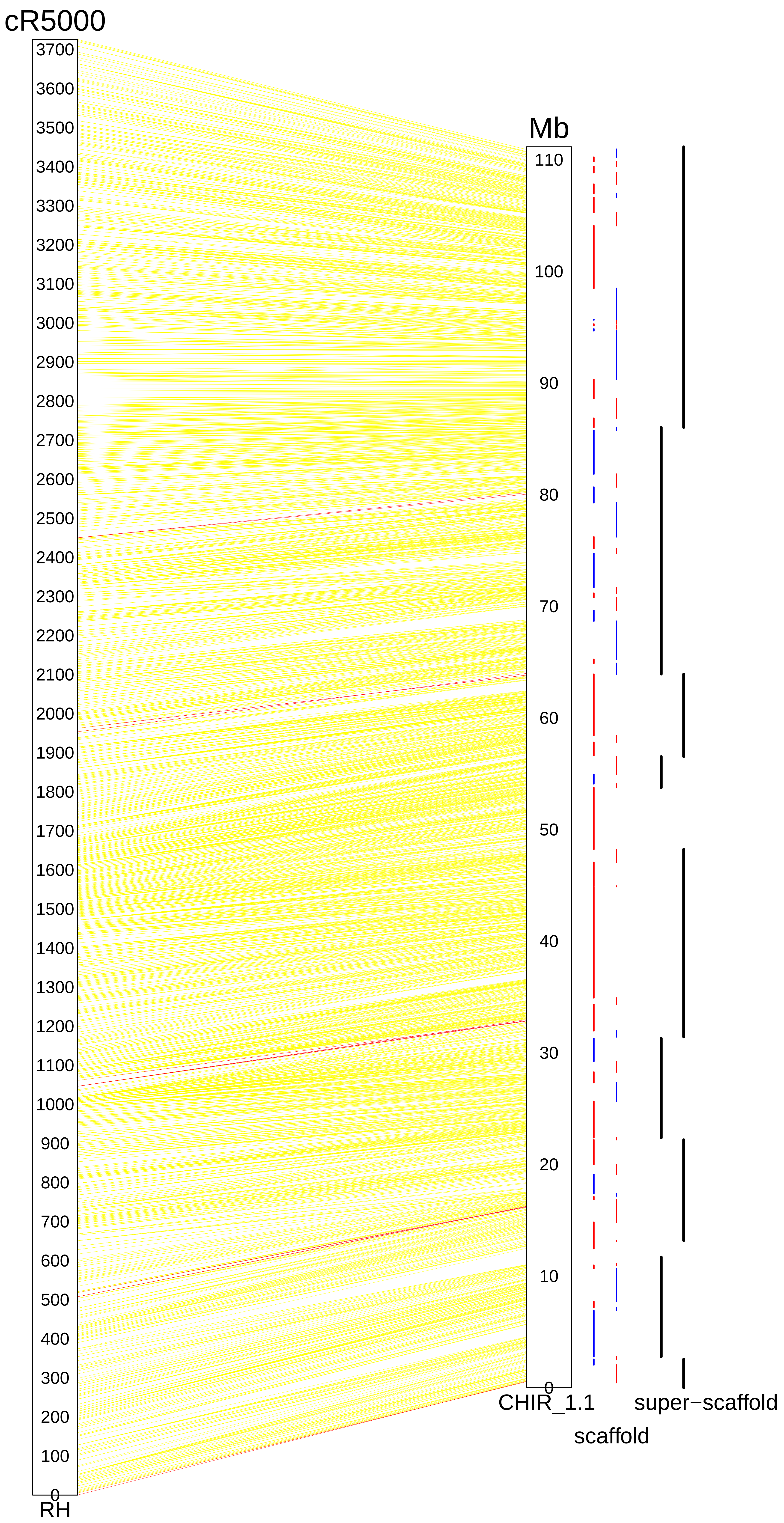

Chromosome 9

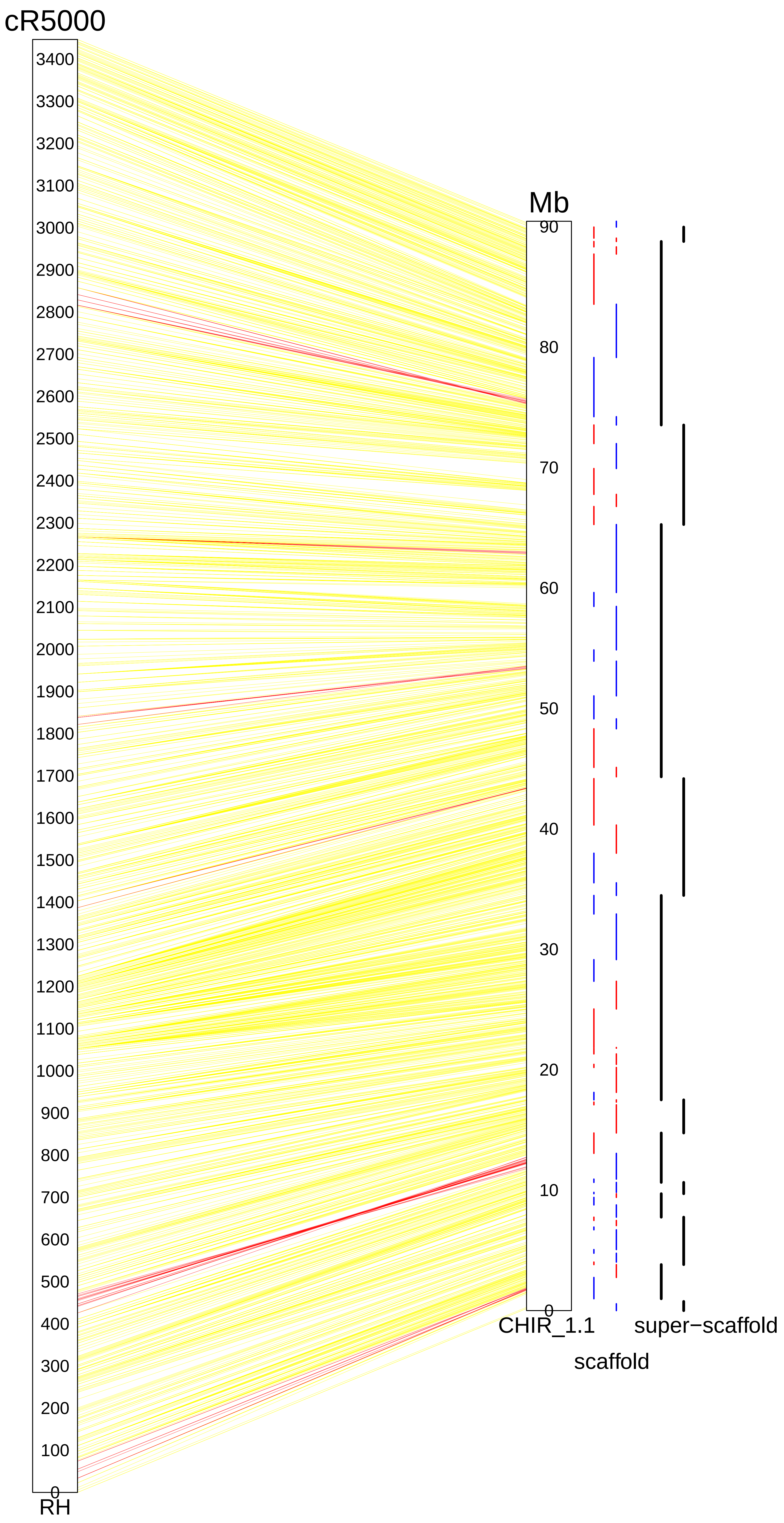

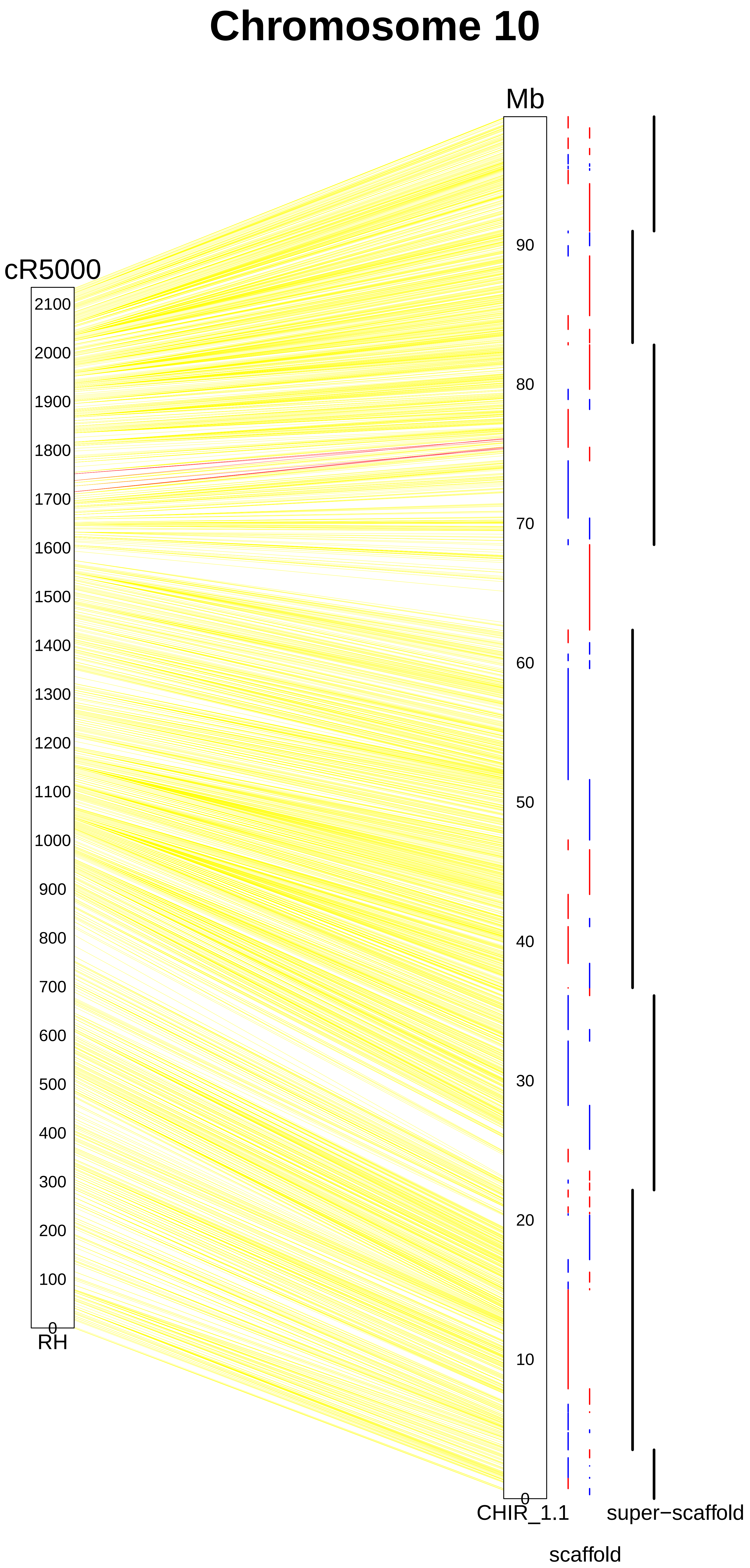

Chromosome 10
cR5000
2100
2000
1900
1800
1700
1600
1500
1400
1300
1200
1100
1000
900
800
700
600
500
400
300
200
100
0
RH
Mb
90
80
70
60
50
40
30
20
10
0
CHIR_1.1
scaffold
super−scaffold

Chromosome 11

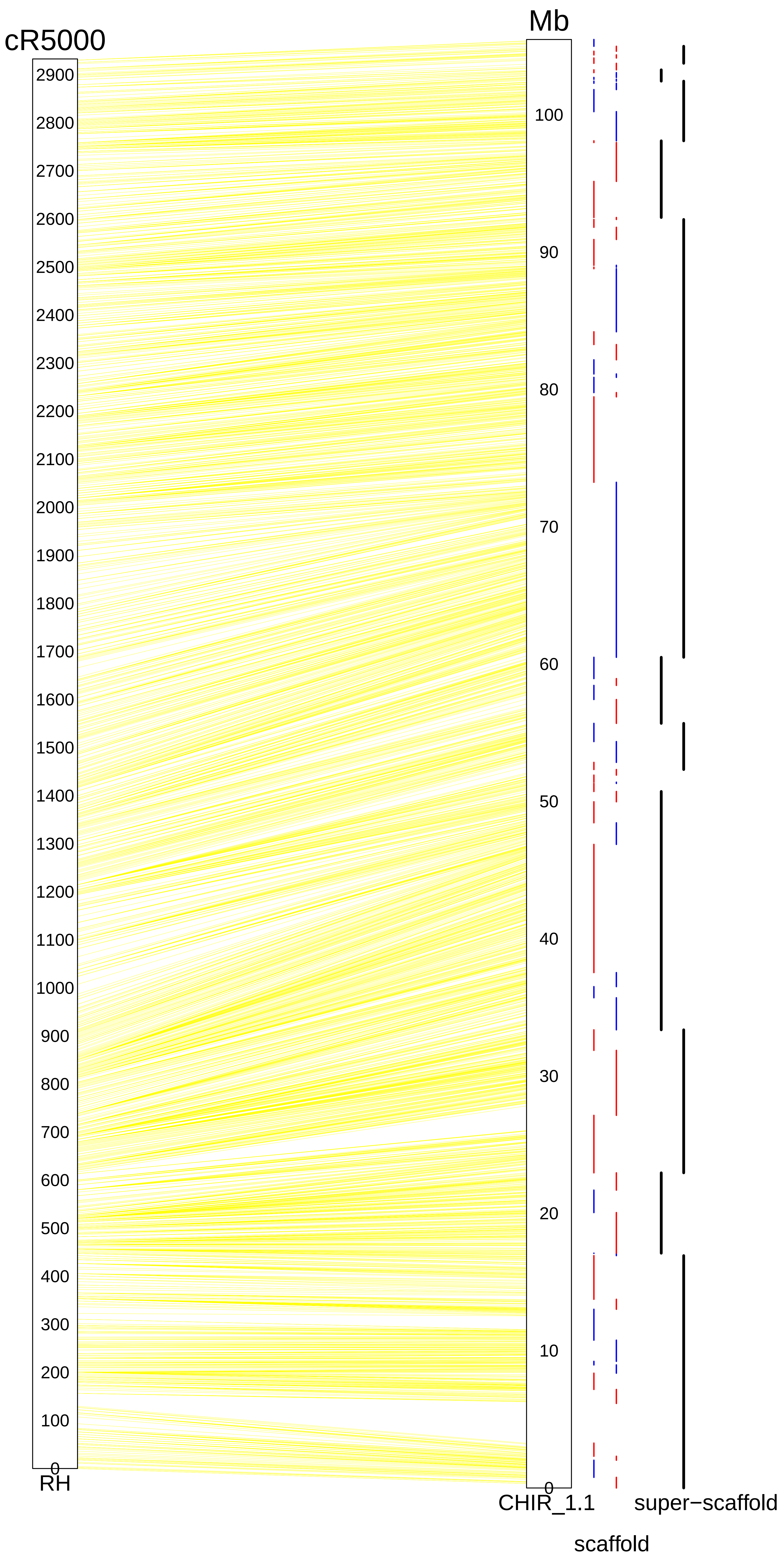

Chromosome 12

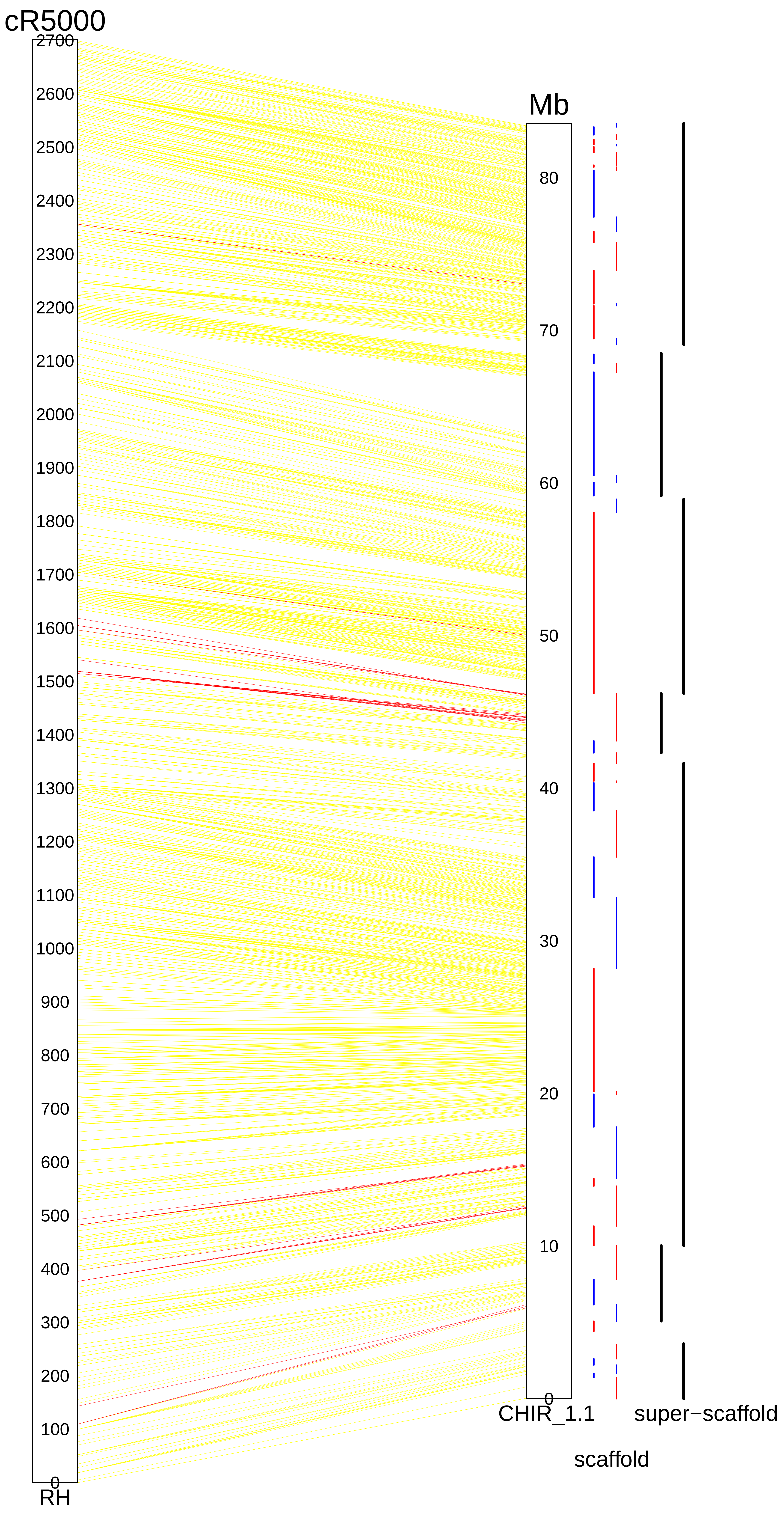

Chromosome 13

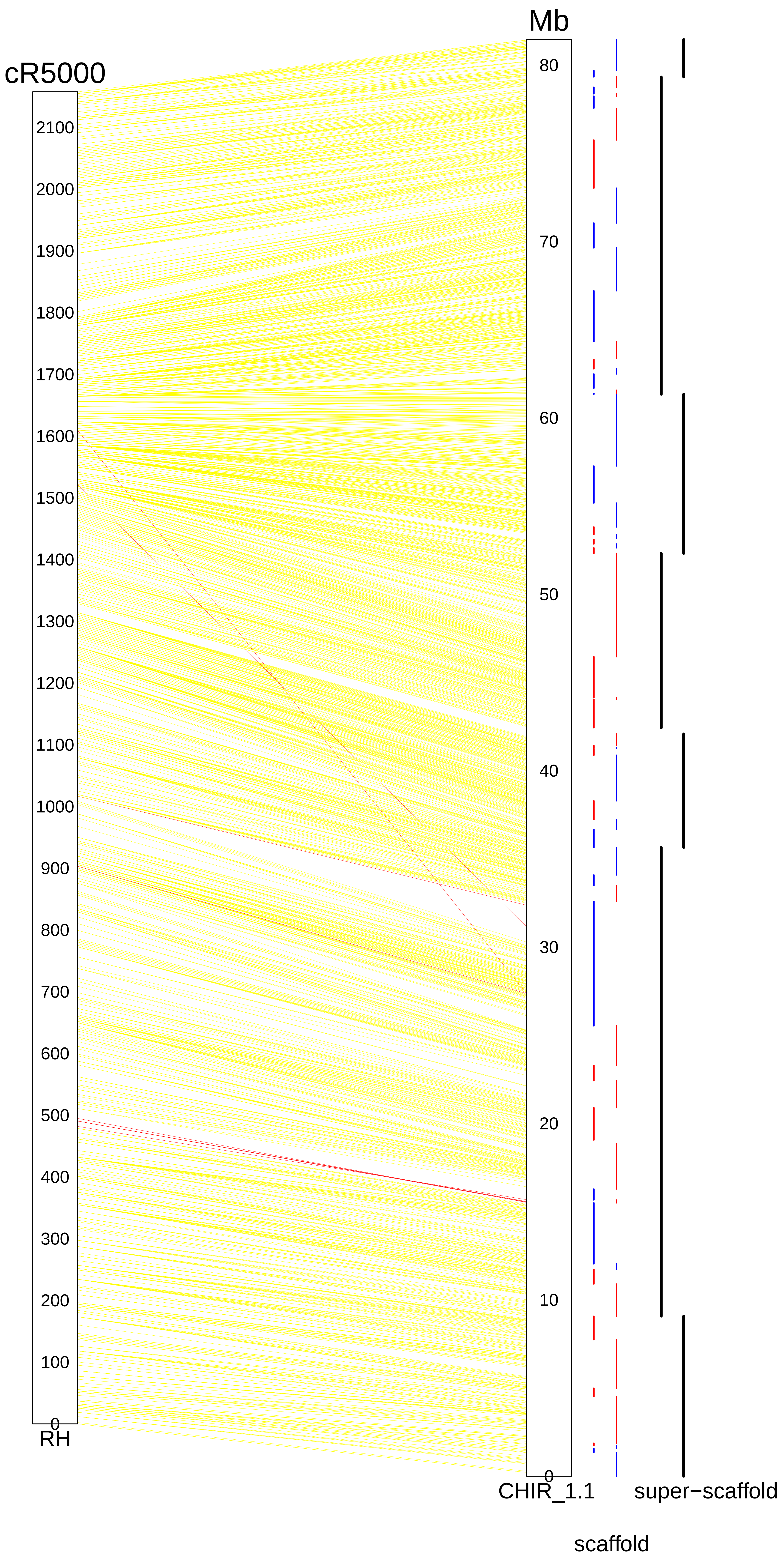

Chromosome 14

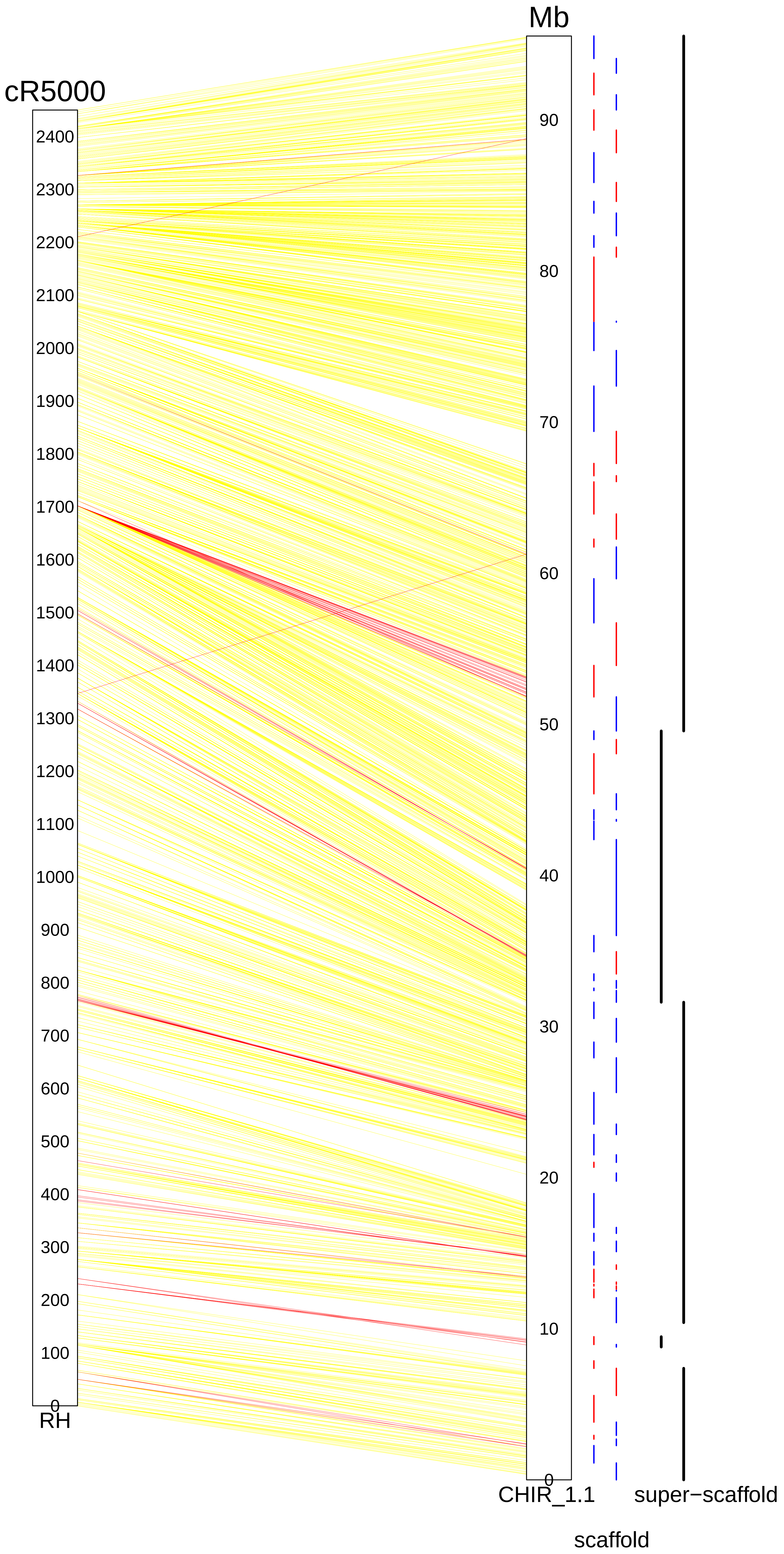

Chromosome 15

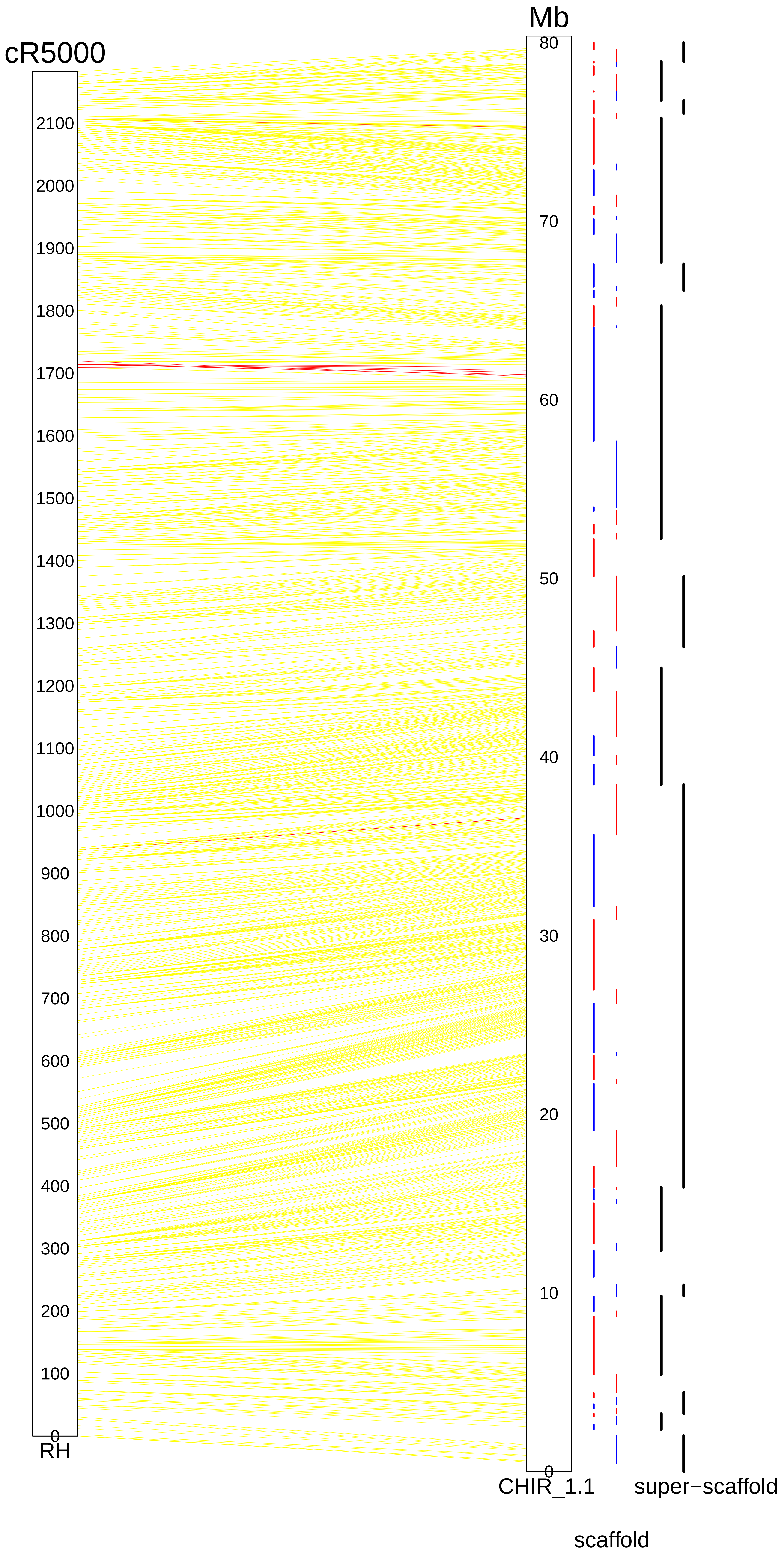

Chromosome 16

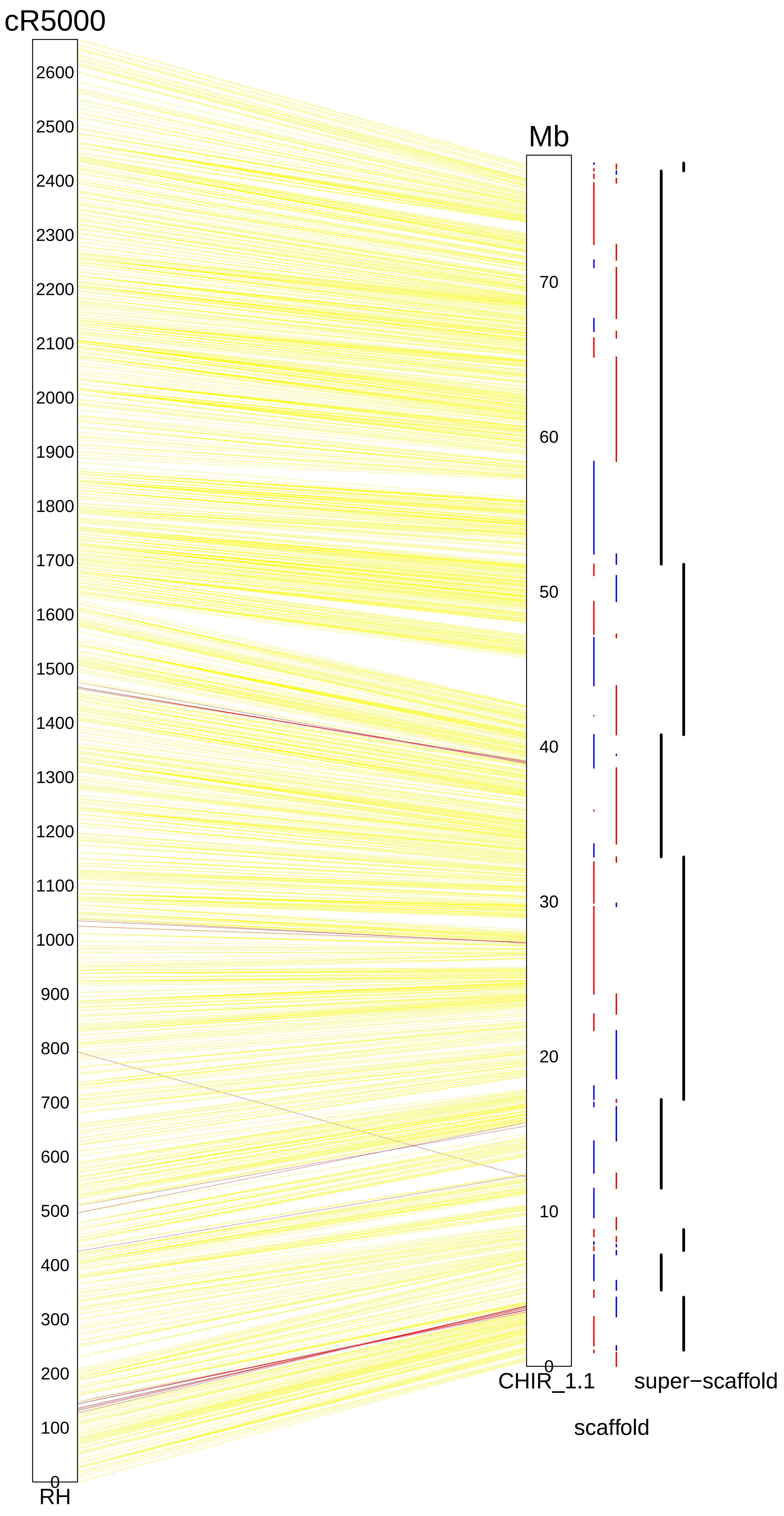

Chromosome 17

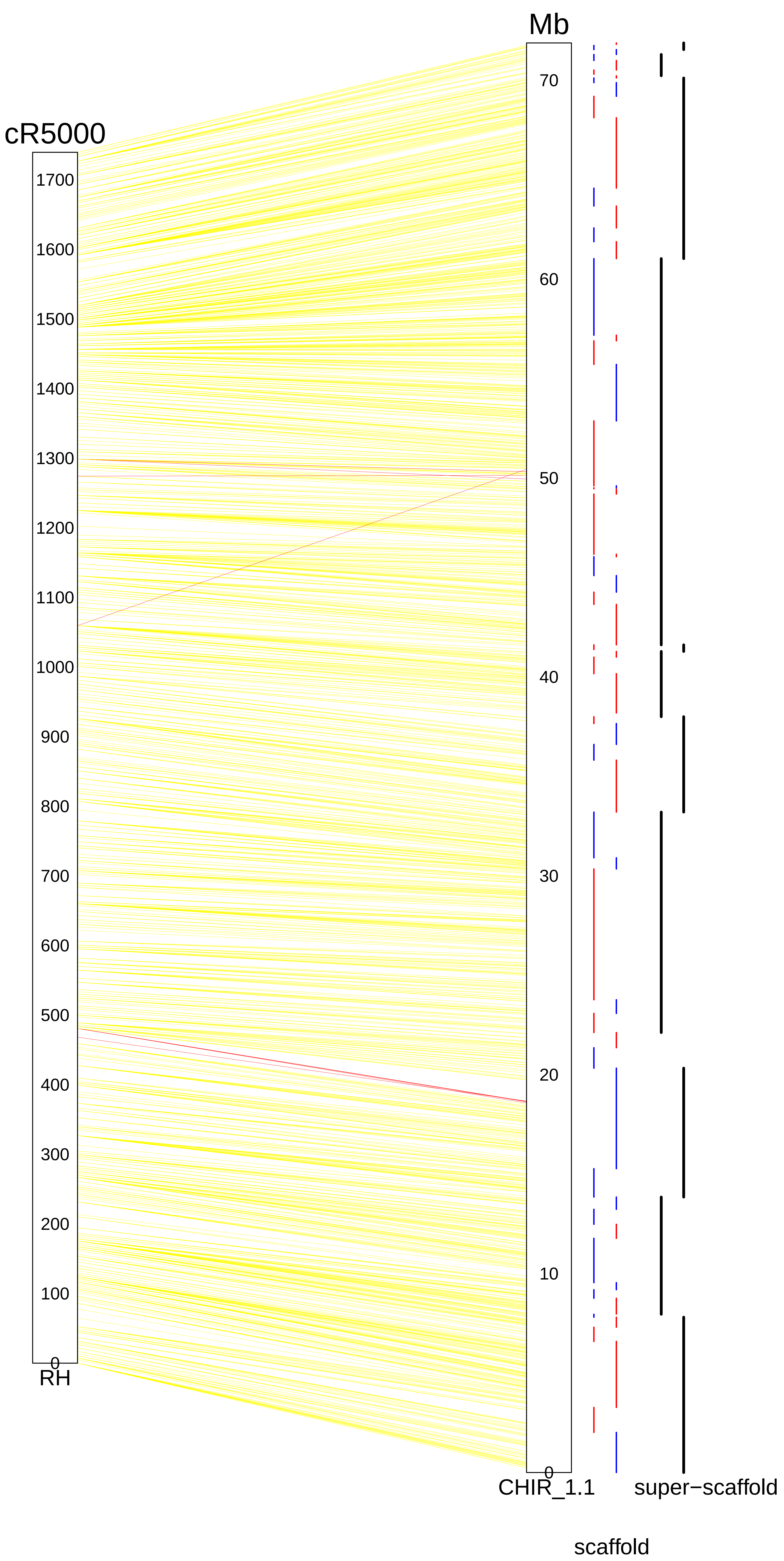

Chromosome 18

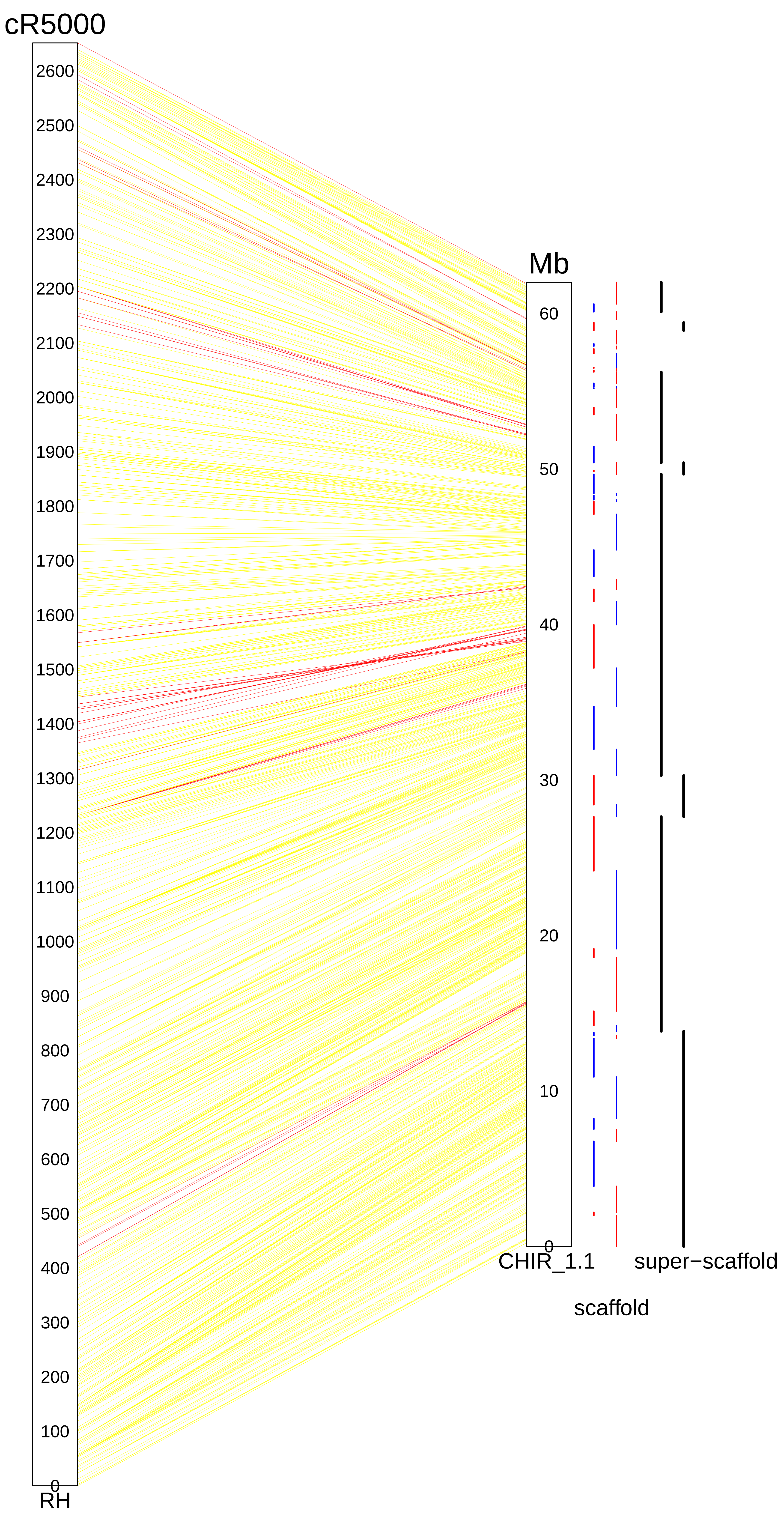

Chromosome 19

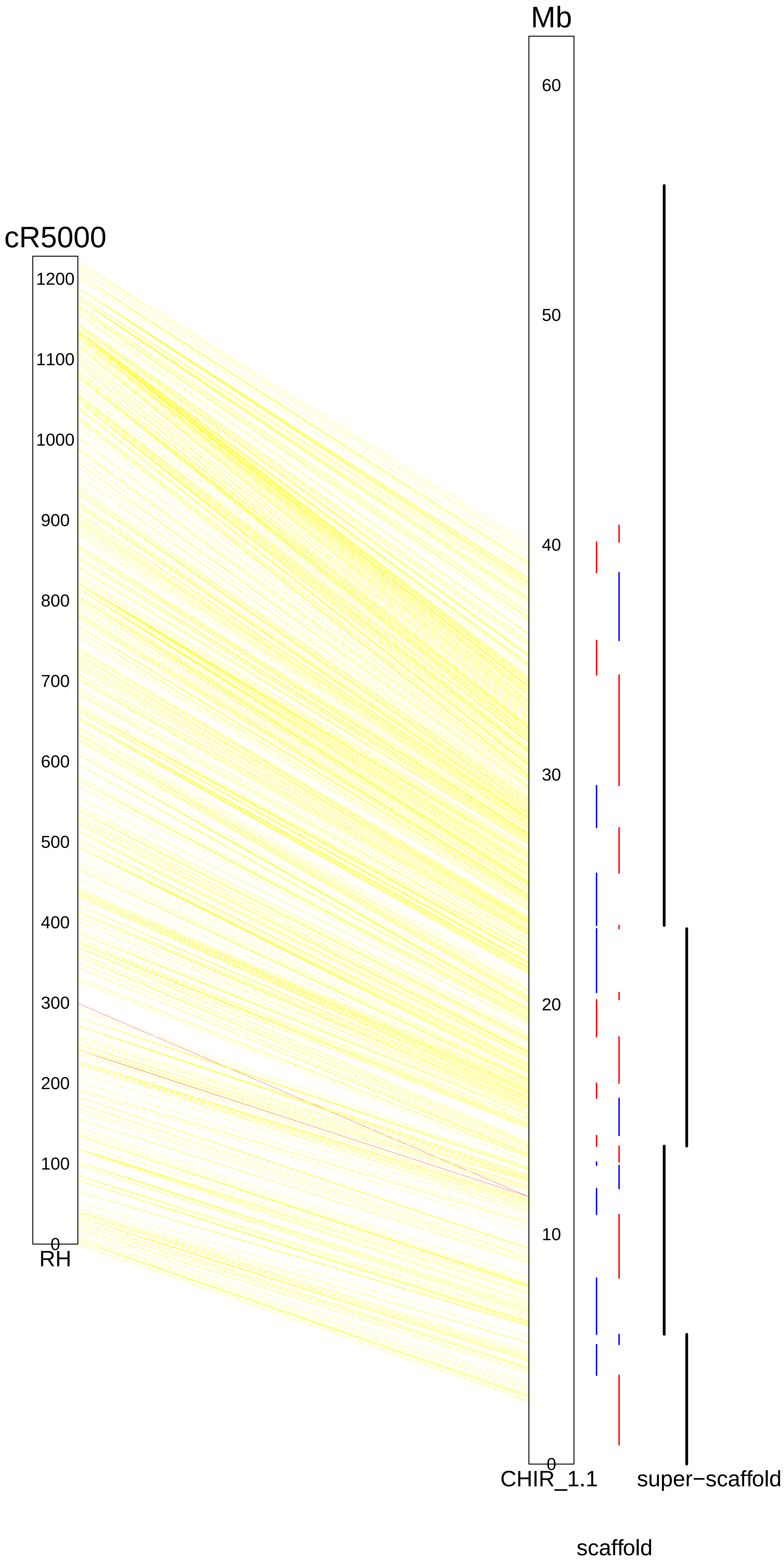

Chromosome 20

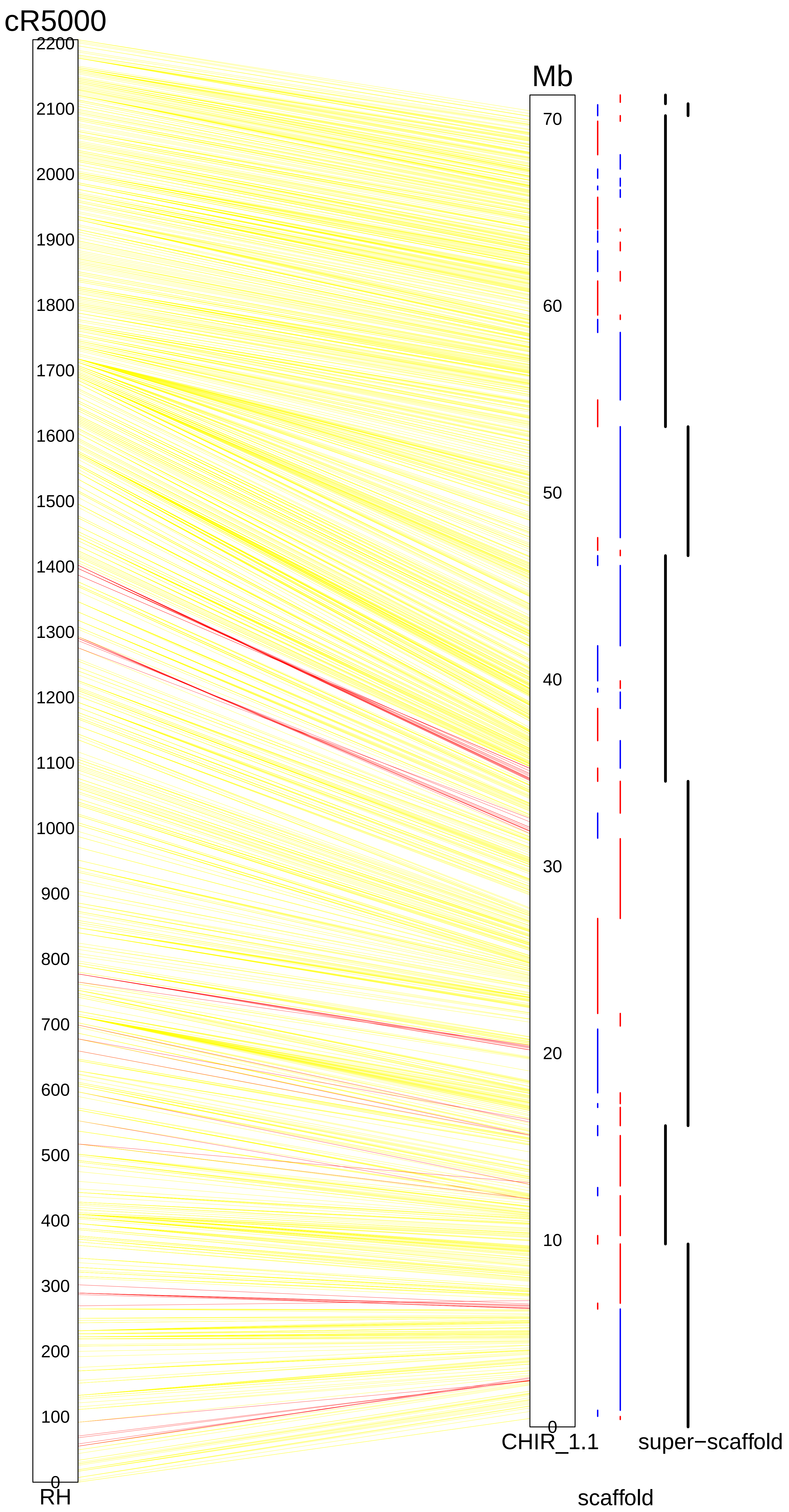

Chromosome 21

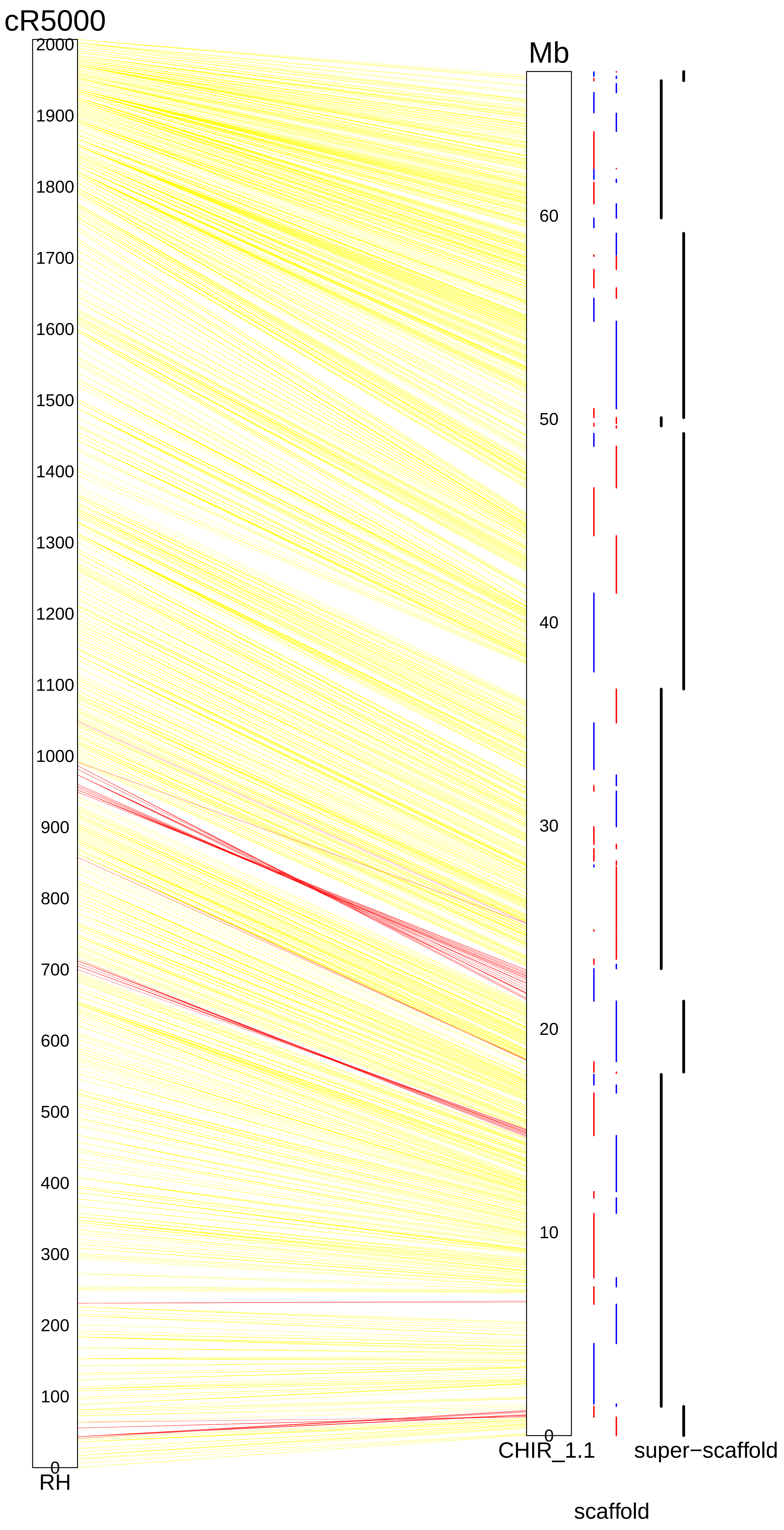

Chromosome 22

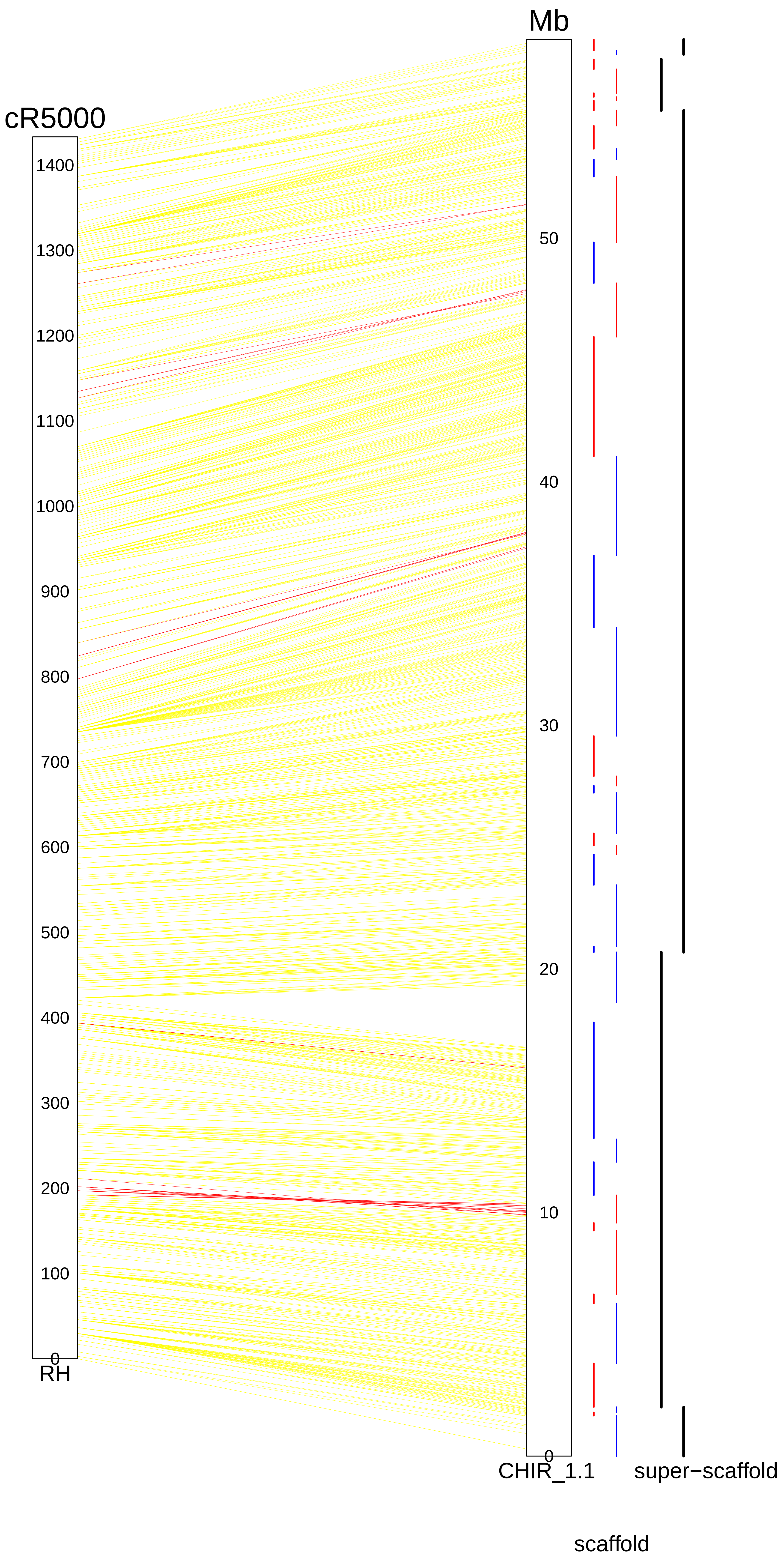

Chromosome 23

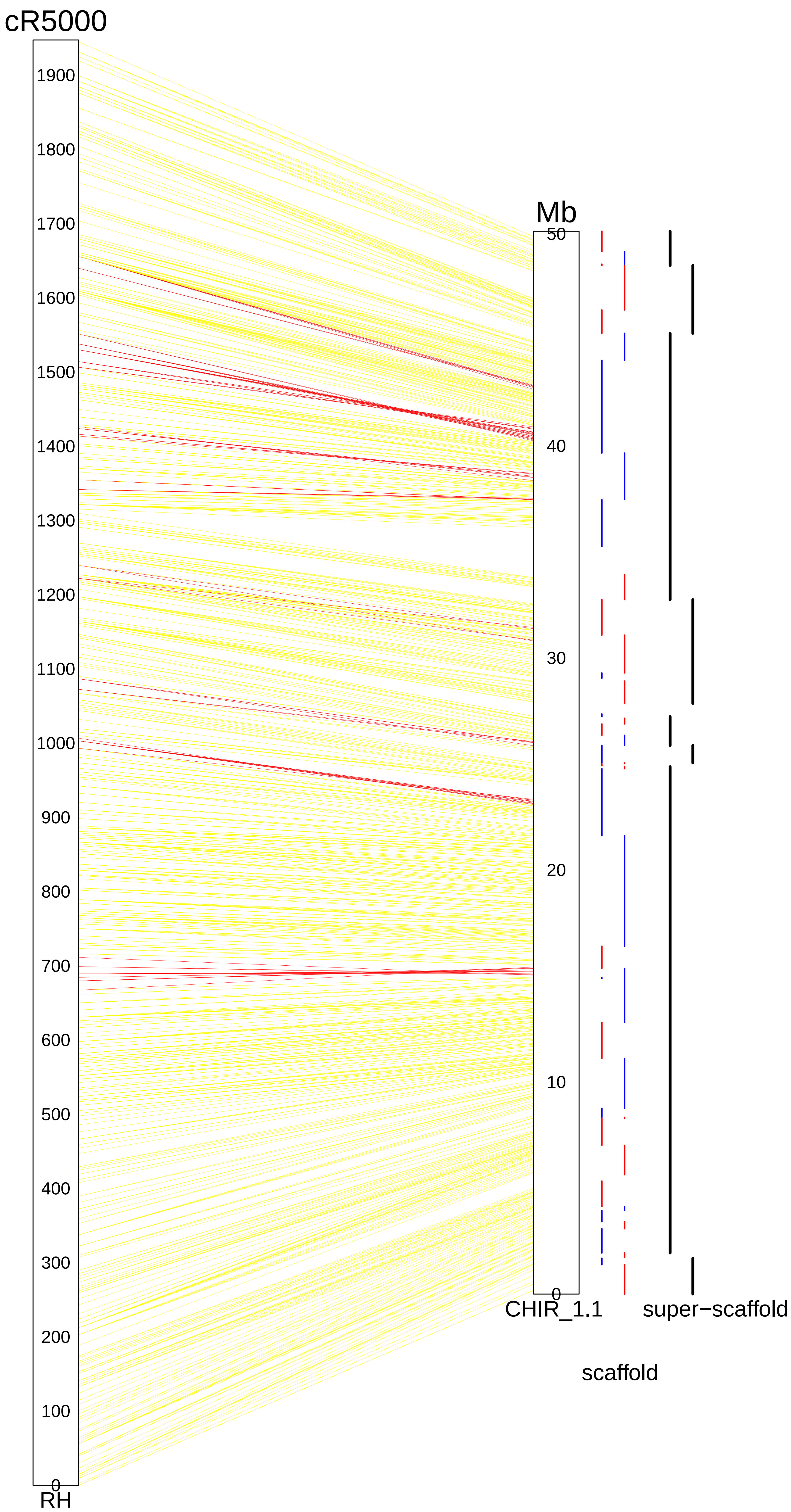

Chromosome 24

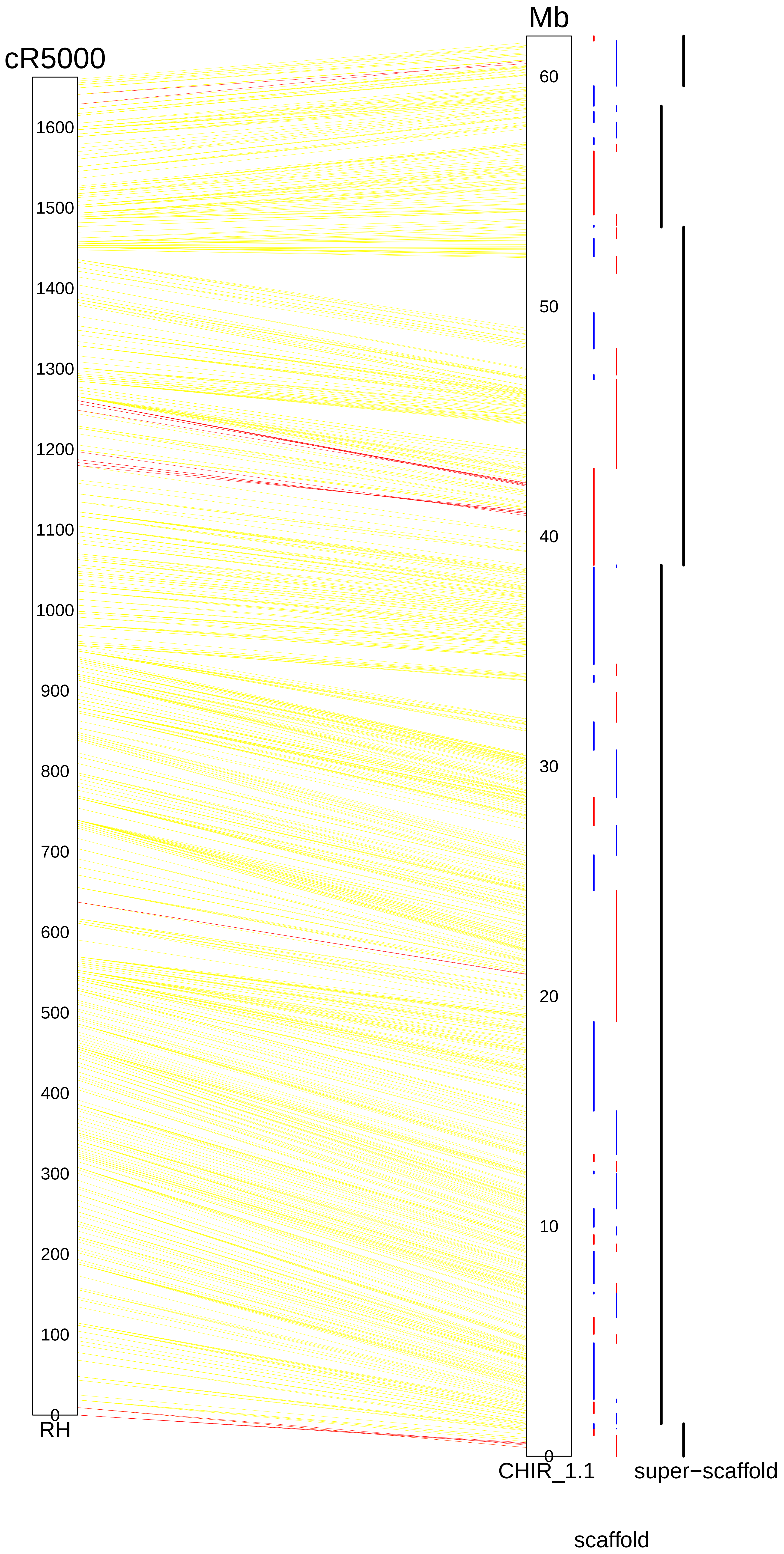

Chromosome 25

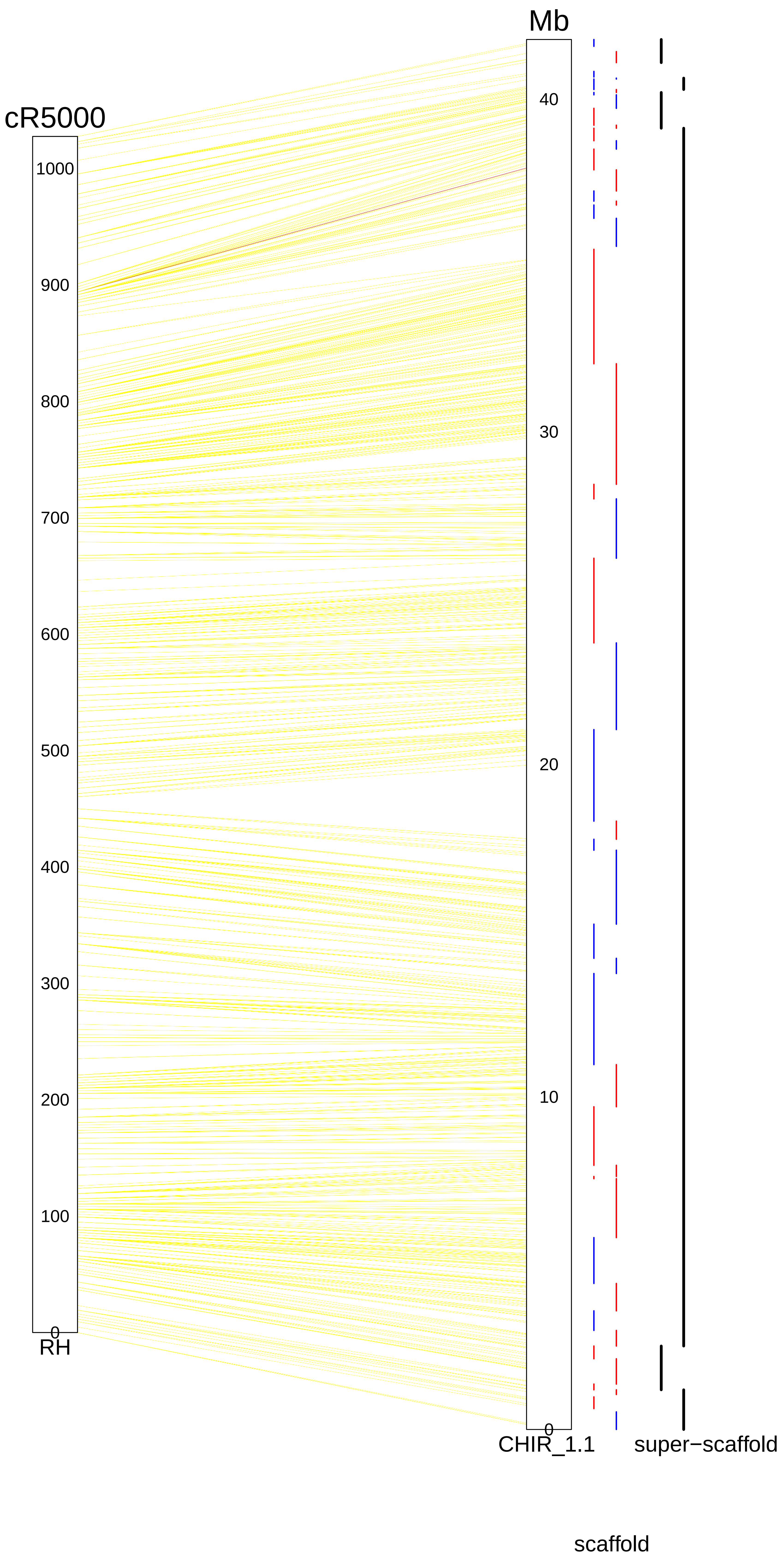

Chromosome 26

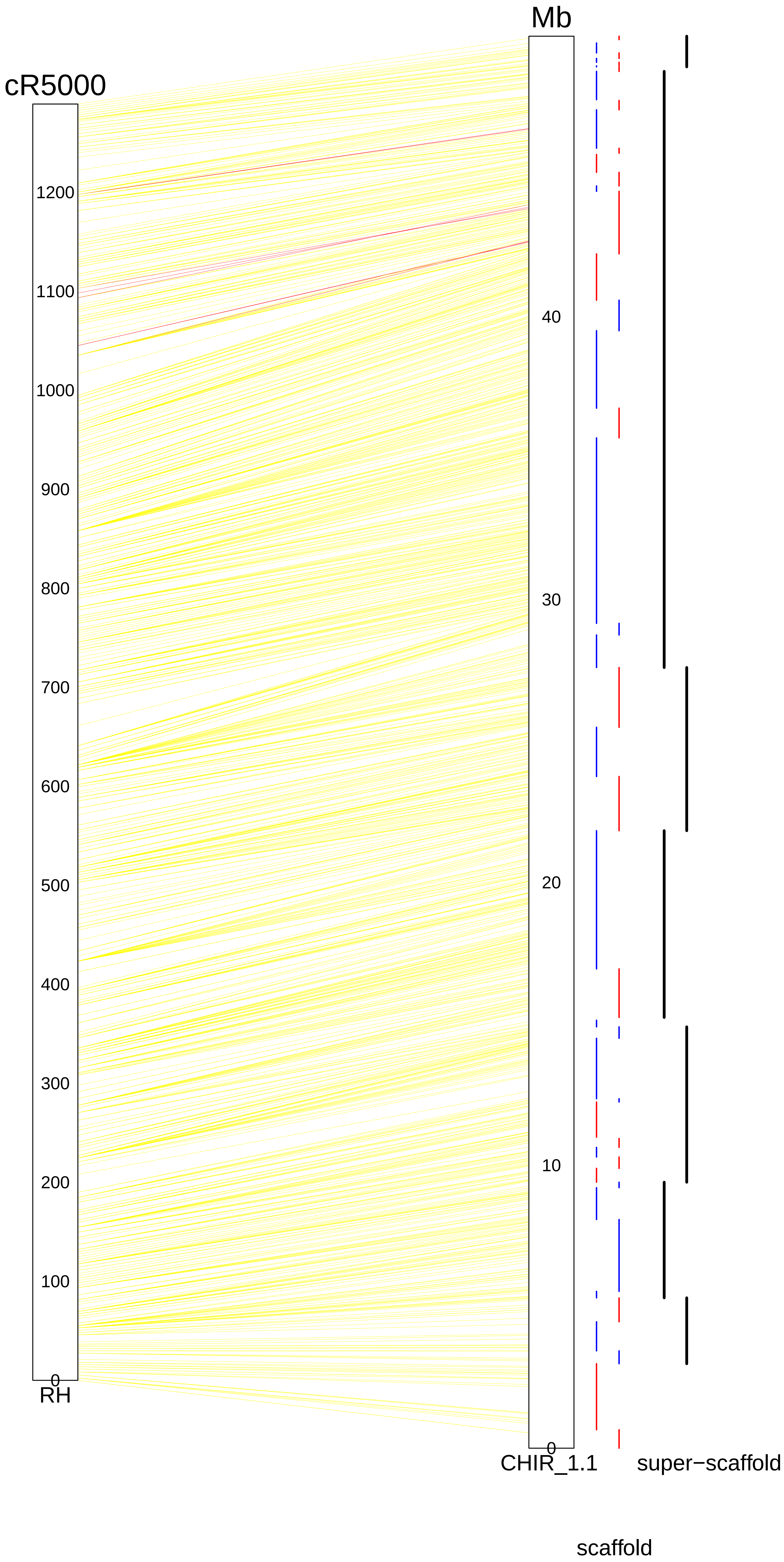

Chromosome 27

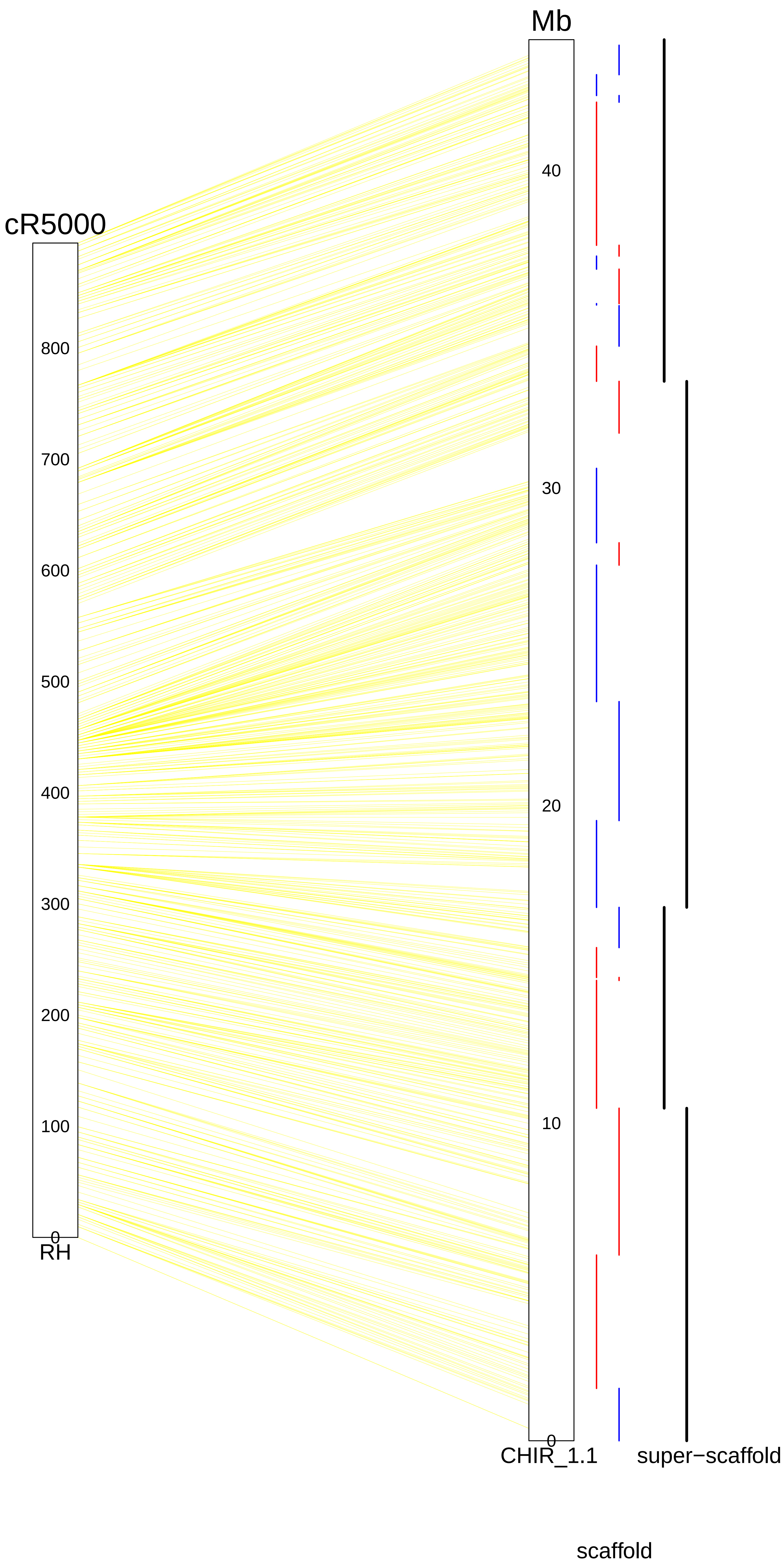

Chromosome 28

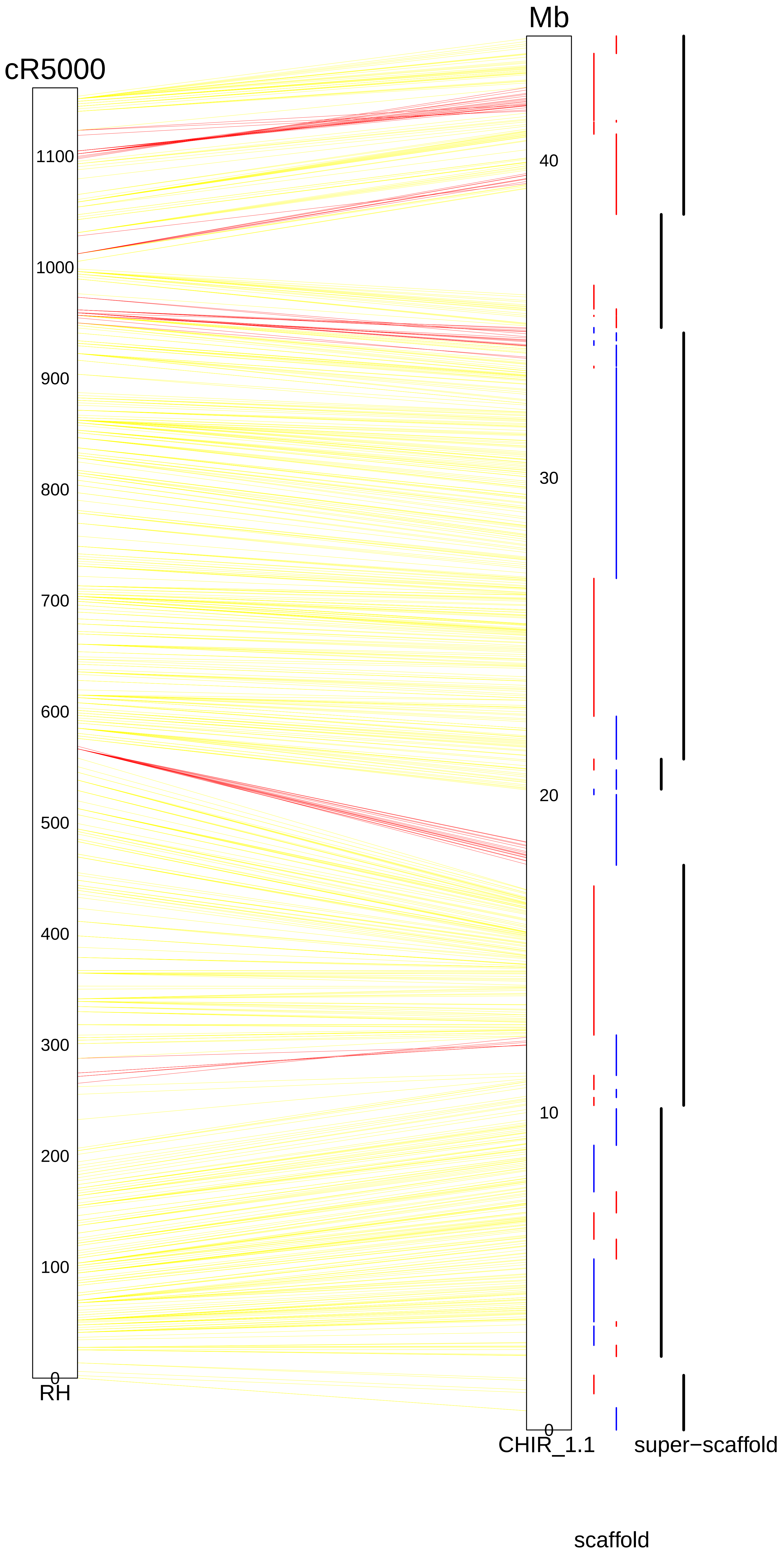

Chromosome 29

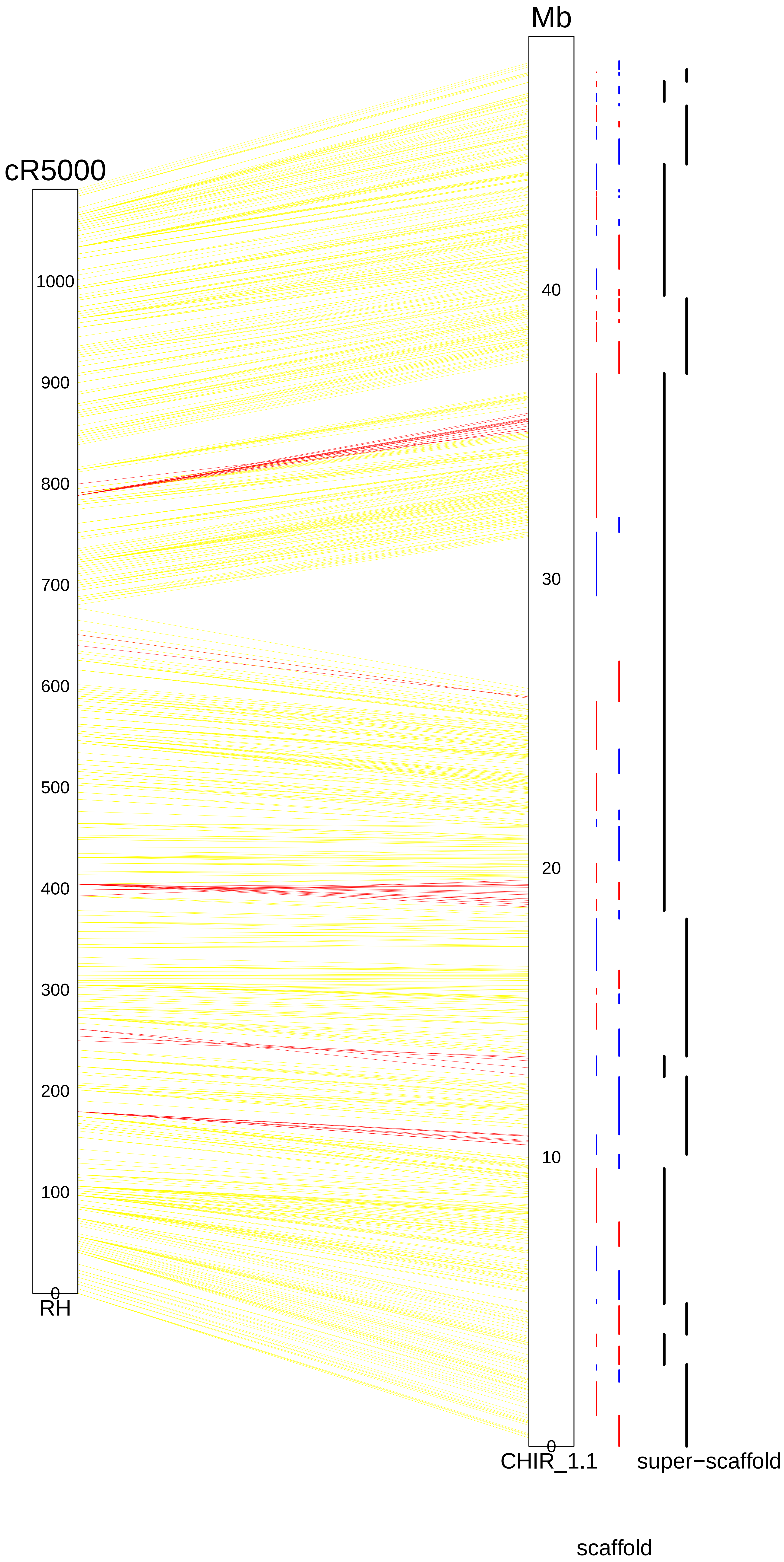

Chromosome X

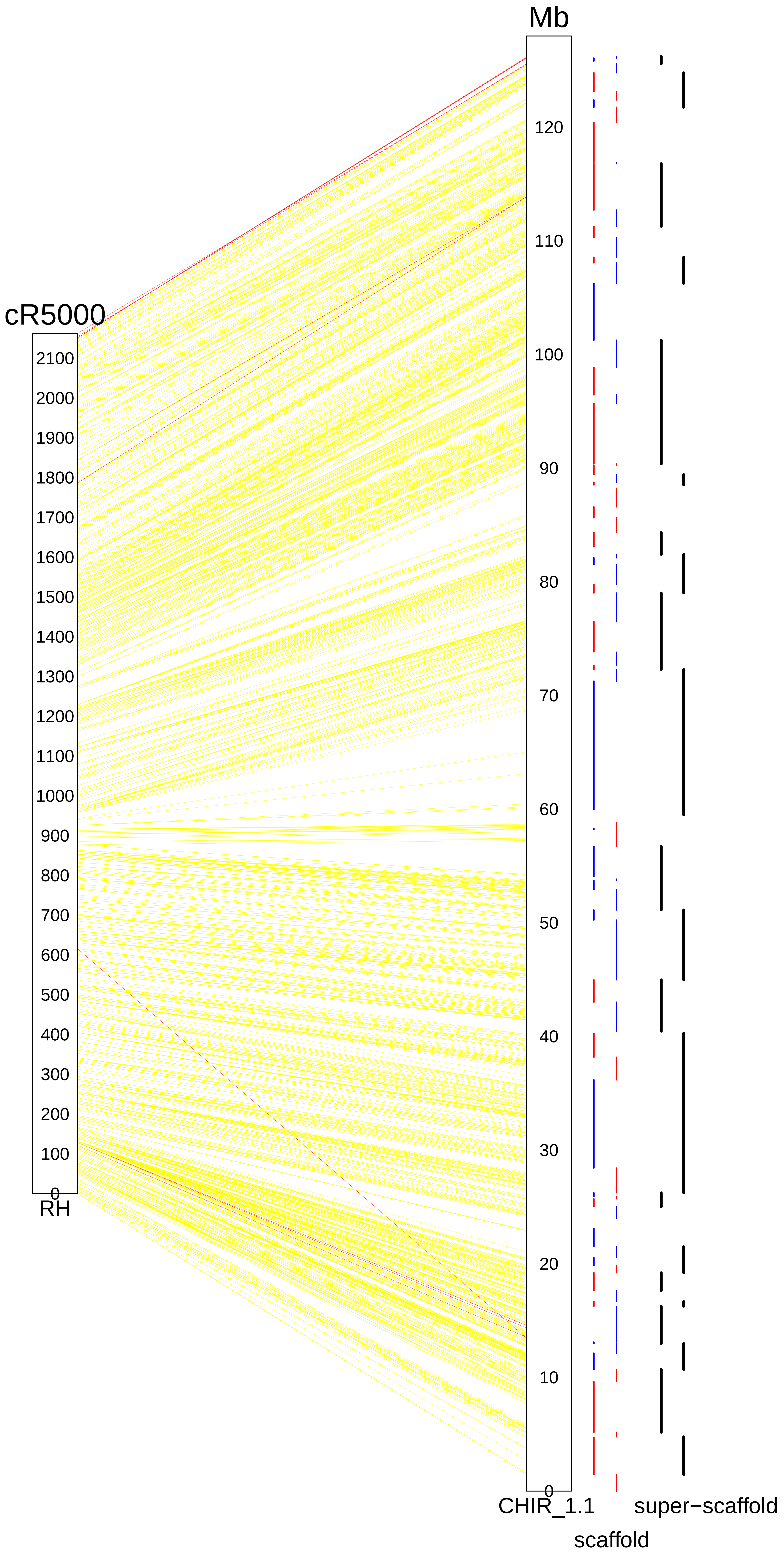

Supplement: Supplementary file 5 — Additional file 5: The connections between the robust RH maps with sequence-based scaffolds and super-scaffolds. Markers in the 30 robust RH maps were linked to 1884 scaffolds and then to 297 super-scaffolds, that were used to reassembling chromosome sequences. Only a few rearrangements (the red link lines between RH markers with chromosome sequences) exist between the RH map and the new assembly CHIR_1.1. Forward alignments between scaffolds and chromosome sequences (CHIR_1.1) are plotted as red lines/dots while inverse (reverse compliment) alignments are plotted as blue lines/dots. Forward alignments between super-scaffolds and CHIR_1.1 are also plotted. To better display the connection, each of neighboring scaffolds (or super-scaffolds) are compulsively divided to two paralleled lines. (PDF 342 KB) [file 12864_2013_6362_MOESM5_ESM.pdf]
