# Supplementary material for: An update of the goat genome assembly using dense radiation hybrid maps allows detailed analysis of evolutionary rearrangements in Bovidae
Source: BMC Genomics. 2014 Jul 23;15(1):625. doi: 10.1186/1471-2164-15-625 (PMC4141111; doi:10.1186/1471-2164-15-625)
Supplement: Supplementary file 9 — Additional file 9: Fast identification of conserved domains in protein sequences of human ANKRD26 homologs was performed using NCBI Conserved Domain Database. Ankyrin domains are near to N-terminal and structural maintenance of chromosomes protein (SMC) domains are near to C-terminal. (PDF 411 KB) [file 12864_2013_6362_MOESM9_ESM.pdf]

Conserved domains on [gi|154354990|ref|NP_055730|]

View concise result

ankyrin repeat domain-containing protein 26 isoform 1 [Homo sapiens]

Graphical summary show options »

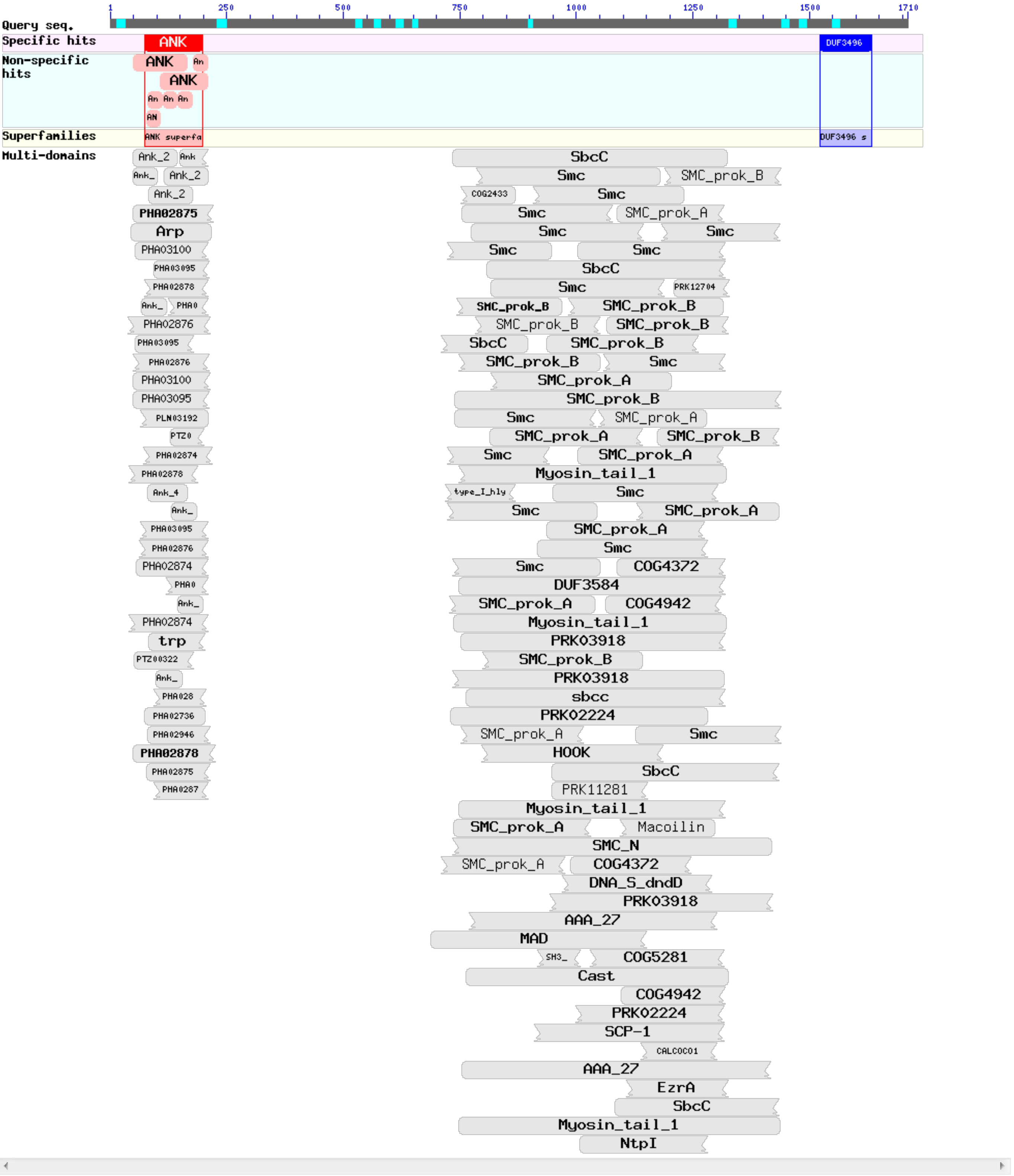

Search for similar domain architectures | Refine search

List of domain hits

Description	PssmId	Multi-dom	E-value
ANK[cd00204], ankyrin repeats; ankyrin repeats mediate protein-protein interactions in very diverse ...	238125	yes	3.80e-37
DUF3496[pfam12001], Domain of unknown function (DUF3496); This presumed domain is functionally uncharacterized. This domain is found i	192910	no	2.62e-36
ANK[cd00204], ankyrin repeats; ankyrin repeats mediate protein-protein interactions in very diverse ...	238125	yes	1.35e-29
ANK[cd00204], ankyrin repeats; ankyrin repeats mediate protein-protein interactions in very diverse ...	238125	yes	7.89e-26
Ank[pfam00023], Ankyrin repeat; Ankyrins are multifunctional adaptors that link specific proteins to the ...	200936	no	5.38e-06
Ank[pfam00023], Ankyrin repeat; Ankyrins are multifunctional adaptors that link specific proteins to the ...	200936	no	1.85e-04
Ank[pfam00023], Ankyrin repeat; Ankyrins are multifunctional adaptors that link specific proteins to the ...	200936	no	2.22e-04

Ank[pfam00023], Ankyrin repeat; Ankyrins are multifunctional adaptors that link specific proteins to the ...	200936	no	2.22e-04
Ank[pfam00023], Ankyrin repeat; Ankyrins are multifunctional adaptors that link specific proteins to the ...	200936	no	2.26e-03
ANK[smart00248], ankyrin repeats; Ankyrin repeats are about 33 amino acids long and occur in at least four consecutive ...	197603	no	5.01e-03
Ank_2[pfam12796], Ankyrin repeats (3 copies);	205076	yes	1.53e-20
Ank_2[pfam12796], Ankyrin repeats (3 copies);	205076	yes	6.42e-16
Ank_2[pfam12796], Ankyrin repeats (3 copies);	205076	yes	1.84e-20
SbcC[COG0419], ATPase involved in DNA repair [DNA replication, recombination, and repair]	223496	yes	1.46e-15
PHA02875[PHA02875], ankyrin repeat protein; Provisional	165206	yes	3.87e-12
Arp[COG0666], FOG: Ankyrin repeat [General function prediction only]	223738	yes	6.28e-12
PHA03100[PHA03100], ankyrin repeat protein; Provisional	222984	yes	8.25e-12
Smc[COG1196], Chromosome segregation ATPases [Cell division and chromosome partitioning]	224117	yes	9.47e-12
PHA03095[PHA03095], ankyrin-like protein; Provisional	222980	yes	1.57e-11
PHA02878[PHA02878], ankyrin repeat protein; Provisional	222939	yes	2.63e-11
Smc[COG1196], Chromosome segregation ATPases [Cell division and chromosome partitioning]	224117	yes	1.15e-10
Smc[COG1196], Chromosome segregation ATPases [Cell division and chromosome partitioning]	224117	yes	1.18e-10
Smc[COG1196], Chromosome segregation ATPases [Cell division and chromosome partitioning]	224117	yes	1.25e-10
PHA02878[PHA02878], ankyrin repeat protein; Provisional	222939	yes	2.83e-10
Smc[COG1196], Chromosome segregation ATPases [Cell division and chromosome partitioning]	224117	yes	3.66e-10
PHA02876[PHA02876], ankyrin repeat protein; Provisional	165207	yes	5.64e-10
PHA03095[PHA03095], ankyrin-like protein; Provisional	222980	yes	1.07e-09
PHA02876[PHA02876], ankyrin repeat protein; Provisional	165207	yes	2.28e-09
SbcC[COG0419], ATPase involved in DNA repair [DNA replication, recombination, and repair]	223496	yes	2.35e-09
Smc[COG1196], Chromosome segregation ATPases [Cell division and chromosome partitioning]	224117	yes	2.50e-09
SMC_prok_B[TIGR02168], chromosome segregation protein SMC, common bacterial type; SMC (structural maintenance of chromosomes) prot	233757	yes	3.04e-09
PHA03100[PHA03100], ankyrin repeat protein; Provisional	222984	yes	3.70e-09
SMC_prok_B[TIGR02168], chromosome segregation protein SMC, common bacterial type; SMC (structural maintenance of chromosomes) prot	233757	yes	6.13e-09
PHA03095[PHA03095], ankyrin-like protein; Provisional	222980	yes	6.26e-09
PLN03192[PLN03192], Voltage-dependent potassium channel; Provisional	215625	yes	1.05e-08
SMC_prok_B[TIGR02168], chromosome segregation protein SMC, common bacterial type; SMC (structural maintenance of chromosomes) prot	233757	yes	1.08e-08
SMC_prok_B[TIGR02168], chromosome segregation protein SMC, common bacterial type; SMC (structural maintenance of chromosomes) prot	233757	yes	1.31e-08
PTZ00322[PTZ00322], 6-phosphofructo-2-kinase/fructose-2,6-biphosphatase; Provisional	140343	yes	1.59e-08
SMC_prok_A[TIGR02169], chromosome segregation protein SMC, primarily archaeal type; SMC (structural maintenance of chromosomes) prote	233758	yes	2.22e-08
SMC_prok_B[TIGR02168], chromosome segregation protein SMC, common bacterial type; SMC (structural maintenance of chromosomes) prot	233757	yes	2.55e-08
SMC_prok_B[TIGR02168], chromosome segregation protein SMC, common bacterial type; SMC (structural maintenance of chromosomes) prot	233757	yes	2.75e-08
SMC_prok_A[TIGR02169], chromosome segregation protein SMC, primarily archaeal type; SMC (structural maintenance of chromosomes) prote	233758	yes	2.78e-08
PHA02874[PHA02874], ankyrin repeat protein; Provisional	165205	yes	2.88e-08
Smc[COG1196], Chromosome segregation ATPases [Cell division and chromosome partitioning]	224117	yes	4.96e-08
SMC_prok_A[TIGR02169], chromosome segregation protein SMC, primarily archaeal type; SMC (structural maintenance of chromosomes) prote	233758	yes	5.00e-08
SMC_prok_A[TIGR02169], chromosome segregation protein SMC, primarily archaeal type; SMC (structural maintenance of chromosomes) prote	233758	yes	5.28e-08
Myosin_tail_1[pfam01576], Myosin tail; The myosin molecule is a multi-subunit complex made up of two heavy chains and four light ...	144972	yes	5.54e-08
Smc[COG1196], Chromosome segregation ATPases [Cell division and chromosome partitioning]	224117	yes	6.95e-08
Smc[COG1196], Chromosome segregation ATPases [Cell division and chromosome partitioning]	224117	yes	9.26e-08
PHA02878[PHA02878], ankyrin repeat protein; Provisional	222939	yes	9.96e-08
Ank_5[pfam13857], Ankyrin repeats (many copies);	206028	yes	1.21e-07
Ank_4[pfam13637], Ankyrin repeats (many copies);	222277	yes	1.55e-07
SMC_prok_A[TIGR02169], chromosome segregation protein SMC, primarily archaeal type; SMC (structural maintenance of chromosomes) prote	233758	yes	1.98e-07
Smc[COG1196], Chromosome segregation ATPases [Cell division and chromosome partitioning]	224117	yes	2.60e-07
Ank_4[pfam13637], Ankyrin repeats (many copies);	222277	yes	3.15e-07
Ank_2[pfam12796], Ankyrin repeats (3 copies);	205076	yes	3.34e-07
Smc[COG1196], Chromosome segregation ATPases [Cell division and chromosome partitioning]	224117	yes	3.77e-07
Ank_5[pfam13857], Ankyrin repeats (many copies);	206028	yes	4.08e-07
Smc[COG1196], Chromosome segregation ATPases [Cell division and chromosome partitioning]	224117	yes	4.46e-07
DUF3584[pfam12128], Protein of unknown function (DUF3584); This protein is found in bacteria and eukaryotes. Proteins in this family are typica	221432	yes	5.64e-07
PHA03095[PHA03095], ankyrin-like protein; Provisional	222980	yes	5.90e-07
SMC_prok_A[TIGR02169], chromosome segregation protein SMC, primarily archaeal type; SMC (structural maintenance of chromosomes) prote	233758	yes	7.18e-07
Myosin_tail_1[pfam01576], Myosin tail; The myosin molecule is a multi-subunit complex made up of two heavy chains and four light ...	144972	yes	8.03e-07
PHA02876[PHA02876], ankyrin repeat protein; Provisional	165207	yes	9.49e-07
PRK03918[PRK03918], chromosome segregation protein; Provisional	235175	yes	1.01e-06
SMC_prok_A[TIGR02169], chromosome segregation protein SMC, primarily archaeal type; SMC (structural maintenance of chromosomes) prote	233758	yes	1.30e-06
SMC_prok_B[TIGR02168], chromosome segregation protein SMC, common bacterial type; SMC (structural maintenance of chromosomes) prot	233757	yes	1.62e-06
SMC_prok_A[TIGR02169], chromosome segregation protein SMC, primarily archaeal type; SMC (structural maintenance of chromosomes) prote	233758	yes	1.81e-06
PRK03918[PRK03918], chromosome segregation protein; Provisional	235175	yes	2.56e-06
PHA02874[PHA02874], ankyrin repeat protein; Provisional	165205	yes	3.64e-06
sbcc[TIGR00618], exonuclease SbcC; All proteins in this family for which functions are known are part of an exonuclease ...	129705	yes	3.82e-06
PHA02876[PHA02876], ankyrin repeat protein; Provisional	165207	yes	4.43e-06
Ank_4[pfam13637], Ankyrin repeats (many copies);	222277	yes	6.00e-06
PHA02874[PHA02874], ankyrin repeat protein; Provisional	165205	yes	9.92e-06
PRK02224[PRK02224], chromosome segregation protein; Provisional	179385	yes	1.08e-05
trp[TIGR00870], transient-receptor-potential calcium channel protein; The Transient Receptor Potential Ca2+ Channel (TRP-CC) Family (TC. 1.A	233161	yes	1.37e-05
PTZ00322[PTZ00322], 6-phosphofructo-2-kinase/fructose-2,6-biphosphatase; Provisional	140343	yes	2.45e-05
SMC_prok_A[TIGR02169], chromosome segregation protein SMC, primarily archaeal type; SMC (structural maintenance of chromosomes) prote	233758	yes	3.34e-05
HOOK[pfam05622], HOOK protein; This family consists of several HOOK1, 2 and 3 proteins from different eukaryotic ...	218661	yes	4.34e-05
Smc[COG1196], Chromosome segregation ATPases [Cell division and chromosome partitioning]	224117	yes	4.85e-05
SbcC[COG0419], ATPase involved in DNA repair [DNA replication, recombination, and repair]	223496	yes	1.43e-04
PRK11281[PRK11281], hypothetical protein; Provisional	236892	yes	1.43e-04
Ank_5[pfam13857], Ankyrin repeats (many copies);	206028	yes	1.52e-04
COG4372[COG4372], Uncharacterized protein conserved in bacteria with the myosin-like domain [Function ...	226809	yes	1.75e-04
SMC_prok_B[TIGR02168], chromosome segregation protein SMC, common bacterial type; SMC (structural maintenance of chromosomes) prot	233757	yes	2.98e-04
Myosin_tail_1[pfam01576], Myosin tail; The myosin molecule is a multi-subunit complex made up of two heavy chains and four light ...	144972	yes	3.29e-04
SMC_prok_A[TIGR02169], chromosome segregation protein SMC, primarily archaeal type; SMC (structural maintenance of chromosomes) prote	233758	yes	3.30e-04
COG4942[COG4942], Membrane-bound metallopeptidase [Cell division and chromosome partitioning]	227278	yes	3.69e-04
SMC_prok_B[TIGR02168], chromosome segregation protein SMC, common bacterial type; SMC (structural maintenance of chromosomes) prot	233757	yes	3.77e-04
Smc[COG1196], Chromosome segregation ATPases [Cell division and chromosome partitioning]	224117	yes	3.86e-04
Smc[COG1196], Chromosome segregation ATPases [Cell division and chromosome partitioning]	224117	yes	4.22e-04
PHA02874[PHA02874], ankyrin repeat protein; Provisional	165205	yes	4.41e-04
PHA02736[PHA02736], Viral ankyrin protein; Provisional	165103	yes	4.77e-04
SMC_N[pfam02463], RecF/RecN/SMC N terminal domain; This domain is found at the N terminus of SMC proteins. The SMC (structural mainter	217051	yes	4.81e-04
COG2433[COG2433], Uncharacterized conserved protein [Function unknown]	225288	yes	5.01e-04
[illegible]	[illegible]	[illegible]	[illegible]

Macoilin[pfam09726], Transmembrane protein; This entry is a highly conserved protein present in eukaryotes.	220365	yes	6.09e-04
Smc[COG1196], Chromosome segregation ATPases [Cell division and chromosome partitioning]	224117	yes	6.96e-04
COG4372[COG4372], Uncharacterized protein conserved in bacteria with the myosin-like domain [Function ...	226809	yes	7.01e-04
PHA02946[PHA02946], ankyin-like protein; Provisional	165256	yes	7.52e-04
SMC_prok_A[TIGR02169], chromosome segregation protein SMC, primarily archaeal type; SMC (structural maintenance of chromosomes) prote	233758	yes	8.01e-04
PHA02878[PHA02878], ankyrin repeat protein; Provisional	222939	yes	1.01e-03
DNA_S_dndD[TIGR03185], DNA sulfur modification protein DndD; This model describes the DndB protein encoded by an operon associated wi	234141	yes	1.07e-03
PRK03918[PRK03918], chromosome segregation protein; Provisional	235175	yes	1.38e-03
AAA_27[pfam13514], AAA domain; This domain is found in a number of double-strand DNA break proteins. This domain ...	205692	yes	1.38e-03
PHA02875[PHA02875], ankyrin repeat protein; Provisional	165206	yes	1.46e-03
MAD[pfam05557], Mitotic checkpoint protein; This family consists of several eukaryotic mitotic checkpoint (Mitotic arrest deficient ...	218636	yes	1.71e-03
COG5281[COG5281], Phage-related minor tail protein [Function unknown]	227606	yes	1.87e-03
Cast[pfam10174], RIM-binding protein of the cytomatrix active zone; This is a family of proteins that form part of the CAZ (cytomatrix at the active z	220614	yes	2.28e-03
COG4942[COG4942], Membrane-bound metallopeptidase [Cell division and chromosome partitioning]	227278	yes	2.85e-03
PHA02876[PHA02876], ankyrin repeat protein; Provisional	165207	yes	3.15e-03
PRK02224[PRK02224], chromosome segregation protein; Provisional	179385	yes	3.46e-03
SCP-1[pfam05483], Synaptonemal complex protein 1 (SCP-1); Synaptonemal complex protein 1 (SCP-1) is the major component of the transver	114219	yes	3.53e-03
SMC_prok_B[TIGR02168], chromosome segregation protein SMC, common bacterial type; SMC (structural maintenance of chromosomes) prot	233757	yes	3.83e-03
CALCOCO1[pfam07888], Calcium binding and coiled-coil domain (CALCOCO1) like; Proteins found in this family are similar to the coiled-coil tr	116500	yes	4.13e-03
AAA_27[pfam13514], AAA domain; This domain is found in a number of double-strand DNA break proteins. This domain ...	205692	yes	4.36e-03
EzrA[pfam06160], Septation ring formation regulator, EzrA; During the bacterial cell cycle, the tubulin-like cell-division protein FtsZ polymerises ...	114855	yes	4.66e-03
SbcC[COG0419], ATPase involved in DNA repair [DNA replication, recombination, and repair]	223496	yes	4.75e-03
Myosin_tail_1[pfam01576], Myosin tail; The myosin molecule is a multi-subunit complex made up of two heavy chains and four light ...	144972	yes	5.38e-03
NtpI[COG1269], Archaeal/vacuolar-type H+-ATPase subunit I [Energy production and conversion]	224188	yes	5.56e-03
SbcC[COG0419], ATPase involved in DNA repair [DNA replication, recombination, and repair]	223496	yes	6.70e-03
SH3_and_anchor[TIGR04211], SH3 domain protein; Members of this protein family have a signal peptide, a strongly conserved SH3 domain, a	234501	yes	7.58e-03
PRK12704[PRK12704], phosphodiesterase; Provisional	237177	yes	7.65e-03
type_I_hlyD[TIGR01843], type I secretion membrane fusion protein, HlyD family; Type I secretion is an ABC transport process that exports protein	130902	yes	7.86e-03

Blast search parameters

**Data Source:**	Precalculated data, version = cdd.v.3.10
**Preset Options:**	**Database:** cdsearch/cdd **Low complexity filter:** yes **E-value threshold:** 0.01

References:

Marchler-Bauer A et al. (2011), *"CDD: a Conserved Domain Database for the functional annotation of proteins."*, **Nucleic Acids Res.39**(D)225-9.

Marchler-Bauer A et al. (2009), *"CDD: specific functional annotation with the Conserved Domain Database."*, **Nucleic Acids Res.37**(D)205-10.

Marchler-Bauer A, Bryant SH (2004), *"CD-Search: protein domain annotations on the fly."*, **Nucleic Acids Res.32**(W)327-331.

Help | Disclaimer | Write to the Help Desk
NCBI | NLM | NIH
